# Molecular Identification of Culturable Bacteria and *Vibrio* Virulence‐Related Genes Associated With Sea Turtle Eggshells and Nest Sediments in Pangandaran, Indonesia

**DOI:** 10.1155/ijm/7422673

**Published:** 2026-10-05

**Authors:** Titin Herawati, Indriyani Rahayu, Aisyah Aisyah, Mochamad Untung Kurnia Agung, Mochamad Rudyansyah Ismail, Donny Juliandri Prihadi, Eri Bachtiar, Adiana Ghazali, Yudi Nurul Ihsan

**Affiliations:** ^1^ Master of Marine Conservation Universitas Padjadjaran Sumedang West Java Indonesia unpad.ac.id; ^2^ Department of Marine Science Universitas Padjadjaran Sumedang West Java Indonesia unpad.ac.id; ^3^ Doctoral Program in Sustainable Fisheries and Marine Sciences Faculty of Fisheries and Marine Sciences Universitas Padjadjaran Sumedang Indonesia unpad.ac.id; ^4^ Department of Fisheries Universitas Padjadjaran Sumedang West Java Indonesia unpad.ac.id; ^5^ Marine Science Faculty of Science & Marine Environment Universiti Malaysia Terengganu Kuala Nerus Terengganu Malaysia umt.edu.my

**Keywords:** bacterial contamination, conservation, sea turtle eggs, *toxin* gene, zoonotic risk

## Abstract

Microbial conditions within sea turtle nesting environments may influence egg health and hatching outcomes, yet information on culturable bacteria associated with sea turtle eggs and nest sediments in Indonesia remains limited. This study characterized culturable bacteria from eggshell surfaces of green turtles (*Chelonia mydas*), olive ridley turtles (*Lepidochelys olivacea*), unhatched eggs, and natural and seminatural hatchery sediments at Batu Hiu Beach, Pangandaran, Indonesia. Three eggs were sampled from each egg category, with eggs within each category originating from the same nest. Bacterial recovery was evaluated using selective and non‐selective media, followed by colony characterization, Gram staining, and 16S rRNA sequencing of 21 representative isolates. Vibrio‐related isolates were screened for toxR, tdh, and trh genes using PCR. Green turtle eggshell swabs showed the highest mean culturable bacterial count (2,358 *C* *F* *U*/*m* *L*), followed by olive ridley eggshell swabs (1,640 *C* *F* *U*/*m* *L*), whereasile hatchery sediment showed the lowest mean count (109 CFU/mL). Bacterial recovery differed among culture media (Friedman *χ*2 = 10.47, *p* = 0.0332; Kendall′’s *W* = 0.436). Molecular analysis showed affiliations with Enterobacteriaceae, Vibrio, and other environmental bacterial groups. All five Vibrio‐related isolates were positive for toxR, whereas tdh and trh were not detected.

## 1. Introduction

Sea turtles are globally categorized as endangered species, facing multiple threats including habitat loss, climate change, and direct exploitation through the illegal trade of their meat and eggs ([1–3] In Indonesia, the consumption of turtle eggs remains widespread and deeply rooted in coastal cultural traditions, despite national and international legal protection. Weak law enforcement and high demand, including cross‐border trade within Southeast Asia, continue to drive exploitation [4, 5]. Among the most frequently consumed are green turtle (*Chelonia mydas*) and olive ridley (*Lepidochelys olivacea*) eggs, valued for their perceived nutritional and cultural significance [6, 7]).

Unlike poultry eggs, turtle eggs lack a protective cuticle layer, making them highly vulnerable to microbial contamination. The porous eggshell structure facilitates bacterial penetration, leading to infections that disrupt embryonic development and reduce hatching success [8, 9]. Microorganisms associated with sea turtle eggs may originate from multiple sources, including maternal‐associated microorganisms, eggshell surfaces, and nesting sediments. These sources contribute to microbial exposure during incubation and may influence microbial conditions surrounding developing eggs [10, 11]. Environmental factors, including nest temperature, moisture, sediment characteristics, and oxygen availability, may further influence microbial colonization and succession during incubation [12, 13]). Changes in microbial composition have been associated with differences in egg conditions and incubation environments, although the direct relationship between microbial composition and hatching outcomes remains complex and requires further investigation [11, 14].

Bacterial communities associated with sea turtle eggs and nesting environments consist of diverse ecological groups. Previous culture‐based and molecular studies have reported the presence of Enterobacteriaceae, including *Salmonella*, *Escherichia*, *Klebsiella*, *Enterobacter*, and *Citrobacter*, from sea turtle eggs, cloacal samples, and nesting substrates [8, 15–17]. These bacterial groups are important because several members possess opportunistic characteristics and may become relevant under favorable environmental conditions. Nevertheless, the direct contribution of these bacteria to embryo mortality or hatching failure remains unclear and requires additional experimental evidence.

In addition to bacterial communities, fungal infections have also been recognized as important microbial threats affecting sea turtle reproductive success. Sea turtle egg fusariosis (STEF) is one of the best documented fungal‐associated diseases affecting turtle eggs and has been linked to embryo mortality and reduced hatching success. Sarmiento‐Ramírez et al. [18] demonstrated that fungi associated with the *Fusarium solani* species complex were involved in infections of sea turtle eggs and represented important fungal agents in nesting environments. Further studies by Sarmiento‐Ramírez et al. [19] expanded this evidence by documenting the distribution of STEF‐associated *Fusarium* pathogens across geographically distinct sea turtle populations. Subsequent studies broadened the understanding of STEF‐associated *Fusarium* beyond sea turtle nesting systems. García‐Martín et al. [20] reported *Fusarium keratoplasticum* in eggs of the freshwater turtle *Podocnemis unifilis*, indicating that STEF‐associated *Fusarium* taxa may also occur in freshwater chelonians. Martínez‐Ríos et al. [21] further reported STEF‐associated *Fusarium* pathogens in turtle‐associated environments, supporting a broader ecological distribution of these fungi.

Although previous studies have substantially improved understanding of fungal‐associated diseases, particularly STEF, bacterial communities associated with sea turtle eggs and nesting sediments remain comparatively less characterized. Information regarding viable culturable bacteria, bacterial abundance, and molecular characteristics of bacterial isolates remains limited. Culture‐based approaches provide complementary information by enabling the recovery of living bacterial isolates that can be further characterized through molecular identification and functional gene screening.

In understanding the ecological and health relevance of marine‐associated bacteria, particular attention has been directed toward the genus *Vibrio*. The genes *tdh* and *trh* are recognized as important virulence‐associated markers in *Vibrio parahaemolyticus*, whereas *toxR* functions as a regulatory gene involved in controlling several virulence‐related pathways [10, 22]. However, detection of *toxR* alone does not confirm pathogenicity because virulence expression depends on additional genetic determinants and environmental regulation. Therefore, interpretation of environmental *Vibrio* isolates requires integration between taxonomic identification and analysis of virulence‐associated markers.

Despite increasing recognition of microbial influences on sea turtle egg health, microbiological investigations of sea turtle eggs in Indonesia remain limited. Previous studies have mainly focused on conservation monitoring, fungal infections, or descriptive microbial observations, whereas integrated assessments combining bacterial enumeration, culture‐based isolation, molecular identification, and screening of functional genetic markers remain scarce.

Therefore, this study is aimed at characterizing culturable bacterial communities associated with green turtle (*Chelonia mydas*) and olive ridley turtle (*Lepidochelys olivacea*) eggs, unhatched eggs, natural nesting sediments, and seminatural hatchery sediments at Batu Hiu Beach, Pangandaran, Indonesia. The study integrates selective and nonselective bacterial cultivation, quantitative bacterial enumeration, colony characterization, 16S rRNA gene‐based identification, and detection of *Vibrio*‐associated genetic markers (*toxR*, *tdh*, and *trh*). Unlike previous studies that have predominantly focused on fungal‐associated egg mortality, this study provides baseline information regarding viable culturable bacterial populations associated with sea turtle eggs and nesting environments in Indonesia. The novelty of this study lies in integrating culture‐based bacterial assessment with molecular identification and targeted screening of *Vibrio*‐associated genetic markers to characterize the bacterial component of sea turtle nesting environments. These findings provide additional microbiological information that complements previous fusariosis‐focused studies and may support future microbial monitoring strategies for sea turtle conservation programs.

## 2. Materials and Methods

### 2.1. Study Location

This study was conducted between September 2024 and March 2025 at Batu Hiu Beach, Pangandaran Regency, West Java, Indonesia. Sampling was carried out during a single nesting season at three purposively selected stations representing different nesting conditions: (1) a seminatural hatchery managed by the Raksa Bintana Foundation (7°41 ^′^32 ^″^ S, 108°32 ^′^9 ^″^ E), (2) a natural green turtle (*Chelonia mydas*) nest (7°41 ^′^21 ^″^S, 108°32 ^′^56 ^″^ E), and (3) a natural olive ridley turtle (*Lepidochelys olivacea*) nest (7°41 ^′^9 ^″^ S, 108°34 ^′^15 ^″^ E) (Figure 1). The purposive sampling approach was applied to capture contrasts between hatchery‐managed and natural nesting environments rather than to infer population‐level bacterial abundance. Due to ethical and conservation constraints, the number of eggs sampled per category was intentionally limited. Consequently, this study was designed as an exploratory assessment aimed at generating baseline microbiological information within the sampled nests.

**Figure 1:**
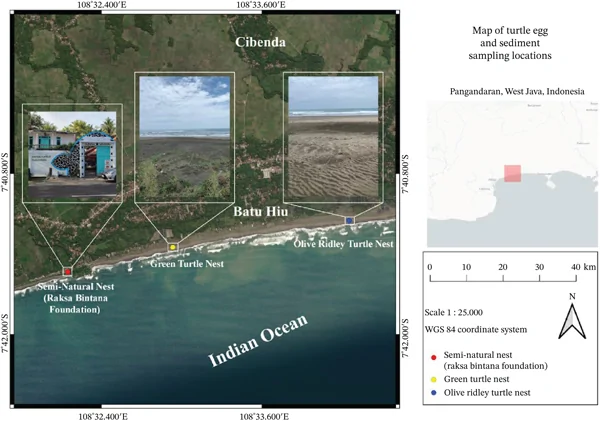
Sampling locations of olive ridley turtle eggs, green turtle eggs, and sediment (source: ArcGIS Pro).

### 2.2. Sample Collection

Eggs and associated nest sediments were collected during the nesting season in September 2024 at Batu Hiu Beach, Pangandaran, Indonesia. A total of three freshly laid olive ridley turtle (*Lepidochelys olivacea*) eggs, three green turtle (*Chelonia mydas*) eggs, and three unhatched eggs were sampled, together with sediments from natural nests and seminatural hatchery nests. The three olive ridley turtle eggs were collected from one natural olive ridley turtle nest, whereas the three green turtle eggs were collected from a separate natural green turtle nest. The three unhatched eggs were obtained from another separate nest. Therefore, eggs within each category originated from the same nest and were treated as within‐nest subsamples rather than independent biological replicates. Natural sediment samples were collected from the corresponding green turtle and olive ridley turtle nesting environments, whereas hatchery sediment samples were collected from the seminatural hatchery area. Samples were included when eggs had intact eggshell surfaces suitable for nondestructive swab sampling and sediments were directly associated with the incubation environment. Eggs were processed individually to characterize within‐nest variation in culturable bacteria associated with eggshell surfaces. Sampling was conducted with permission from the local sea turtle conservation authority at Batu Hiu Beach. The study was also approved by the Research Ethics Committee of Universitas Padjadjaran (Ethical Approval No. 689/UN6.KEP/EC/2025). The number of eggs sampled was limited due to conservation and ethical considerations.

Eggs were collected using sterile gloves and sterile forceps. To standardize sample collection, the eggshell surface was gently cleaned with sterile gauze moistened with physiological saline prior to swabbing. Sterile Amies swabs were then applied to the eggshell surface and sediment samples. The swabbing method was selected as a nondestructive approach for assessing culturable bacteria associated with eggshell surfaces and nest sediments. This approach provided a consistent method for recovering culturable bacteria, particularly aerobic and facultative anaerobic Gram‐negative bacteria [11, 23]. Following collection, swabs were placed in sterile transport tubes, maintained at 4°C–8°C in insulated cool boxes, and transported to the laboratory within 24 h. Blank field controls were included to monitor potential environmental contamination during sampling. All samples were processed aseptically at the Microbiology and Molecular Biotechnology Laboratory, Faculty of Fisheries and Marine Sciences, and the Central Laboratory of Biological Activity, Universitas Padjadjaran.

### 2.3. Bacterial Culture and Enumeration

Bacterial culture was performed using swabs collected from the eggshell surfaces of green turtle (*Chelonia mydas*), olive ridley turtle (*Lepidochelys olivacea*), and associated nest sediments [15]. Cultivation was carried out using a combination of selective and nonselective media, including Salmonella–Shigella agar (SSA; HiMedia, India), eosin‐methylene blue agar (EMBA; HiMedia, India), Mueller–Hinton agar (MHA; HiMedia, India), thiosulfate citrate bile salts sucrose (TCBS) agar (HiMedia, M189‐500G, India), and nutrient agar (NA; Oxoid, CM0003, United Kingdom). Media selection was based on their ability to support bacterial growth and enable presumptive differentiation of specific bacterial groups.

SSA was used for the presumptive isolation of *Salmonella* and *Shigella*, where bile salts and dyes inhibit Gram‐positive bacteria, whereas sodium thiosulfate facilitates the detection of hydrogen sulfide–producing bacteria, typically observed as black‐centered colonies associated with *Salmonella* spp. [24–26]. EMBA functioned as a selective and differential medium for enteric Gram‐negative bacteria. Lactose‐fermenting species such as *Escherichia coli* produced a characteristic metallic green sheen, whereas nonlactose fermenters, including *Salmonella*, appeared colorless [27–29].

MHA was used in this study exclusively as a nonselective medium for preliminary cultivation of culturable bacteria. The use of MHA was based on the limited availability of selective media specific for *Bacillus cereus* in the laboratory, whereas previous studies have shown that *Bacillus cereus* and other *Bacillus* spp. are able to grow well on Mueller–Hinton‐based media, particularly in the context of antimicrobial susceptibility testing [30]. In this study, MHA was not applied as a selective or confirmatory medium for species‐level identification of *Bacillus cereus*, but was restricted to preliminary cultivation and observation of colony characteristics indicative of *Bacillus* spp. Given that growth and colony morphology on MHA are not specific for *Bacillus cereus*, species‐level identification was not inferred, and all isolates recovered on MHA were conservatively reported as *Bacillus*‐like bacteria to avoid overinterpretation of the results [31–33].

TCBS agar was used for the selective isolation of *Vibrio* spp., as its alkaline pH and selective components suppress most nontarget organisms and enhance the recovery of *Vibrio*‐associated bacteria, including *Vibrio parahaemolyticus* [25]. NA served as a general‐purpose, nonselective medium to support the growth of heterotrophic bacteria and for maintaining stock cultures [28].

All inoculated plates were incubated at 37°C for 24–48 h. Colony growth was evaluated based on morphology, pigmentation, and surface characteristics. Presumptive isolates were subsequently subjected to Gram staining to determine Gram reaction and cell morphology [34].

Isolates recovered from SSA, EMBA, and MHA were reported at the presumptive level based on colony morphology and Gram‐stain characteristics, with reference to standard microbiological criteria [34–36]. Conversely, isolates obtained from TCBS and NA were subcultured into nutrient broth and incubated at 37°C for 24 h to obtain pure cultures for subsequent molecular analysis. An incubation temperature of 37°C was used as a laboratory condition to support the recovery of mesophilic bacteria and bacterial groups relevant to the microbiological assessment [37]. This incubation condition may influence the bacterial groups recovered compared with the natural temperature conditions of the nesting environment.

### 2.4. Molecular Analysis of Bacteria Recovered From NA and TCBS Medium

Molecular analysis was conducted on bacterial isolates obtained from TCBS medium (*Vibrio* spp.) and NA (general heterotrophic bacteria). Isolates selected for molecular analysis were based on distinct colony morphology, particularly colony color and shape, observed after culture and purification. Representative isolates with distinct morphological characteristics were subsequently selected for molecular identification. Molecular identification followed the method of Delidow et al. [38] with several modifications. DNA was extracted using the Wizard Genomic DNA Purification Kit A1120 (Promega).

PCR amplification targeted the universal 16S rRNA gene and the gyrB gene for higher resolution species‐level identification of *Vibrio* species. Two primer sets were used: 27F (AGAGTTTGATCCTGGCTCAG) and 1492R (TACGGYTACCTTGTTACGACTT) for the 16S rRNA gene (1460 bp; [39]) and VF1 (AARCARGGNCGTAACCGTAA) and VR1 (HGGGTADCGRCGRCTCAT) for the gyrB gene (510 bp; [40]). The PCR mixture (35 *μ*L) consisted of 17.5 *μ*L Green Taq Master Mix 2×, 11.9 *μ*L nuclease‐free water, 1.4 *μ*L forward primer, 1.4 *μ*L reverse primer, and 2.8 *μ*L DNA template.

The PCR conditions were as follows:

• 16S rRNA: initial denaturation at 94°C for 90 s; 30 cycles of denaturation at 95°C for 30 s, annealing at 48.5°C for 30 s, extension at 72°C for 30 s; final extension at 72°C for 5 min.
• gyrB: initial denaturation at 94°C for 4 min; 35 cycles of denaturation at 94°C for 1 min, annealing at 60°C for 1 min, extension at 72°C for 1 min; final extension at 72°C for 10 min; storage at 4°C [40].

PCR products were separated on 1.5% agarose gels (Mupid‐exU, Japan) and visualized with a UV transilluminator (UVITEC Cambridge). Positive amplicons were subsequently sequenced (1st BASE DNA Sequencing). Sequence data were analyzed using BioEdit and MEGA11 [41] and compared with GenBank references using BLAST. Taxonomic assignments were based on sequence similarity, alignment quality, query coverage, and comparison with available reference sequences in the NCBI GenBank database [42–46]. Species‐level identification was assigned when sequence similarity and taxonomic resolution were sufficient. Isolates with insufficient resolution were reported at the genus level or as the closest related taxon rather than as confirmed species [42, 44, 46, 47]. Phylogenetic trees were constructed using the neighbor‐joining method with 1000 bootstrap replicates. Isolates showing < 98.65% similarity to reference strains were considered potential novel species [40].

The 16S rRNA gene is widely used to identify microbial communities associated with turtle eggs [48], whereas the gyrB gene provides higher taxonomic resolution for differentiating closely related *Vibrio* species [49, 50]. Molecular analyses have also revealed that fungal pathogens such as *Fusarium* spp. are associated with hatching failures [51, 52]. Environmental factors such as nest moisture, temperature, and maternal effects strongly influence microbial communities and embryo survival [53].

### 2.5. Detection of Toxin Genes of *Vibrio* spp.

Detection of toxin genes in *Vibrio* spp. isolates was carried out using specific primers targeting the *toxR*, *tdh*, and *trh* genes (Table 1). PCR reactions were prepared in a final volume of 50 *μ*L, consisting of 50 ng DNA template, 10 pmol of each primer, 25 *μ*L of 2× PCR Master Mix (containing 2.4 mM dNTP and 0.3 units of Taq DNA polymerase; Promega, United States), and sterile distilled water.

**Table 1:** Primer sequences used for detection of toxin genes in *Vibrio* spp.

Gene	Primer name	Nucleotide sequence (5 ^′^ → 3 ^′^)	Product size (bp)	References
toxR	toxR‐F	GTCTTCTGACGCAATCGTTG	367	[54, 55]
	toxR‐R	ATACGAGTGGTTGCTGTCATG	367	[54, 55]
tdh	tdh‐F	GTAAAGGTCTCTGACTTTTGGAC	500	[54, 55]
	tdh‐R	TGGAATAGAACCTTCATCTTCACC	500	[54, 55]
trh	trh‐F	TTGGCTTCGATATTTTCAGTATCT	269	[54, 55]
	trh‐R	CATAACAAACATATGCCCATTTCC	269	[54, 55]

*Note:* References: Luan et al., 2007; Marlina et al., 2007 [54, 55].

Abbreviations: *tdh*, thermostable direct hemolysin; *tlh*, thermolabile hemolysin; *toxR*, toxin operon regulator; *trh*, TDH‐related hemolysin.

Amplification was performed in an MJ Mini Thermal Cycler (Bio‐Rad, United States) with the following program: initial denaturation at 94°C for 3 min, followed by 30 cycles of denaturation at 94°C for 1 min, annealing at 50°C for 1 min, and extension at 72°C for 1 min. A final extension was performed at 72°C for 7 min.

PCR products were separated by electrophoresis on 1% agarose gels using 1× TAE buffer, stained with ethidium bromide, and visualized with a UV transilluminator system (DyNa Light).

### 2.6. Enumeration of Culturable Bacteria on Selective and Nonselective Media

Total plate count (TPC) was applied to estimate culturable bacterial counts recovered from eggshell surface swabs, unhatched egg swabs, and associated nest sediment swabs from natural and hatchery environments. Bacterial enumeration was performed using both selective and nonselective media, including SSA for presumptive *Salmonella*/*Shigella*‐like colonies, EMBA (Levine) for presumptive coliform or *E. coli*–like colonies, MHA for *Bacillus*‐like bacteria, TCBS agar for presumptive *Vibrio* spp., and NA as a general medium for total heterotrophic bacteria.

After incubation, colonies were counted on plates containing 30–300 colonies. Bacterial counts were expressed as colony‐forming units per milliliter of swab suspension using the following equation [56]:

(1) Bacteria count CFU mL−1=no.of colonies×dilution factorvolume plated mL,

where CFU denotes colony‐forming units.

Because bacteria were recovered from surface swabs eluted in sterile saline rather than from homogenized egg contents, all enumeration results were expressed as colony‐forming units per milliliter of swab suspension. Thus, the results describe culturable bacteria associated with the sample surfaces.

### 2.7. Statistical Analysis

Culturable bacterial counts were analyzed using descriptive and exploratory nonparametric approaches because eggs within each category originated from the same nest and were not considered independent biological replicates. Data were summarized using the mean, standard deviation, median, minimum, and maximum values for each sample category and culture medium. Overall differences among the five culture media were evaluated using the Friedman test, with sample categories treated as blocks and culture media as repeated recovery conditions [57–59]. Kendall′s *W* was calculated as the effect size. When the Friedman test was significant, paired Wilcoxon signed‐rank tests with Holm correction were used for exploratory pairwise comparisons among media [60, 61]. Because the number of independent nests was limited, differences among turtle species, egg categories, and nesting environments were interpreted descriptively rather than as population‐level inference. A log_10_ (CFU/mL + 1) transformation was used only for visualization. Statistical significance was set at *α* = 0.05, and all analyses were performed using R software.

## 3. Result

### 3.1. Bacterial Counts Across Samples

Culturable bacterial recovery from sea turtle eggshell swabs and associated nest sediments was evaluated using selective and nonselective culture media (Figure 2). The analyzed samples included green turtle eggshell swabs, olive ridley turtle eggshell swabs, unhatched egg swabs, natural nest sediments associated with each turtle species, and seminatural hatchery sediment. Bacterial counts were expressed as colony‐forming units per milliliter of swab suspension.

**Figure 2:**
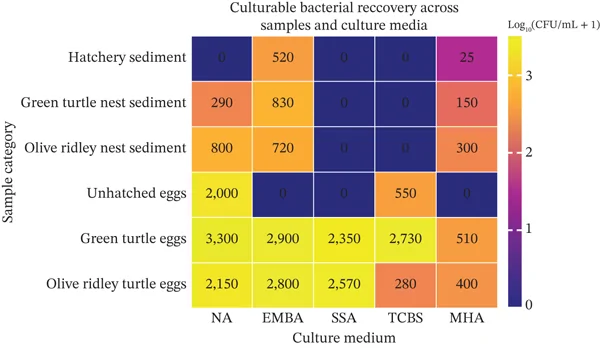
Culturable bacterial counts recovered from sea turtle eggshell swabs and nest sediment samples across five culture media. Cell values indicate bacterial counts in colony‐forming units per milliliter, whereas the color gradient represents log_10_ (CFU/mL + 1). The heatmap presents descriptive patterns of bacterial recovery under the applied culture conditions.

Descriptively, the highest mean bacterial recovery was observed on NA (1423.3 CFU/mL), followed by EMBA (1295.0 CFU/mL), SSA (820.0 CFU/mL), TCBS (593.3 CFU/mL), and MHA (230.8 CFU/mL) (Table 2). The relatively large standard deviations observed for several media reflected substantial variation in bacterial recovery among the sampled eggshell and sediment categories.

**Table 2:** Descriptive and exploratory statistical analysis of culturable bacterial recovery across culture media.

Medium	Mean (CFU/mL)	SD	Median	Min–Max
NA	1423.3	1271.0	1400	0–3300
EMBA	1295.0	1238.0	775	0–2900
SSA	820.0	1272.0	0	0–2570
TCBS	593.3	1070.0	140	0–2730
MHA	230.8	207.0	225	0–510

At the sample level, green turtle eggshell swabs showed the highest mean recovery across the five culture media (2358 CFU/mL), followed by olive ridley turtle eggshell swabs (1640 CFU/mL). Lower mean values were observed for unhatched eggs (510 CFU/mL), olive ridley nest sediment (364 CFU/mL), green turtle nest sediment (254 CFU/mL), and hatchery sediment (109 CFU/mL). These values represent descriptive patterns within the sampled nests and should not be interpreted as population‐level differences among turtle species or nesting environments.

### 3.2. Culturable Bacterial Recovery Across Culture Media

Differences in bacterial recovery among the five culture media were further examined using an exploratory Friedman test. Sample categories were treated as blocks, whereas culture media represented repeated laboratory recovery conditions. This analysis was used to evaluate overall variation in culture‐dependent bacterial recovery among media and was not interpreted as a population‐level comparison among turtle species or nesting environments (Figure 3).

**Figure 3:**
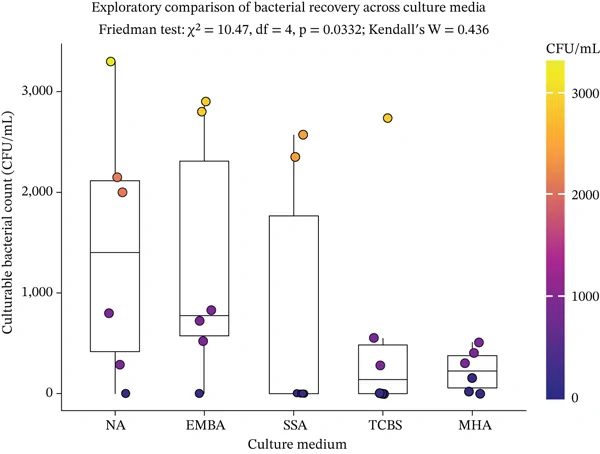
Culturable bacterial counts across culture media. The Friedman test indicated an overall difference among media.

The Friedman test indicated an overall difference in culturable bacterial recovery among culture media (*χ* ^2^ = 10.47, df = 4, *p* = 0.0332). Kendall′s coefficient of concordance was 0.436, indicating a moderate effect size. However, pairwise Wilcoxon signed‐rank tests with Holm correction did not identify significant differences between individual pairs of culture media (all adjusted *p* > 0.05). Although NA and EMBA showed the highest mean recovery, followed by SSA, TCBS, and MHA, the absence of significant pairwise differences indicates that no individual medium could be statistically distinguished from another after correction for multiple comparisons. The significant overall Friedman result therefore reflects variation in the collective recovery pattern across the five media rather than a statistically supported difference between specific pairs of media.

### 3.3. Morphological Characteristics of Bacteria

Observations on selective and nonselective media revealed clear differences in colony appearance and Gram staining (Table 3). On NA, colonies were the most diverse, with variations in color (orange, white, and yellow) and cell shape (cocci, bacilli, and spiral), reflecting the broad heterotrophic community in turtle eggs and sediments.

**Table 3:** Gram staining and morphological characteristics of bacteria isolated from selective and nonselective media.

Media	Presumptive/isolated bacteria	Gram result	Morphology	References/source
NA	Total heterotrophic bacteria	±	Cocci, bacilli, spiral; colonies orange/white/yellow, pink, black	This study
TCBS	*Vibrio* spp.	−	Curved rods, cocci; colonies yellow/green/orange	This study
EMBA	*Escherichia coli*	−	Bacilli; nonspore; lactose fermenter, metallic sheen	[34]; [36]; [62]
SSA	*Salmonella* spp.	−	Bacilli; nonspore; H_2_S producer, black colonies	[34]; [36]; [62]
MHA	*Bacillus* spp.	+	Large bacilli; spore‐forming; catalase +	[35]; [63]; [64]

On TCBS, *Vibrio* spp. appeared as yellow, green, or orange colonies, consistent with sucrose‐fermenting and nonfermenting strains, and were confirmed as Gram‐negative curved rods (comma‐shaped). On EMBA, *E. coli* produced the characteristic metallic green sheen and was identified as Gram‐negative bacilli and lactose fermenting. On SSA, *Salmonella* spp. formed black‐centered colonies due to H_2_S production and were identified as Gram‐negative bacilli, nonspore forming, and nonlactose fermenting. On MHA, isolates were identified as *Bacillus* spp., forming large opaque colonies. Gram staining showed Gram‐positive bacilli in chains with endospores and catalase‐positive activity. Overall, colony morphology and Gram staining confirmed the presence of key pathogens (*Vibrio* spp., *E. coli*, *Salmonella* spp., and *Bacillus* spp.) on selective media, whereas NA highlighted the wider diversity of heterotrophic bacteria.

### 3.4. Molecular Identification of Culturable Bacterial Isolates Recovered From NA and TCBS Medium

Sequencing of the 16S rRNA gene was performed to identify representative culturable bacterial isolates recovered from NA and TCBS media (Figure 4, Table 4). A total of 21 isolates were successfully identified, representing enteric bacteria, marine‐associated taxa, and environmental bacteria cultivated under the applied culture conditions.

**Figure 4:**
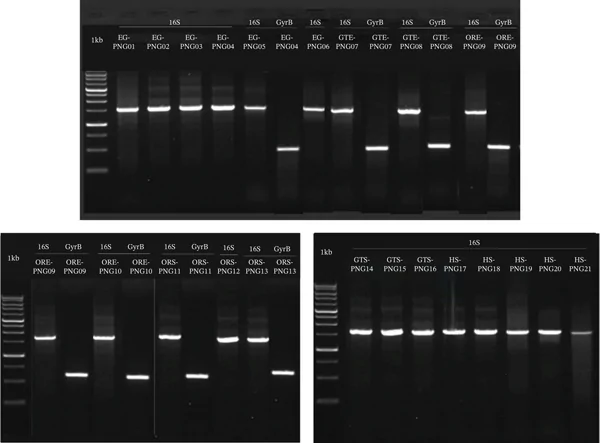
Agarose gel electrophoresis of 16S rRNA and *gyrB* gene amplification from bacterial isolates.

**Table 4:** Closest taxonomic matches of culturable bacterial isolates recovered from NA and TCBS media based on 16S rRNA gene sequence comparison with NCBI GenBank references.

No.	Code	Closest BLAST match (GenBank reference)	GenBank accession no.	*E* value	16S rRNA sequence similarity (%)	Taxonomic assignment	No. of isolates	Isolated from	Medium	Colony color and shape
1	EG‐PNG01	*Klebsiella* sp. clone	KT294091.1	3.0 × 10^−51^	83.40	*Klebsiella*‐related isolate	1	Unhatched egg	NA	White (bacilli, spiral)
2	EG‐PNG02	*Klebsiella pneumoniae* strain EMB.W.KL.GH1.C3	MK719834.1	0.0	94.29	*Klebsiella* sp.	1	Unhatched egg	NA	Orange (bacilli, spiral)
3	EG‐PNG03	*Enterobacter asburiae* strain RCB707	KT260919.1	7.0 × 10^−92^	76.38	*Enterobacter*‐related isolate	1	Unhatched egg	NA	Yellow (bacilli, spiral)
4	EG‐PNG04	*Citrobacter* sp. strain CPO 2.210	OR426156.1	0.0	91.19	*Citrobacter* sp.	1	Unhatched egg	TCBS	Green (comma, coccus)
5	EG‐PNG05	*Shewanella* sp. A2 MF‐2014	LK392351.1	1.0 × 10^−53^	79.34	*Shewanella*‐related isolate	1	Unhatched egg	TCBS	White flower‐like (comma, coccus)
6	EG‐PNG06	*Providencia rettgeri* strain CR	PQ496001.1	8.0 × 10^−74^	81.95	*Providencia*‐related isolate	1	Unhatched egg	TCBS	Yellow flower‐like (comma, coccus)
7	GTE‐PNG07	*Vibrio parahaemolyticus* strain HSEMS	PP116102.1	0.0	95.06	*Vibrio* sp. closely related to *V. parahaemolyticus*	1	Green turtle egg	TCBS	Orange (coccus, comma)
8	GTE‐PNG08	*Vibrio parahaemolyticus* strain CSBG	PP106503.1	0.0	95.04	*Vibrio* sp. closely related to *V. parahaemolyticus*	1	Green turtle egg	TCBS	Green (comma, coccus)
9	ORE‐PNG09	*Vibrio alginolyticus* strain AA62	MW255275.1	0.0	94.14	*Vibrio* sp. closely related to *V. alginolyticus*	1	Olive ridley turtle egg	TCBS	Green (comma, coccus)
10	ORE‐PNG10	*Vibrio alginolyticus* strain NBRC 15630	PQ443934.1	0.0	90.20	*Vibrio*‐related isolate	1	Olive ridley turtle egg	TCBS	Dark green (comma, coccus)
11	ORS‐PNG11	*Vibrio alginolyticus* strain CICC 10889	OR965054.1	0.0	92.00	*Vibrio* sp.	1	Olive ridley turtle sediment	NA	White (bacilli)
12	ORS‐PNG12	*Marinobacter* sp.	OQ570258.1	3.0 × 10^−126^	79.88	*Marinobacter*‐related isolate	1	Olive ridley turtle sediment	NA	Yellow (bacilli, spiral)
13	ORS‐PNG13	*Sutcliffiella halmapala*	KU955629.1	0.0	85.40	*Sutcliffiella*‐related isolate	1	Olive ridley turtle sediment	NA	Orange (bacilli, spiral)
14	GTS‐PNG14	*Pseudomonas fragi* strain J5	PP725227.1	0.0	88.78	*Pseudomonas* sp.	1	Green turtle sediment	NA	White (spiral, bacilli)
15	GTS‐PNG15	Bacterium clone GBO51O2hO6	JN672574.1	0.0	95.31	Unclassified bacterium‐related isolate	1	Green turtle sediment	NA	Yellow (spiral, bacilli)
16	GTS‐PNG16	*Bacillus* sp. (Firmicutes) strain CJKOP‐136	MF537181.1	0.0	89.54	*Bacillus*‐related isolate	1	Green turtle sediment	NA	Orange (spiral, bacilli)
17	HS‐PNG17	*Salmonella enterica* subsp. *enterica* serovar Paratyphi B strain CFSAN008708	CP074668.1	5.0 × 10^−94^	76.42	*Salmonella*‐related isolate	1	Hatchery sediment	NA	Black (spiral, bacilli)
18	HS‐PNG18	Bacterium clone GBO51O2hO	JN672574.1	0.0	96.58	Unclassified bacterium‐related isolate	1	Hatchery sediment	NA	Pink (spiral, bacilli)
19	HS‐PNG19	*Pseudomonas corrugata* strain p17_D02	JQ833503.1	2.0 × 10^−42^	77.50	*Pseudomonas*‐related isolate	1	Hatchery sediment	TCBS	Yellow (coccus)
20	HS‐PNG20	*Aeromonas* sp. strain CQ11 16S	OL477328.1	0.0	94.11	*Aeromonas* sp.	1	Hatchery sediment	TCBS	Orange (coccus)
21	HS‐PNG21	*Citrobacter* sp.	HQ108124.1	0.0	99.77	*Citrobacter* sp.	1	Hatchery sediment	TCBS	Dark green (comma)

Figure 4 presents agarose gel electrophoresis images documenting successful PCR amplification of the 16S rRNA and gyrB genes prior to sequencing. Gel images are provided solely as technical verification of amplification quality and were not used for quantitative or ecological interpretation.

Unhatched eggs were associated with culturable bacterial isolates showing closest sequence matches to members of the Enterobacteriaceae, including *Klebsiella*‐related, *Enterobacter*‐related, *Citrobacter-*related, and *Providencia-*related taxa. In contrast, eggs from green and olive ridley turtles yielded isolates with closest relationships to *Vibrio* reference sequences, including taxa related to *Vibrio parahaemolyticus* and *Vibrio alginolyticus*. Sediment samples exhibited broader taxonomic affiliations and included isolates related to *Marinobacter*, *Sutcliffiella*, *Pseudomonas*, *Bacillus*, *Aeromonas*, *Salmonella*, and *Citrobacter*.

Sequence similarity values varied among isolates and influenced the level of taxonomic assignment. Isolates were assigned at the genus level when supported by sufficient sequence resolution, whereas isolates with lower similarity values were reported as closely related taxa rather than confirmed species. Low sequence similarity values were not interpreted as evidence of novel species or strains because additional genomic characterization would be required. These results describe the taxonomic affiliations of culturable bacterial isolates recovered under selective and nonselective culture conditions and do not represent the complete bacterial community composition, microbiome structure, or relative abundance within turtle eggs or sediments.

Phylogenetic analysis based on 16S rRNA gene sequences revealed clustering patterns of isolates according to their taxonomic affiliations rather than their sampling sources (Figure 5). Isolates assigned to the genus *Vibrio* formed a distinct cluster within the *Vibrio*‐associated lineage, with closely related isolates showing bootstrap support values ranging from 81% to 87%. The clustering pattern indicated genetic similarity among *Vibrio*‐related isolates obtained from different turtle egg and sediment samples; however, species‐level identification was not inferred solely from 16S rRNA sequences.

**Figure 5:**
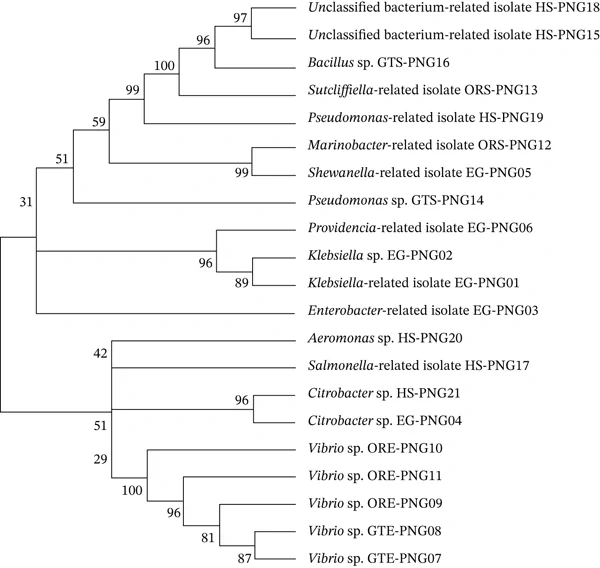
Phylogenetic tree based on 16S rRNA gene sequences of culturable bacterial isolates recovered from sea turtle eggs and surrounding sediments in Pangandaran, Indonesia. The evolutionary history was inferred using the neighbor‐joining method, with bootstrap values calculated from 1000 replicates and displayed next to the branches.

Members of the Enterobacteriaceae‐related isolates formed closely related clusters distinct from marine‐associated bacterial groups, including *Vibrio*‐related isolates and *Marinobacter*‐related isolates. In contrast, isolates assigned to *Bacillus*‐ and *Sutcliffiella*‐related groups formed separate clades consistent with their broader taxonomic relationships. These phylogenetic patterns indicate that genetic relatedness among isolates was primarily associated with taxonomic affiliation rather than ecological origin or sampling environment.

### 3.5. Detection of *Vibrio* Virulence Genes

Figure 6 presents the agarose gel electrophoresis results of PCR amplification targeting toxR, tdh, and trh genes from presumptive *Vibrio* isolates recovered from TCBS medium. Clear amplification bands corresponding to the toxR gene were detected in all tested isolates, including isolates showing close relationships with *Vibrio parahaemolyticus* and *Vibrio alginolyticus* reference sequences based on 16S rRNA analysis. In contrast, no amplification products were observed for the hemolysin‐associated genes tdh and trh in any of the tested isolates.

**Figure 6:**
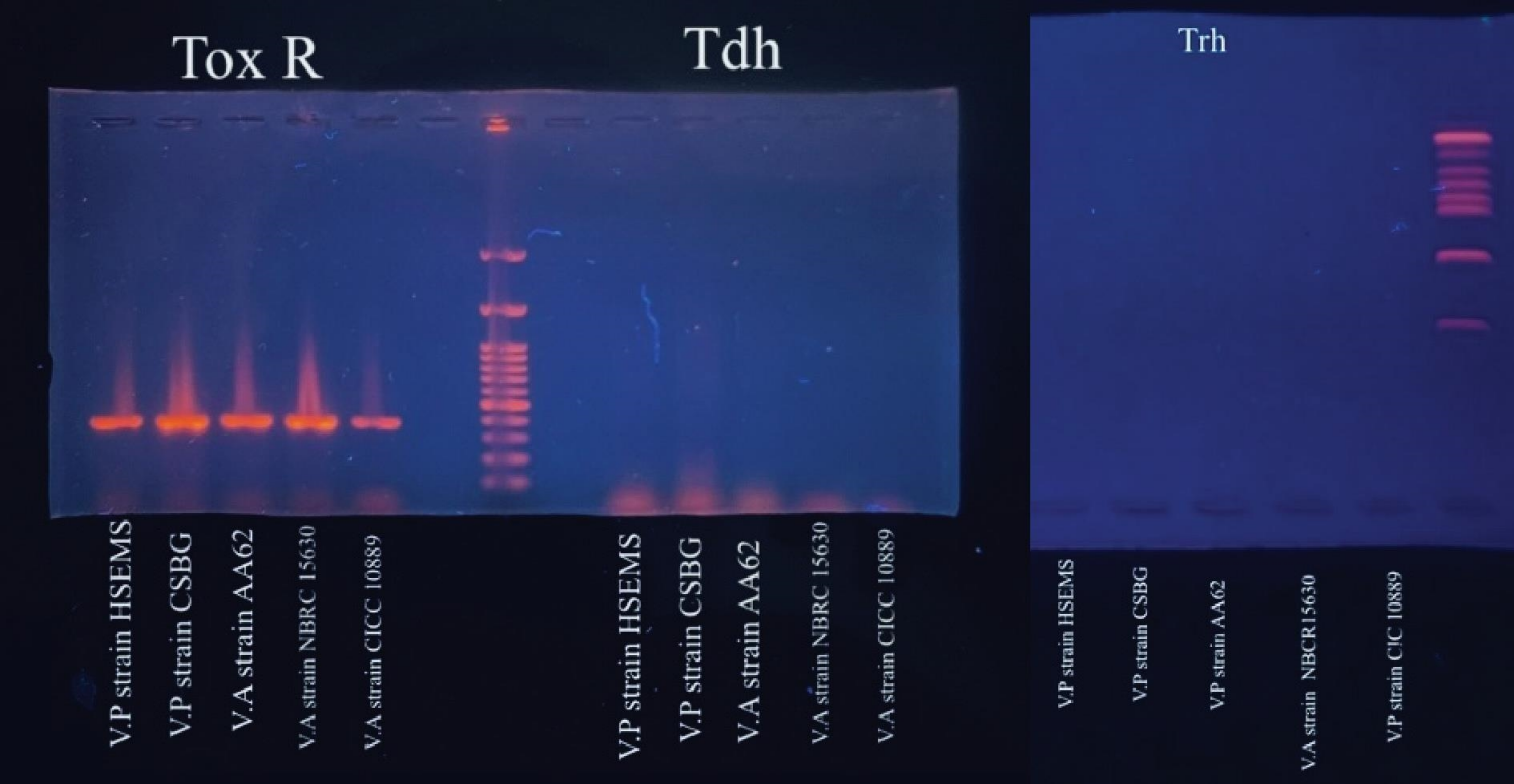
Agarose gel electrophoresis of PCR products targeting *Vibrio* virulence genes (*toxR*, *tdh*, and *trh*).

The detection of toxR indicates the presence of a genetic marker associated with the *Vibrio* group, whereas the absence of tdh and trh indicates that these specific virulence‐associated genes were not detected among the analyzed isolates. The results demonstrate variation in the distribution of *Vibrio*‐associated genetic markers among culturable isolates recovered from sea turtle eggs and nest sediments.

Table 5 summarizes the detection of *Vibrio*‐associated and virulence‐related genes among culturable isolates recovered from turtle eggs and sediments. All tested isolates showed positive amplification of the *toxR* marker, whereas amplification of *tdh* and *trh* was not detected. These findings indicate that the analyzed isolates contained a *Vibrio*‐associated genetic marker but lacked the specific hemolysin‐associated genes evaluated in this study. The presence of *toxR* among the isolates was observed across different sampling sources, including turtle eggs and sediment samples. However, the detection pattern of *toxR*, *tdh*, and *trh* represents only the genetic characteristics of the analyzed isolates under the applied molecular conditions and does not represent the complete virulence profile of the bacterial strains.

**Table 5:** Detection of *Vibrio*‐associated and virulence‐related genes (*toxR*, *tdh*, and *trh*) in culturable *Vibrio* isolates recovered from sea turtle eggs and sediments.

No.	Isolate code	Taxonomic assignment based on 16S rRNA	*toxR*	*tdh*	*trh*	Interpretation
1	VP strain HSEMS	*Vibrio* sp. closely related to *V. parahaemolyticus*	+	−	−	*toxR* detected; *tdh* and *trh* not detected
2	VP strain CSBG	*Vibrio* sp. closely related to *V. parahaemolyticus*	+	−	−	*toxR* detected; *tdh* and *trh* not detected
3	VA strain AA62	*Vibrio* sp. closely related to *V. alginolyticus*	+	−	−	*toxR* detected; *tdh* and *trh* not detected
4	VA strain NBRC15630	*Vibrio*‐related isolate	+	−	−	*toxR* detected; *tdh* and *trh* not detected
5	VA strain CICC10889	*Vibrio* sp.	+	−	−	*toxR* detected; *tdh* and *trh* not detected

## 4. Discussion

This study showed that culturable bacterial counts recovered from eggshell swabs of green turtle (*Chelonia mydas*) and olive ridley turtle (*Lepidochelys olivacea*) eggs were higher than those recovered from natural and hatchery nest sediment samples. However, because eggs within each category originated from the same nest, these findings should be interpreted as exploratory observations describing within‐nest bacterial recovery patterns rather than population‐level differences. The limited number of independent nests and conservation restrictions during sampling were considered when interpreting bacterial abundance patterns.

Quantitative enumeration results summarized in Table 2 showed that eggshell swab samples generally yielded higher recoverable bacterial counts compared with associated sediment samples across the applied culture media. Fresh eggshell samples showed higher bacterial recovery compared with unhatched eggs and sediment samples. This pattern was also illustrated in Figure 2, where eggshell‐associated samples showed greater variability compared with sediment samples. These results suggest that eggshell surfaces may provide a distinct microenvironment that supports bacterial attachment and recovery during incubation. However, culture‐based enumeration represents only the fraction of bacteria capable of growing under the applied laboratory conditions and does not represent the complete bacterial composition associated with eggshell surfaces or nesting sediments.

The higher recovery of culturable bacteria from eggshell surfaces may be related to the structural characteristics of sea turtle eggs and their interaction with surrounding environments. Unlike poultry eggs, sea turtle eggs lack a protective cuticle layer, and their porous eggshell structure may facilitate bacterial attachment from sand, seawater, and maternal‐associated sources [8, 9]. Previous studies have demonstrated that microorganisms associated with sea turtle eggs originate from multiple sources, including eggshell‐associated microorganisms and nesting sediments [10, 11]. Environmental factors, including nest temperature, moisture, and sediment characteristics, may further influence microbial colonization patterns during incubation [52, 65]. Bézy et al. [66] reported that microbial abundance in olive ridley turtle nests was associated with hatching outcomes, whereas environmental conditions may modify microbial dynamics within nesting environments [11, 67]. Nevertheless, the recovery of culturable bacteria from eggshell surfaces does not directly indicate infection, embryo mortality, or reduced hatching success.

Beyond microbial processes, environmental stability remains an important factor influencing sea turtle reproductive success. Climate change may affect nesting habitats through shoreline loss, altered sediment characteristics, and increased flooding frequency. Rising sea levels can reduce suitable nesting areas, whereas prolonged flooding has been associated with increased egg mortality [68–70]. These environmental changes may influence nest conditions, including moisture and temperature, which can subsequently affect microbial dynamics. Therefore, conservation strategies should integrate habitat management, appropriate hatchery practices, and microbial monitoring approaches [71–73].

Culture‐based isolation followed by molecular identification revealed several bacterial groups associated with eggshell surfaces and nesting sediments (Table 4). Based on colony morphology and culture characteristics, several isolates were initially classified as presumptive enteric‐associated bacteria. However, these preliminary classifications require cautious interpretation because colony morphology and biochemical characteristics alone cannot confirm bacterial species identity. Molecular identification based on 16S rRNA gene sequencing provided additional taxonomic resolution for selected isolates. Isolates with sufficient sequence similarity were assigned to the corresponding taxonomic level, whereas isolates with lower similarity were interpreted as closely related taxa rather than definitive species identification.

The recovered isolates represented several bacterial groups, including Enterobacteriaceae‐associated bacteria, marine‐associated bacteria, and environmental bacterial taxa (Table 4). Enterobacteriaceae‐related isolates showed similarity with members of *Klebsiella*, *Enterobacter*, *Citrobacter*, and *Salmonella*‐associated groups. These bacterial groups have previously been reported from sea turtle eggs and nesting environments ([15]). Their occurrence may reflect environmental inputs from sand, seawater, or animal‐associated sources. Similar bacterial groups, including *Bacillus*, *Salmonella*, and *Citrobacter*, have also been reported in turtle‐associated samples [15, 74]. However, the detection of these bacterial groups on eggshell surfaces should not be interpreted as direct evidence of pathogenicity, embryo infection, or zoonotic transmission because this study evaluated only culturable bacteria recovered from external eggshell surfaces.

The phylogenetic relationships of selected bacterial isolates based on 16S rRNA gene sequences are presented in Figure 5. The isolates clustered mainly according to their taxonomic relationships rather than their sampling origin. Enterobacteriaceae‐associated isolates grouped with related reference sequences, whereas marine‐associated bacteria formed separate clusters. Several isolates showed lower similarity values compared with available GenBank references, indicating limitations in database representation and the need for additional molecular markers for more accurate taxonomic resolution [40].

Marine‐associated isolates related to *Vibrio* were also recovered from eggshell and sediment samples (Table 4). Several isolates showed similarity with *Vibrio parahaemolyticus*–associated and *Vibrio alginolyticus*–associated sequences. These bacterial groups are commonly distributed in coastal environments and may occur naturally in marine ecosystems [75–77]. However, these findings should be interpreted as the detection of *Vibrio*‐associated environmental bacteria rather than confirmation of pathogenic strains.

Virulence‐associated gene screening of *Vibrio*‐associated isolates is summarized in Table 5 and Figure 6. All tested isolates showed amplification of the regulatory gene *toxR*, whereas the hemolysin genes *tdh* and *trh* were not detected. Previous studies have shown that environmental *Vibrio* isolates may contain regulatory genes while lacking major toxin‐associated genes [78, 79]. The presence of *toxR* alone does not confirm pathogenicity because virulence expression depends on multiple genetic determinants and environmental regulation. Therefore, the detected *Vibrio*‐associated isolates should be interpreted as *toxR*‐positive environmental isolates rather than confirmed pathogenic bacteria. Tropical aquatic environments are recognized as reservoirs of diverse *Vibrio* populations, where genetic exchange may contribute to variation among environmental strains [80, 81].

Taken together, the findings indicate that sea turtle eggshell surfaces and associated nest sediments contain diverse culturable bacterial isolates, including presumptive enteric‐associated bacteria and molecularly characterized *Vibrio*‐associated isolates. The integration of bacterial enumeration (Table 2), molecular identification (Table 4; Figure 5), and *Vibrio*‐associated genetic screening (Table 5; Figure 6) provides baseline information regarding bacterial components of sea turtle nesting environments. Rather than defining turtle eggs as bacterial hotspots, pathogenic reservoirs, or direct zoonotic sources, this study provides preliminary microbiological evidence from a limited number of samples and highlights the importance of continued microbial monitoring within sea turtle conservation programs.

### 4.1. Limitations and Future Directions

This study has several limitations. The sample size was relatively small, and sampling was conducted during a single nesting season at one study site. In addition, samples were obtained using eggshell and sediment swabs, such that the results represent culturable bacteria associated with sample surfaces. Furthermore, isolates recovered from SSA, EMBA, and MHA media were identified based on colony morphology and Gram staining, whereas molecular confirmation was performed only for selected isolates recovered from NA and TCBS media. Future studies should include larger sample sizes, multiple nests, multiseasonal monitoring, and biochemical or molecular confirmation of presumptive isolates.

## 5. Conclusion

This study provides baseline information on culturable bacteria recovered from sea turtle eggshell surfaces and associated nesting sediments at Batu Hiu Beach, Pangandaran. Isolates with distinct colony morphology were further analyzed using 16S rRNA and, for selected *Vibrio*‐associated isolates, gyrB analysis. Molecular analysis enabled several isolates to be identified to the species level, whereas isolates with insufficient sequence resolution were reported at the genus level or as the closest related taxon. All tested *Vibrio* isolates carried the *toxR* gene, whereas *tdh* and *trh* were not detected. The presence of *toxR* was interpreted as a genetic characteristic of the tested isolates and not as evidence of confirmed virulence or pathogenicity. Given the limited number of eggs and nests, the single study location, and the focus on the culturable bacterial fraction, the findings are exploratory and should not be generalized to the broader sea turtle population or the complete bacterial community. These findings provide baseline information to support further research and microbiological monitoring of sea turtle nesting and seminatural hatchery environments at Batu Hiu Beach.

## Author Contributions

**Titin Herawati**: conceptualization, writing—original draft preparation, writing—review and editing, methodology, funding, investigation, resources, and supervision; **Indriyani Rahayu**: writing—original draft preparation, writing—review and editing, methodology, data curation, formal analysis, resources, software, validation, and visualization; **Aisyah Aisyah**: data curation, formal analysis, methodology, and investigation; **Mochamad Untung Kurnia Agung**: methodology, data curation, investigation, reviewing, and editing; **Donny Juliandri Prihadi**: reviewing and editing; **Mochamad Rudyansyah Ismail**: reviewing and editing; **Eri Bachtiar**: reviewing and editing; **Adiana Ghazali**: reviewing and editing; **Yudi Nurul Ihsan**: reviewing and editing.

## Funding

This work was supported by Universitas Padjadjaran (10.13039/501100015690, 2200/UN6.M/PT.01.03/2025).

## Ethics Statement

This study was approved by the Research Ethics Committee of Universitas Padjadjaran, Indonesia (Approval No. 689/UN6.KEP/EC/2025).

## Conflicts of Interest

The authors declare no conflicts of interest.

## Supporting Information

Additional supporting information can be found online in the Supporting Information section.

## Supporting information


**Supporting Information 1** File S1: Representative 16S rRNA gene sequence files of bacterial isolates used for molecular identification.

**Supporting Information 2** File S2: Representative gyrB gene sequence files of selected bacterial isolates.

**Supporting Information 3** File S3: NCBI BLAST search results and supporting molecular identification outputs for bacterial isolates.

**Supporting Information 4** File S4: Raw bacterial count data, Gram staining results, and statistical outputs used in this study.

## Data Availability

The data that support the findings of this study are available in the supporting information of this article.
