# Supplementary material for: Molecular Identification of Culturable Bacteria and Vibrio Virulence‐Related Genes Associated With Sea Turtle Eggshells and Nest Sediments in Pangandaran, Indonesia
Source: Int J Microbiol. 2026 Oct 5;2026:7422673. doi: 10.1155/ijm/7422673 (PMC13639395; doi:10.1155/ijm/7422673)
Supplement: Supplementary file 2 [file IJM-2026-7422673-s002.zip › Hasil NCBI/US Consensus.docx]

>CP074668.1 Salmonella enterica subsp. enterica serovar Paratyphi B strain CFSAN008708 chromosome, complete genome

GTGTCACTTTCGCTTTGGCAGCAGTGTCTTGCCCGATTGCAGGATGAGTTACCAGCCACAGAATTCAGTA

TGTGGATACGCCCGTTGCAGGCGGAACTGAGCGATAACACGCTGGCTTTGTATGCGCCAAACCGTTTTGT

GCTCGATTGGGTAAGAGATAAGTACCTCAACAATATCAATGGATTACTCAACACATTCTGCGGCGCGGAT

GCCCCACAACTGCGTTTTGAAGTGGGAACAAAGCCCGTTACGCAAACGCTAAAAACGCCTGTGCATAACG

TTGTCGCGCCTGCGCAGACAACGACGGCGCAGCCGCAGCGCGTAGCGCCTGCGGCCCGTTCGGGCTGGGA

TAACGTACCAGCGCCGGCGGAGCCGACCTACCGCTCCAACGTCAATGTTAAACATACATTTGATAACTTC

GTCGAAGGTAAATCCAACCAACTGGCGCGCGCGGCGGCACGTCAGGTGGCGGATAATCCTGGCGGCGCCT

ATAACCCGTTATTCCTCTATGGCGGCACCGGTCTGGGTAAAACTCACCTGCTGCATGCGGTAGGTAACGG

CATTATGGCGCGCAAACCCAACGCAAAAGTCGTGTATATGCACTCCGAGCGCTTTGTGCAGGACATGGTA

AAAGCCCTGCAAAATAACGCCATCGAAGAGTTTAAACGCTACTACCGCTCCGTTGACGCATTGCTGATCG

ACGATATTCAATTCTTCGCCAATAAAGAACGATCCCAGGAAGAGTTTTTCCATACCTTTAACGCTCTGCT

GGAAGGCAATCAGCAGATCATTTTGACGTCGGATCGCTATCCGAAAGAGATCAACGGCGTTGAGGATCGT

CTCAAGTCCCGCTTTGGTTGGGGGCTGACAGTGGCGATCGAACCACCAGAGCTGGAAACCCGCGTGGCGA

TCCTGATGAAAAAAGCGGACGAAAATGATATTCGTCTGCCAGGCGAGGTCGCGTTCTTTATCGCCAAACG

TCTACGCTCTAACGTGCGTGAACTGGAAGGCGCGTTGAACCGGGTGATCGCCAATGCCAACTTTACCGGC

CGGGCGATTACTATCGACTTTGTGCGCGAAGCGCTGCGCGATTTACTGGCGTTGCAGGAAAAACTGGTCA

CCATCGACAATATTCAGAAGACGGTGGCCGAGTATTACAAAATCAAAATTGCGGATCTGCTTTCTAAGCG

TCGATCTCGCTCGGTAGCACGTCCGCGCCAGATGGCTATGGCGCTGGCAAAAGAGCTCACCAACCACAGT

CTGCCGGAAATCGGCGATGCGTTTGGCGGGCGCGACCACACTACCGTACTTCATGCCTGTCGTAAAATTG

AGCAACTGCGTGAAGAAAGCCACGATATCAAAGAAGATTTTTCGAATTTAATCAGAACATTGTCGTCGTG

ATCCTATGAAATTTACCGTTGAACGTGAACATTTATTAAAACCGCTTCAGCAGGTCAGCGGCCCGCTGGG

>PX055691.1 Klebsiella pneumoniae subsp. pneumoniae strain OSR - 22 16S ribosomal RNA gene, partial sequence

GTATGATCCTGGCTCAGATTGAACGCTGGCGGCAGGCCTAACACATGCAAGTCGAGCGGTAGCACAGAGA

GCTTGCTCTCGGGTGACGAGCGGCGGACGGGTGAGTAATGTCTGGGAAAACTGCCTGATGGAGGGGGATA

ACTACTGGAAACGGTAGCTAATACCGCATAACGTCGCAAGACCAAAGTGGGGGACCTTCGGGCCTCATGC

CATCAGATGTGCCCAGATGGGATTAGCTGGTAGGTGGGGTAACGGCTCACCTAGGGGACGATCCCTAGCT

GGTCTGAGAGGATGACCAGCCACACTGGAACTGAGACACGGTCCAGACTCCTACGGGAGGCAGCAGTGGG

GAATATTGCACAATGGGCGCAAGCCTGATGCAGCCATGCCGCGTGTGTGAAGAAGGCCTTCGGGTTGTAA

AGCACTTTCAGCGGGGAGGAAGGCGGTGAGGTTAATAACCTCATCGATTGACGTTACCCGCAGAAGAAGC

ACCGGCTAACTCCGTGCCAGCAGCCGCGGTAATACGGAGGGTGCAAGCGTTAATCGGAATTACTGGGCGT

AAACGCACGCAGGGCGGTCTGTCAAGTCGGATGTGAAATCCCCGGGCTCAACCTGGGAACTGCATTCAAA

ACTTGGCACGCTAGAGTCTTGTAGAGGGGGGTAGAATTCCAAGTGTAGCGGTGAAATGCGTAGAGATCTG

GAGGAATACCGGTGGCGAAGGCGGCCCCCTGGACAAAGACTGACGCTCAGGTGCGAAAGCGTGGGGAGCA

AACAGGATTAGATACCCTGGTAGTCCACGCTGTAAACGATGTCGATTTGGAGGTTGTGCCCTTGAGGCGT

GGCTTCCGGAGCTAACGCGTTAAATCGACCGCCTGGGGAGTACGGCCGCAAGGTTAAAACTCAAATGAAT

TGACGGGGGCCCGCACAAGCGGTGGAGCATGTGGTTTAATTCGATGCAACACGAAGAACCTTACCTGGTC

TTGACATCCACAGAACTTAGCAGAGATGCTTTGGTGCCTTCGGGAACTGTGAGACAGGTGCTGCATGGCT

GTCGTCAGCTCGTGTTGTGAAATGTTGGGTTAAGTCCCGCAACGAGCGCAACCCTTATCCTTTGTTGCCA

GCGGTTCGGCCGGGAACTCAAAGGAGACTGCCAGTGATAAACTGGAGGAAGGTGGGGATGACGTCAAGTC

ATCATGGCCCTTACGACCAGGGCTACACACGTGCTACAATGGCATATACAAAGAGAAGCGACCTCGCGAG

AGCAAGCGGACCTCATAAAGTATGTCGTAGTCCGGATTGGAGTCTGCAACTCGACTCCATGAAGTCGGAA

TCGCTAGTAATCGTAGATCAGAATGCTACGGTGAATACGTTCCCGGGCCTTGTACACACCGCCCGTCACA

CCATGGGAGTGGGTTGCAAAAGAAGTAGGTAGCTTAACCTTCGGGAGGGCGCTTACCACTTTGTGATTCA

TGACTGGGGTGAAGTCGTAACAAGGAT

>CP196294.1 Klebsiella pneumoniae strain CPO307 chromosome, complete genome

ATTCGAGCACAAGATCCTCGAGCGGGTGGCCCGCATCCGCGCCGACGATCTGTTTGCCGACGACACCAAC

TTCGGCCCGCTGGTCAGCTTCCCCCATCGCGACAACGTTCTGCGCTATATCGAGAGCGGCAAACGCGAAG

GCGCGCGCCTGCTGTGCGGCGGCGAGGCGCTGAAAGGCGACGGTTTCGATAACGGCGCGTGGGTCGCCCC

GACGGTGTTTACCGATTGCAGCGACGAGATGACCATCGTGCGCGAAGAGATCTTCGGCCCGGTGATGTCG

ATCCTCAGCTATGCCGACGAAGCGGAGGTCATTCGCCGCGCCAACGCCACCGAATACGGCCTCGCCGCCG

GCGTGGTGACGCCCAACCTCAACCGCGCTCACCGAATTATCCACCAGCTGGAAGCCGGCATCTGCTGGAT

CAACAGCTGGGGGGAATCCCCGGCGGAAATGCCGGTTGGCGGCTACAAACACTCCGGCATTGGCCGCGAG

AACGGCGTCATGACGCTGCAGAGTTACACCCAGGTGAAGTCCATCCAGGTTGAGATGGGTAAATTCCAGT

CCATATTTTAACCGGGAGGTTTTTTTGCAATTTGACTACATCGTTATTGGTGCCGGCTCCGCCGGCAACG

TTCTGGCAACACGACTGACTGAAGATCCAAACACCACCGTCCTGCTGCTGGAAGCCGGCGGCCCTGACTA

CCGGTTCGATTTCCGCACCCAGATGCCCGCCGCGCTGGCTTATCCGCTGCAGGGCAAGCGCTACAACTGG

GCCTACGAGACCGAACCTGAGCCGTACATGAACAATCGCCGCATGGAGTGCGGACGCGGCAAAGGGCTCG

GCGGATCCTCGCTGATCAACGGCATGTGCTACATTCGCGGTAACGCGATGGATCTGGATAACTGGGCCAA

AGAGCCGGGTCTGGAACACTGGAGCTATCTCGACTGCCTGCCCTACTACCGGAAAGCGGAAACCCGCGAT

ATCGGGCCTAACGACTACCACGGCGGCGATGGCCCGGTGAGCGTGACGACGCCGAAGCCGGGCAATAATC

CGCTCTTCGAGGCGATGGTTGAAGCCGGGGTGCAGGCGGGCTATCCGCGTACCGACGATCTCAACGGCTA

CCAGCAGGAAGGCTTTGGCCCGATGGACCGCACCGTGACGCCGCAGGGCCGTCGCGCCAGCACCGCCCGC

GGCTATCTCGACCAGGCGCGCGGTCGCCCGAACCTGACCATCCGCACCCATGCGCTGACCGATCACATTA

TTTTTGCCGGCAAGCGCGCGGTGGGCGTCGAGTGGCTGGAGGGAGAGAGCACGATCCCGTCAAAAGCGAC

GGCCAACAAAGAGGTGCTGCTGTGCGCCGGGGCGATTGCCTCGCCGCAGATCCTGCAGCGCTCCGGAGTG

GGTAACCCGGAGCTGCTCAGGCAGTTCGATATCCCGGTGGTGCACGATCTCCCCGGCGTGGGTGAGAACC

TGCAGGACCATCTGGAGATGTATCTGCAGTACGAGTGCAAAGAGCCGGTATCGCTCTACCCGGCGCTACA

GTGGTGGAACCAGCCGAAGATCGGCGCCGAGTGGCTGTTCGGCGGCACCGGCATCGGCGCCAGCAACCAG

TTCGAAGCGGGCGGTTTTATCCGCAGCCGCGCAGAATTCGCCTGGCCGAACATTCAGTACCACTTCCTGC

CGGTGGCGATTAACTACAATGGCTCCAACGCGGTGAAAGAGCACGGCTTCCAGTGCCACGTCGGCTCGAT

GCGTTCGCCCAGCCGCGGCCACGTGCGCCTGAAGTCGCGCGACCCGCACGCCCATCCGGCGATCCTGTTT

AACTATATGTCCCATGAGCAGGACTGGCAGGAGTTCCGCGACGCCATCCGCATTACCCGGGAGATCATGA

ACCAGCCGGCGCTGGATAAGTATCGCGGCCGCGAAATCAGTCCGGGAATAGAATGCCAGAGCGATGCCGA

ACTGGATGAGTTTGTGCGCAACCACGCCGAGACCGCGTTCCACCCGTGCGGGACCTGTAAGATGGGTTAC

GACGAGATGGCGGTGGTCGATGGCGAGGGCCGCGTCCACGGTCTGGAAGGATTGCGGGTGGTCGACGCCT

CGATCATGCCGCAGATCATCACCGGCAACCTCAACGCCACCACCATTATGATCGGCGAGAAGATGGCCGA

CGCCATTCGCGGCCGCCAGCCGCTGCCGCGCAGCACCGCGACGTATTATGTCGCTGGCGACGCGCCGGTT

CGCCGCTAACAGGTCAATGCCGGGCGGCGACTACGCCTTGCCCGGCCTACCCTGAGGCGATGTAACGTAG

CCCCGGTAAGCGCAGCGCGACCGGGGTGCCGTTCCGGGTTTTGACCCCATAGGTTGATGTTAGCGAAACG

>CP171510.1 Klebsiella pneumoniae strain KP30_UID2108280026 chromosome, complete genome

TCTATAATGCGCCTCCACTGACACGGAACAACGGCAAACAAGCCGCCGGGCCAGGAGGATTCAGCGACGG

AAATCGACGTTGAAAGCCGACAGAGAAAAGCAAAATAAGCGCTTGACTCTGAATGAGGAAAACGTATTAT

ACGGGACCTCGCGACAGAGCGCTAAAGCGCGTCGCAACTGCTCTTTAACAATTTATCAGACAATCTGTGT

GGGCACTCAAAGTGACATGGATTCTTAACGTCCTCGGACGAAAAATGAATACCAAGTCTCAAGAGTGAAC

ACGTAATTCATTACGAAGTTTAATTCTTTGAGCATCAAACTTAAATTGAAGAGTTTGATCATGGCTCAGA

TTGAACGCTGGCGGCAGGCCTAACACATGCAAGTCGAGCGGTAGCACAGAGAGCTTGCTCTCGGGTGACG

AGCGGCGGACGGGTGAGTAATGTCTGGGAAACTGCCTGATGGAGGGGGATAACTACTGGAAACGGTAGCT

AATACCGCATAATGTCGCAAGACCAAAGTGGGGGACCTTCGGGCCTCATGCCATCAGATGTGCCCAGATG

GGATTAGCTAGTAGGTGGGGTAACGGCTCACCTAGGCGACGATCCCTAGCTGGTCTGAGAGGATGACCAG

CCACACTGGAACTGAGACACGGTCCAGACTCCTACGGGAGGCAGCAGTGGGGAATATTGCACAATGGGCG

CAAGCCTGATGCAGCCATGCCGCGTGTGTGAAGAAGGCCTTCGGGTTGTAAAGCACTTTCAGCGGGGAGG

AAGGCGTTAAGGTTAATAACCTTGGCGATTGACGTTACCCGCAGAAGAAGCACCGGCTAACTCCGTGCCA

GCAGCCGCGGTAATACGGAGGGTGCAAGCGTTAATCGGAATTACTGGGCGTAAAGCGCACGCAGGCGGTC

TGTCAAGTCGGATGTGAAATCCCCGGGCTTAACCTGGGAACTGCATTCGAAACTGGCAGGCTAGAGTCTT

GTAGAGGGGGGTAGAATTCCAGGTGTAGCGGTGAAATGCGTAGAGATCTGGAGGAATACCGGTGGCGAAG

GCGGCCCCCTGGACAAAGACTGACGCTCAGGTGCGAAAGCGTGGGGAGCAAACAGGATTAGATACCCTGG

TAGTCCACGCCGTAAACGATGTCGATTTGGAGGTTGTGCCCTTGAGGCGTGGCTTCCGGAGCTAACGCGT

TAAATCGACCGCCTGGGGAGTACGGCCGCAAGGTTAAAACTCAAATGAATTGACGGGGGCCCGCACAAGC

GGTGGAGCATGTGGTTTAATTCGATGCAACGCGAAGAACCTTACCTGGTCTTGACATCCACAGAACTTAG

CAGAGATGCTTTGGTGCCTTCGGGAACTGTGAGACAGGTGCTGCATGGCTGTCGTCAGCTCGTGTTGTGA

AATGTTGGGTTAAGTCCCGCAACGAGCGCAACCCTTATCCTTTGTTGCCAGCGGTTAGGCCGGGAACTCA

AAGGAGACTGCCAGTGATAAACTGGAGGAAGGTGGGGATGACGTCAAGTCATCATGGCCCTTACGACCAG

GGCTACACACGTGCTACAATGGCATATACAAAGAGAAGCGACCTCGCGAGAGCAAGCGGACCTCATAAAG

TATGTCGTAGTCCGGATTGGAGTCTGCAACTCGACTCCATGAAGTCGGAATCGCTAGTAATCGTAGATCA

GAATGCTACGGTGAATACGTTCCCGGGCCTTGTACACACCGCCCGTCACACCATGGGAGTGGGTTGCAAA

AGAAGTAGGTAGCTTAACCTTCGGGAGGGCGCTTACCACTTTGTGATTCATGACTGGGGTGAAGTCGTAA

CAAGGTAACCGTAGGGGAACCTGCGGTTGGATCACCTCCTTACCTTAAAGAACCTGCCTTTGTAGTGCTC

ACACAGATTGTCTGATGAACGTAAAGAAGCAAGGCGTCTTGCGATTGAGACTTCAGTGTCCCCTTCGTCT

AGAGGCCCAGGACACCGCCCTTTCACGGCGGTAACAGGGGTTCGAATCCCCTAGGGGACGCCACTTGCTG

GTTCGTGAGTGAAAGACGCGTGCCGAACTATCTCAAAACTCATCTTCGGGTGACGTTTGAGATATTTGCT

CTTTAAAAATCTGGATCAAGCTGAAAATTGAAACGACACACAGTTAATGTGTGTTCGAGTCTCTCAAATT

TTCGCAATCAGAAGTGAAACATCTTCGGGTTGTGAGGTTAAGCGACTAAGCGTACACGGTGGATGCCCTG

GCAGTCAGAGGCGATGAAGGACGTGCTAATCTGCGAAAAGCGTCGGTAAGGTGATATGAACCGTTATAAC

CGGCGATGTCCGAATGGGGAAACCCAGTGTGATTCGTCACACTATCGTTAACTGAATACATAGGTTAACG

AGGCGAACCGGGGGAACTGAAACATCTAAGTACCCCGAGGAAAAGAAATCAACCGAGATTCCCCCAGTAG

CGGCGAGCGAACGGGGAGCAGCCCAGAGTCTGAATCAGCTTGTGTGTTAGTGGAACGGTCTGGAAAGTCC

GACGGTACAGGGTGATAGTCCCGTACACCAAAATGCACAGGCTGTGAACTCGAAGAGTAGGGCGGGACAC

GTGGTATCCTGTCTGAATATGGGGGGACCATCCTCCAAGGCTAAATACTCCTGACTGACCGATAGTGAAC

CAGTACCGTGAGGGAAAGGCGAAAAGAACCCCGGCGAGGGGAGTGAAAAAGAACCTGAAACCGTGTACGT

ACAAGCAGTGGGAGCACCTTCGGGTGTGACTGCGTACCTTTTGTATAATGGGTCAGCGACTTATATTCTG

TAGCAAGGTTAACCGTATAGGGGAGCCGCAGGGAAACCGAGTCTTAACTGGGCGTTAAGTTGCAGGGTAT

>CP196638.1 Klebsiella pneumoniae strain CX36 chromosome, complete genome

GCTTACGGGCACAAGCGATATAATGCCGCTCAATTCCTTTTTTTTTCTCATCTTTATTGGTTGGAACTGA

GCCATAAATGTAATCTGGCCATAATATCAGCTCTTTAAAGATCCTATTGTTTATCCTTTTTTGAATACGC

CTTATTAGCTGGTGTGGATCATAAACATCTCTAAGTTCACCATTTATTTTTTTTACTTGCTTTTTCTTGT

ATCTTTGGTGGGAAGGCATTGCAATAGCAGTTTTTAAATCTGCTAACGTAATATCCAAGCAAGAACTTAA

AGATTGCAAAGATCTGAGAGATTGTGCACCAGGTTTTTTTATAGGATTACGGGCAACCATAATATCTTCC

ATTCCATGAAATCAAAATTACATCCGGCCTCCCTCATACTCAAGTGGGCAAAAGAATAGGGTTGCTTTCG

TCAAAAGCCCTTAGCAACTGCCCCTATTCTTTGACCGGACGCAATTGAGGACGGCTACAGCCCCATCGTT

GCCGGCCAGGCTTTGTGGGGGTTCCGTTCCAGCCTATGCTACTGCATAGGCGCTCTCACTTTTCTGTTGC

GAGAGCGGAAGCTAAGAAACTATAAACTTTGACTGCCACAGGCAAACGGAACGTTTGCGCGAAGCAGATA

TTACCATTGATGTTTCTAGTTTCAAGACTTAACTAGCATTTTTCACATCACCTACCAAAATTGTAATGGA

CTGACCAAAAAGGCACTACACCGCACCCGCCTGCGGTTTTCATATCACTAAAATTTTTCAGTTGTGATTT

TTTACAAACGATACCGCCAGCCCGCGCCCGTGCTGGGGATACACTGCGAGGCAGGAACTGAAAAGAATGA

AAAGAAATTCAGCTTTTTTCAGTTTTACTGTCCGTAGTATCTATGGCCTGAATACGCAACTCTATGATAT

TAAATGGATAATTATTTTTAGGTGTGTGATTTTATGAAGTTTTGGAAACATGAAAAACATAACAAATTGA

AAAATAATAATTTTATGTCCGTATAAACTGAAATCCGGTGTTCAGGTTCCGGGAATGGATCGGCCATTCC

CGACGCATACTTTACCCATTCTTTTGCGCAGCCAGCAGCAACTTTAAAGCCTTTTCCCGTTCCTCGGGTG

GAATTGCATCAATTAGCTCTTTAACCCTCCCCTGCCCGTTCAGCGCGCTTGGACTAACGGAATGGGAGAA

AGCAACATTCATCACAAACGAGTGACCGCACTCCACATTAGAGCAATAGCAGTACACATCGTACAGTTGT

GGGTGTTTCCTGTTTGTTTTACCTATCCGCGCTACTGCCTGACATGTGGGGCAGTACATTCTCATCATCT

TCATCGTTCTGTGCCCTATACTGTTGCCCGCCAGAAGAAGGGATTTGACTCCCTACCTCTTGTTCTGAAC

GTGTAACACTGGTAACACGTGTAACGCTATTGATTTAAAAGGATTTAATCTGTTACCAGTTAGCAAAATC

AAGAGGTAACGCGTGGTAACACACCATTTTTTGTTACCTCTGTTACCAGTCAGTATTTTTGACTGGTAAC

ACTGAAGAACCGTATCAGTAGCGGGTGTTACCTCTGTTACCAGTGTTACCTCATATAAATAAGACTCACG

CGATAATTACTCAGGTTCGGCGCTTCCCAGTACCTGTGTATTGAACTGGTACACACGTTTAAGCCCTATT

TCTGGCAGGCGAATGCTGTTTTGCGTCCGGCCATCTTCACCGGGTTTCAGCCAGCCCGCGTCCACACACA

ACCTGGCCACCTTACGCGAATCAAATCCCTTACAGATCTCCTTCCAGCCTGACGGGAGAACGTAGAACGT

CACAACCGGCTCTGTCACGTTGTCTCCTTTATCGACTTTTCTGAATCCCATCATTGAAACAGGCCGGTTC

CTGTCGTCATTCCAGTCAGCAAACCGACTGAACTGATTACGCGTCATGAAGTCCCGTACCTGTTCAAGTG

CCGCTTTATCTTCCTGATTAGCTGTATGGCCCCGGTCAGCCATCCATGCAGCCAGACAACTTTGAGCGGC

ACGAAAGGCCTCTCCCTCCGGCCATCCTGTGATGCCCGCTTTTGTGGCCAGCTCCCCGGCCATAGCAACC

AGAGCAAAACGGGTAACAGCCCGGCCAACCTGATTCCCGGCATTTTCCGGCGTCAGCCTCCGGGTGTACT

CTTTCAATAAGGCTTTCGCCTGGCTGGTCAGTTCCGGCAGATCAGCGGTCAGGCAATGCAGCCAGTCGCG

GAATGGTGCACCATGATAATGCGCTACATGCTGCTCAAGATGCTCAGCCAGGGTTTTCCCGCTACTGAAG

CCATGAAGTTCTTCAAACACGCCATACTTGCCCGAATCGCTTGGGATCTGGATCATTCTGACCTCAACCC

CGGCATACGTACGTTCCCCGGCGCTTGCCGCATGTTCTACCAGTGACAGTTCCCCGGTGGACAGAAACAA
