# Supplementary material for: Molecular Identification of Culturable Bacteria and Vibrio Virulence‐Related Genes Associated With Sea Turtle Eggshells and Nest Sediments in Pangandaran, Indonesia
Source: Int J Microbiol. 2026 Oct 5;2026:7422673. doi: 10.1155/ijm/7422673 (PMC13639395; doi:10.1155/ijm/7422673)
Supplement: Supplementary file 2 [file IJM-2026-7422673-s002.zip › Hasil NCBI/CS consensus.docx]

>KT260919.1 Enterobacter asburiae strain RCB707 16S ribosomal RNA gene, partial sequence

GGAAACGGTAGCTAATACCGCATAACGTCGCAAGACCAAAGAGGGGGACCTTTCGGGCCTCTTGCCATCA

GATGTGCCCAGATGGGATTAGCTAGTAGGTGGGGTAACGGCTCACCTAGGCGACGATCCCTAGCTGGTCT

GAGAGGATGACCAGCCACACTGGAACTGAGACACGGTCCAGACTCCTACGGGAGGCAGCAGTGGGGAATA

TTGCACAATGGGCGCAAGCCTGATGCAGCCATGCCGCGTGTATGAAGAAGGCCTTCGGGTTGTAAAGTAC

TTTCAGCGGGGAGGAAGGTGTTGAGGTTAATAACCTCAGCAATTGACGTTACCCGCAGAAGAAGCACCGG

CTAACTCCGTGCCAGCAGCCGCGGTAATACGGAGGGTGCAAGCGTTAATCGGAATTACTGGGCGTAAAGC

GCACGCAGGCGGTCTGTCAAGTCGGATGTGAAATCCCCGGGCTCAACCTGGGAACTGCATTCGAAACTGG

CAGGCTAGAGTCTTGTAGAGGGGGGTAGAATTCCAGGTGTAGCGGTGAAATGCGTAGAGATCTGGAGGAA

TACCGGTGGCGAAGGCGGCCCCCTGGACAAAGACTGACGCTCAGGTGCGAAAGCGTGGGGAGCAAACAGG

ATTAGATACCCTGGTAGTCCACGCCGTAAACGATGTCGACTTGGAGGTTGTGCCCTTGAGGCGTGGCTTC

CGGAGCTAACGCGTTAAGTCGACCGCCTGGGGAGTACGGCCGCAAGGGTAAAACTCAAATGAATTGACGG

GGGCCCGCACAAGCGGTGGAGCATGTGGTTTAATTCGATGCAACGCGAAGAACCTTACCTACTCTTGACA

TCCAGAGAACTTTCCAGAGATGGATTGGTGCCTTCGGGAACTCTGAGACAGGTGCTGCATGGCTGTCGTC

AGCTCGTGTTGTGAAATGTTGGGTTAAGTCCCGCAACGAGCGCAACCCTTATCCTTTGTTGCCAGCGGTT

CGGCCGGGAACTCAAAGGAGACTGCCAGTGATAAACTGGAGGAAGGTGGGGATGACGTCAAGTCATCATG

GCCCTTACGAGTAGGGCTACACACGTGCTACAATGGCGCATACAAAGAGAAGCGACCTCGCGAGAGCAAG

CGGACCTCATAAAGTGCGTCGTAGTCCGGATTGGAGTCTGCAACTCGACTCCATGAAGTCGGAA

>PV014873.1 Kosakonia cowanii strain WTB002 16S ribosomal RNA gene, partial sequence

GGGCGGCGGCTAACCTGCAAGTCGAACGGTAACAGGAAGCAGCTTGCTGCTTCGCTGACGAGTGGCGGAC

GGGTGAGTAATGTCTGGGAAACTGCCTGATGGAGGGGGATAACTACTGGAAACGGTAGCTAATACCGCAT

AACGTCGCAAGACCAAAGAGGGGGACCTTCGGGCCTCTTGCCATCAGATGTGCCCAGATGGGATTAGCTA

GTAGGTGGGGTAACGGCTCACCTAGGCGACGATCCCTAGCTGGTCTGAGAGGATGACCAGCCACACTGGA

ACTGAGACACGGTCCAGACTCCTACGGGAGGCAGCAGTGGGGAATATTGCACAATGGGCGCAAGCCTGAT

GCAGCCATGCCGCGTGTATGAAGAAGGCCTTCGGGTTGTAAAGTACTTTCAGCGGGGAGGAAGGCGATGT

GGTTAATAACCGCGTCGATTGACGTTACCCGCAGAAGAAGCACCGGCTAACTCCGTCCCACCAGCCCCGG

TAATACGGAGGGTGCAAGCGTTAATCGGAATTACTGGGCGTAAAGCGCACGCAGGCGGTCTGTCAAGTCG

GATGTGAAATCCCCGGGCTCAACCTGGGAACTGCATCCGAAACTGGCAGGCTTGAGTCTCGTAGAGGGGG

GTAGAATTCCAGGTGTAGCGGTGAAATGCGTAGAGATCTGGAGGAATACCGGTGGCGAAGGCGGCCCCCT

GGACGAAGACTGACGCTCAGGTGCGAAAGCGTGGGGAGCAAACAGGATTAGATACCCTGGTAGTCCACGC

CGTAAACGATGTCGACTTGGAGGTTGTGCCCTTGAGGCGTGGCTTCCGGAGCTTAACGCGTTAAGTCGAC

CGCCTGGGGAGTACGGCCGCAAGGGTAAAACTCAAATGAATTGACGGGGGCCCGCACAAGCGGGGGAGCA

TGTGGTTTAATTCGATGCAACGCGAAGAACCTTACCTGGTCTTGACATCCACAGAACTTGGCAGAGATGC

CTTGGTGCCTTCGGGAACTGTGAGACAGGTGCTGCATGGCTGTCGTCAGCTCGTGTTGTGAAATGTTGGG

TTAAGTCCCGCAACGAGCGCAACCCTTATCCTTTGTTGCCAGCGGTTCGGCCGGGAACTCAAAGGAGACT

GCCAGTGATAAACTGGAGGAAGGTGGGGATGACGTCAAGTCATCATGGCCCTTACGACCAGGGCTACACA

CGTGCTACAATGGCGCATACAAAGAGAAGCAATCTCGCGAGAGCAAGCGGACCTCATAAAGTGCGTCGTA

GTCCGGATTGGAGTCTGCAACTCGACTCCATGAAGTCGGAATCGCTAGTAATCGTGAATCAGAATGTCAC

GGTGAATACGTTCCCGGGCCTTGTACACACCGCCCGTCACACCATGGGAGTGGGTTGCAAAAGAAGTAGG

TAGCTTAACCTTCGGGAGGGCGCTTACCACTTGTATACAGA

>OM243108.1 Klebsiella pneumoniae subsp. rhinoscleromatis strain MS1 16S ribosomal RNA gene, partial sequence

AAGAAGGGGCGGCCTAACACATGCAAGTCGAGCGGTAGCACAGAGAGCTTGCTCTCGGGTGACGAGCGGC

GGACGGGTGAGTAATGTCTGGGAAACTGCCTGATGGAGGGGGATAACTACTGGAAACGGTAGCTAATACC

GCATAACGTCGCAAGACCAAAGTGGGGGACCTTCGGGCCTCATGCCATCAGATGTGCCCAGATGGGATTA

GCTAGTAGGTGGGGTAACGGCTCACCTAGGCGACGATCCCTAGCTGGTCTGAGAGGATGACCAGCCACAC

TGGAACTGAGACACGGTCCAGACTCCTACGGGAGGCAGCAGTGGGGAATATTGCACAATGGGCGCAAGCC

TGATGCAGCCATGCCGCGTGTGTGAAGAAGGCCTTCGGGTTGTAAAGCACTTTCAGCGGGGAGGAAGGCG

ATAAGGTTAATAACCTTGTCGATTGACGTTACCCGCAGAAGAAGCACCGGCTAACTCCGTGCCAGCAGCC

GCGGTAATACGGAGGGTGCAAGCGTTAATCGGAATTACTGGGCGTAAAGCGCACGCAGGCGGTCTGTCAA

GTCGGATGTGAAATCCCCGGGCTCAACCTGGGAACTGCATTCGAAACTGGCAGGCTAGAGTCTTGTAGAG

GGGGGTAGAATTCCAGGTGTAGCGGTGAAATGCGTAGAGATCTGGAGGAATACCGGTGGCGAAGGCGGCC

CCCTGGACAAAGACTGACGCTCAGGTGCGAAAGCGTGGGGAGCAAACAGGATTAGATACCCTGGTAGTCC

ACGCCGTAAACGATGTCGATTTGGAGGTTGTGCCCTTGAGGCGTGGCTTCCGGAGCTAACGCGTTAAATC

GACCGCCTGGGGAGTACGGCCGCAAGGTTAAAACTCAAATGAATTGACGGAGGCCCGCACAAGCGGTGGA

GCATGTGGTTTAATTCGATGCAACGCGAAGAACCTTACCTGGTCTTGACATCCACAGAACTTACCAAAGA

TGCATTGGTGCCTTCGGGAACTGTGAAACAGGTGCTGCATGGCTGTCGTCAGCTCGTGTCGTCGAAATGT

TGGATTAAGNTCCAGCAACCAGCGCAACCCTTATCCTTTGTTGCCAGCGGTTAGGCCGGGAACTCAAAGA

AGACTGCCAGTAATAAACTGCAGGAACGTGGGAATGAAGTCAAGTCATCATGGCCCTTACGACCAGGGCT

ACACACGTGCTACAATGGCATATACAAAGAGAAGCGACCTCGCGAGAGCAAGCGGACCTCATAAAGTATG

TCGTAGTCCGGATTGGAGTCTGCAACTCGACTCCATGAAGTCGGAATCGCTAGTAATCGTAGATCAGAAT

GCTACGGTGAATACGTTCCCGGGCCTTGTACACACCGCCCGTCACACCATGGGAGTGGGTTGCAAAAGAA

GTAGGTAGCTTAACCTTCGGGAGGGCGCTTCCCATCGTGTT

>OQ979237.1 Erwinia sp. strain IP1 16S ribosomal RNA gene, partial sequence

CCTGGGGGGGCCTACACATGCAAGTCGAACGGCAGCACAGAAGAGCTTGCTCTTTGGGTGGCGAGTGGCG

GACGGGTGAGTAATGTCTGGGAAACTGCCCGATGGAGGGGGATAACTACTGGAAACGGTAGCTAATACCG

CATAACGTCGCAAGACCAAAGTGGGGGACCTTCGGGCCTCACACCATCGGATGTGCCCAGATGGGATTAG

CTAGTAGGTGGGGTAATGGCTCACCTAGGCGACGATCCCTAGCTGGTCTGAGAGGATGACCAGCCACACT

GGAACTGAGACACGGTCCAGACTCCTACGGGAGGCAGCAGTGGGGAATATTGCACAATGGGCGCAAGCCT

GATGCAGCCATGCCGCGTGTATGAAGAAGGCCTTCGGGTTGTAAAGTACTTTCAGCGGGGAGGAAGGCGG

TGAGGTTAATAACCTCGCCGATTGACGTTACCCGCAGAAGAAGCACCGGCTAACTCCGTGCCAGCAGCCG

CGGTAATACGGAGGGTGCAAGCGTTAATCGGAATTACTGGGCGTAAAGCGCACGCAGGCGGTCTGTTAAG

TCAGATGTGAAATCCCCGGGCTTAACCTGAGAACTGCATGTGAAACTGGCAGGCTTGAGTCTCGTAGAGG

GGCGTAGAATTCCAGGTGTAGCGGTGAAATGCGTAGAGATCTGGAGGAATACCGGTGGCGAAGGCGGCCC

CCTGGACGAAGACTGACGCTCAGGTGCGAAAGCGTGGGGAGCAAACAGGATTAGATACCCTGGTAGTCCA

CGCCGTAAACGATGTCGACTTGGAGGTTGTGCCCTTGAGGCGTGGCTTCCGGAGCTAACGCGTTAAGTCG

ACCGCCTGGGGAGTACGGCCGCAAGGTTAAAACTCAAATGAATTGACGGAGGCCCGCACAAGCGGTGGAG

CATGTGGTTTAATTCGATGCAACGCGAAGAACCTTACCTGGCCTTGACATCCCAGAGAACTTAACAAAGA

TGCTTTGGTGCCTTCGGGAACTCTGAAACAGGTGCTGCATGGCTGTCGTCAGCTCGTGTTGTGAAATGTT

GGGTTAAGTCCCGCAACGAGCGCAACCCTTATCCTTTGTTGCCAGCGGTTCGGCCGGGAACTCAAAGGAG

ACTGCCGGTGATAAACCGGAGGAAGGTGGGGATGACGTCAAGTCATCATGGCCCTTACGGCCAGGGCTAC

ACACGTGCTACAATGGCGCATACAAAGAGAAGCGACCTCGCGAGAGCAAGCGGACCTCATAAAGTGCGTC

GTAGTCCGGATTGGAGTCTGCAACTCGACTCCATGAAGTCGGAATCGCTAGTAATCGTAGATCAGAATGC

TACGGTGAATACGTTCCCGGGCCTTGTACACACCGCCCGTCACACCATGGGAGTGGGTTGCAAAAGAAGT

AGGTAGCTTAACCTTCGGGAGGGCGCTTACCCACTTGTTCAGGGA

>KT248083.1 Kluyvera georgiana strain P4607 16S ribosomal RNA gene, partial sequence

TAGCACAGAGAGCTTGCTCTCGGGTGACGAGTGGCGGACGGGTGAGTAATGTCTGGGAAACTGCCCGATG

GAGGGGGATAACTACTGGAAACGGTAGCTAATACCGCATAACGTCGCAAGACCAAAGTGGGGGACCTTCG

GGCCTCAAACCATCGGAGTTGTGCCCAGATGAGGATTAGGCTAGTAGGTGGGGTAACGGCTCACCTAGGC

GACGATCCCTAGCTGGTCTGAGAGGATGGACCAGCCACACTGGAACTGAGATCACGGTCCAGACTCCTAC

GGGAGGCAGCAGTGGGGAATATTGCACAATGGGCGCAAGCCTGATGCAGCCATGCCGCGTGTATGAAGAA

GGCCTTCGGGTTGTAAAGTACTTTCAGCGACGAGGAAGGCATTAAGGTTAATAACCTTAGTGATTGACGT

TACTCGCAGAAGAAGCACCGGCTAACTCCGTGCCAGCAGCCGCGGTAATACGGAGGGTGCAAGCGTTAAT

CGGAATTACTGGGCGTAAAGCGCACGCAGGCGGTTTGTTAAGTCAGATGTGAAATCCCCGGGCTCAACCT

GGGAACTGCATTTGAAACTGGCAAGCTTGAGTCTCGTAGAGGGGGGTAGAATTCCAGGTGTAGCGGTGAA

ATGCGTAGAGATCTGGAGGAATACCGGTGGCGAAGGCGGCCCCCTGGACGAAGACTGACGCTCAGGTGCG

AAAGCGTGGGGAGCAAACAGGATTAGATACCCTGGTAGTCCACGCCGTAAACGATGTCTATTTGGAGGTT

GTTCCCTTGAGGAGTGGCTTCCGGAGCTAACGCGTTAAATAGACCGCCTGGGGAGTACGGCCGCAAGGGT

AAAACTCAAATGAATTGACGGGGGCCCGCACAAGCGGTGGAGCATGTGGTTTAATTCGATGCAACGCGAA

GAACCCTTACCCTACTCTTGACATCCAGAGAACTTTAGCAGAGATGCTTTTGGTGCCCTTCGGGAACTCT

GAGACAGGTGCTGCATGGCTGTCGTCAGCTTCGTGTTTGTGAAATGTTGGGTTAAGTCCCGCAACGAGCG

CAACCCTTAATCCTTTGTTGCCAGCGATTCGGCCGGGAAACTCGAAAGGAGACTGCCAGGTGATAAACTG

GAAGAAGGTGGGGATGACGTCAAGTCATCATGGCCCTTACAAGTAGGCCTAACACACGTGCTACAATGGG

CCATATACAAAGAGAAGCGAACTTCGCGAGAGCAAGCGGACCTCATAAAGTATGCCTGATAGTCCGAGAA

TGGAAGATC
