# Supplementary material for: Molecular Identification of Culturable Bacteria and Vibrio Virulence‐Related Genes Associated With Sea Turtle Eggshells and Nest Sediments in Pangandaran, Indonesia
Source: Int J Microbiol. 2026 Oct 5;2026:7422673. doi: 10.1155/ijm/7422673 (PMC13639395; doi:10.1155/ijm/7422673)
Supplement: Supplementary file 2 [file IJM-2026-7422673-s002.zip › Hasil NCBI/JS Consensus.docx]

>PP106503.1 Vibrio parahaemolyticus strain CSBG 16S ribosomal RNA gene, partial sequence

ATGCCTAGGAAATTGCCCTGATGTGGGGGATAACCATTGGAAACGATGGCTAATACCGCATGATGCCTAC

GGGCCAAAGAGGGGGACCTTCGGGCCTCTCGCGTCAGGATATGCCTAGGTGGGATTAGCTAGTTGGTGAG

GTAAGGGCTCACCAAGGCGACGATCCCTAGCTGGTCTGAGAGGATGATCAGCCACACTGGAACTGAGACA

CGGTCCAGACTCCTACGGGAGGCAGCAGTGGGGAATATTGCACAATGGGCGCAAGCCTGATGCAGCCATG

CCGCGTGGGTGAAGAAGGCCTTCGGGTTGTAAAGCACTTTCAGTCGTGAGGAAGGTAGTGTAGTTAATAG

CTGCATTATTTGACGTTAGCGACAGAAGAAGCACCGGCTAACTCCGTGCCAGCAGCCGCGGTAATACGGA

GGGTGCGAGCGTTAATCGGAATTACTGGGCGTAAAGCGCATGCAGGTGGTTTGTTAAGTCAGATGTGAAA

GCCCGGGGCTCAACCTCGGAATTGCATTTGAAACTGGCAGACTAGAGTACTGTAGAGGGGGGTAGAATTT

CAGGTGTAGCGGTGAAATGCGTAGAGATCTGAAGGAATACCGGTGGCGAAGGCGGCCCCCTGGACAGATA

CTGACACTCAGATGCGAAAGCGTGGGGAGCAAACAGGATTAGATACCCTGGTAGTCCACGCCGTAAACGA

TGTCTACTTGGAGGTTGTGGCCTTGAGCCGTGGCTTTCGGAGCTAACGCGTTAAGTAGACCGCCTGGGGG

AGTACGGTCGCAAGATTAAAACTCAAATGAATTGACGGGGGCCCGCACAAGCGGTGGAGCATGTGGTTTA

ATTCGATGCAACGCGAAGAACCTTACCTACTCTTGACATCCAGAGAACTTTCCAGAGATGGATTGGTGCC

TTCGGGAACTCTGAGACAGGTGCTGCATGGCTGTCGTCACCTCGTGTTGTGAAATGTTGGGTTAAGTCCC

GCAACGAACGCAACCCTTATCCTTGTTTGCCAGCGAGTAATGTCGGGAACTCCAGGGAAACTGCCGGTGA

TAAACCGGAGGAAGGTGGGGACAACGCCAAGTCATCATGGCCCTTACAAATAAGGCTACACACGTGCTAC

AATGGCGCATACAGAGGGCGGCCAACTTGCGAAAGTGAGCGAATCCCAAAAAGTGCGTCGTAGTCCGGAT

TGGAGTCTGCAACTCGACTCCATGAAGTCGGAATCGCTAGTAATCGTGGATCAGAATGCCACGGTGAATA

CGTTCCCGGGCC

>PP389753.1 Vibrio alginolyticus strain A2 16S ribosomal RNA gene, partial sequence

GCCTACCATGCAGTCGAGCGGAACGAGTTATCTGAACCTTCGGGGAACGATAACGGCGTCGAGCGGCGGA

CGGGTGAGTAATGCCTAGGAAATTGCCCTGATGTGGGGGATAACCATTGGAAACGATGGCTAATACCGCA

TGATGCCTACGGGCCAAAGAGGGGGACCTTCGGGCCTCTCGCGTCAGGATATGCCTAGGTGGGATTAGCT

AGTTGGTGAGGTAAGGGCTCACCAAGGCGACGATCCCTAGCTGGTCTGAGAGGATGATCAGCCACACTGG

AACTGAGACACGGTCCAGACTCCTACGGGAGGCAGCAGTGGGGAATATTGCACAATGGGCGCAAGCCTGA

TGCAGCCATGCCGCGTGTGTGAAGAAGGCCTTCGGGTTGTAAAGCACTTTCAGTCGTGAGGAAGGTGGTG

TAGTTAATAGCTGCATTATTTGACGTTAGCGACAGAAGAAGCACCGGCTAACTCCGTGCCAGCAGCCGCG

GTAATACGGAGGGTGCGAGCGTTAATCGGAATTACTGGGCGTAAAGCGCATGCAGGTGGTTTGTTAAGTC

AGATGTGAAAGCCCGGGGCTCAACCTCGGAATTGCATTTGAAACTGGCAGACTAGAGTACTGTAGAGGGG

GGTAGAATTTCAGGTGTAGCGGTGAAATGCGTAGAGATCTGAAGGAATACCGGTGGCGAAGGCGGCCCCC

TGGACAGATACTGACACTCAGATGCGAAAGCGTGGGGAGCAAACAGGATTAGATACCCTGGTAGTCCACG

CCGTAAACGATGTCTACTTGGAGGTTGTGGCCTTGAGCCGTGGCTTTCGGAGCTAACGCGTTAAGTAGAC

CGCCTGGGGAGTACGGTCGCAAGATTAAAACTCAAATGAATTGACGGGGGCCCGCACAAGCGGTGGAGCA

TGTGGTTTAATTCGATGCAACGCGAAGAACCTTACCTACTCTTGACATCCAGAGAACTTTCCAGAGATGG

ATTGGTGCCTTCGGGAACTCTGAGACAGGTGCTGCATGGCTGTCGTCAGCTCGTGTTGTGAAATGTTGGG

TTAAGTCCCGCAACGAGCGCAACCCTTATCCTTGTTTGCCACCGAGTAATGTCGGGAACTCCAGGGAAAC

TGCCGGTGAATAACCGGAGGAAGGTGGGGACAACTTCAAGTCATCATGGCCCTTACAAATAGGGCTACAC

ACGTGCTACAATGGCCCATACAGAGGGCGGCCAACTTGCAAATGTGAACCAATCCCAAAAGGCGCGCTCT

ATTCCGGATTGGAGTTCCCACCCCCTCTCCATGAAAACGGAACCCTTAGTAATTTGGGAATAGAATGCCC

CCGGGGAAACGTTCCCCGGCTTTTAACCCCCCCCCCCCCACACCATGGGAGTGGGCTGCAAAAGAAGTAG

GTAGTTTAACCTTCGGGGGGACGCTACCATTGA

>PP116102.1 Vibrio parahaemolyticus strain HSEMS 16S ribosomal RNA gene, partial sequence

GGTGAGTAATGCCTAGGAAATTGCCCTGATGTGGGGGATAACCATTGGAAACGATGGCTAATACCGCATG

ATGCCTACGGGCCAAAGAGGGGGACCTTCGGGCCTCTCGCGTCAGGATATGCCTAGGGGGGATAAGCTAG

TTGGTGAGGTAAGGGCTCACCAAGGCGACGATCCCTAGCTGGTCTGAGAGGATGATCAGCCACACTGGAA

CTGAGACACGGTCCAGACTCCTACGGGAGGCAGCAGTGGGGAATATTGCACAATGGGCGCAAGCCTGATG

CAGCCATGCCGCGTGTGTGAAGAAGGCCTTCGGGTTGTAAAGCACTTTCAGTCGTGAGGAAGGTAGTGTA

GTTAATAGCTGCATTATTTGACGTTAGCGACAGAAGAAGCACCGGCTAACTCCGTGCCAGCAGCCGCGGT

AATACGGAGGGTGCGAGCGTTAATCGGAATTACTGGGCGTAAAGCGCATGCAGGTGGTTTGTTAAGTCAG

ATGTGAAAGCCCGGGGCTCAACCTCGGAATTGCATTTGAAACTGGCAGACTAGAGTACTGTAGAGGGGGG

TAGAATTTCAGGTGTAGCGGTGAAATGCGTAGAGATCTGAAGGAATACCGGTGGCGAAGGCGGCCCCCTG

GACAGATACTGACACTCAGATGCGAAAGCGTGGGGAGCAAACAGGATTAGATACCCTGGTAGTCCACGCC

GTAAACGATGTCTACTTGGGAGGTTGTGGCCTTGAGCCGTGGCTTTCGGAGCTAACGCGTTAAGTAGACC

GCCTGGGGGAGTACGGTCGCAAGATTAAAACTCAAATGAATTGACGGGGGCCCGCACAAGCGGTGGAGCA

TGTGGTTTAATTCGATGCAACGCGAAGAACCTTACCTACTCTTGACATCCAGAGAACTTTCCAGAGATGG

ATTGGTGCCTTCGGGAACTCTGAAACAGGTGCTGCATGGCTGTCGTCACCTCGTGTTGTGAAATGTTGGG

TTAAGTCCCGCAACAAACGCAACCCTTATCCTTGTTTGCCACCGAATAATGTCGGGAACTCCAGGGAAAC

TGCCGGTAATAAACCGGAAGAAGGGGGGGACAACGTCAAGTCATCATGGCCCTTACAAATAAGGCTACAC

ACGTGCTACAATGGCGCATACAGAAGGCGGCCAACTTGCCAAAGTAACCGAATCCCAAAAAGTGCGTCGT

AGTCCGGATTGGAGTCTGCAACTCGACTCCATGAAGTCGGAATCGCTAGTAATCGTGGATCAGAATGCCA

CGGTGAATACGTTCCCGGGCCTTGTACACACCGCCCGTCACACCATGGGAGTG

>CP152363.1 Vibrio parahaemolyticus strain vp-HL-201910 chromosome 1, complete sequence

GTGTCATCTTCGCTTTGGCTGCAATGTTTGCAGCAGCTTCAAGAAGAGCTACCAGCTACAGAATTCAGTA

TGTGGGTTCGTCCGTTACAAGCGGAGCTCAATGACAATACTCTCACTCTATTCGCGCCAAACCGCTTTGT

GTTGGATTGGGTGCGTGATAAGTATCTGAACAGCATTACTCGTTTACTGCAGGAATACTGTGGTAACGAC

ATCCCGAATTTACGCTTTGAAGTGGGCAGTCGCCCAGTTTCTGCGCCTAAGCCTGCACCGACACGTACGC

CGGCGGATGTGGCTGCGGAATCTTCAGCGCCTGCGCAGTTGCAAGCACGTAAACCTGTGCACAAAACATG

GGATGACGACCCTCAAGCGATTGCAGCGATCAATCACCGCTCAAACATGAACCCAAAACATAAGTTCGAC

AACTTCGTAGAAGGTAAATCGAACCAACTGGGTTTGGCTGCGGCGCGTCAGGTATCGGATAACCCAGGAG

CTGCCTACAACCCACTGTTCCTTTACGGTGGTACTGGTCTAGGTAAAACTCACTTGCTTCACGCTGTGGG

TAACGCGATTGTGGATAACAACCCGAACGCGAAAGTGGTGTACATGCACTCTGAGCGTTTCGTGCAAGAC

ATGGTAAAAGCACTGCAAAACAATGCGATTGAAGAATTCAAGCGCTACTACCGCAGTGTGGATGCACTCC

TTATCGATGACATCCAATTCTTTGCCAACAAAGAGCGCTCACAAGAAGAATTCTTCCATACCTTCAACGC

ATTGTTGGAAGGTAACCAACAGATCATCCTAACTTCTGACCGTTATCCAAAAGAGATCAGCGGGGTAGAG

GATCGTCTGAAATCGCGCTTTGGTTGGGGTCTAACGGTTGCGATCGAGCCGCCTGAACTAGAGACGCGTG

TGGCGATTTTGATGAAGAAAGCTGAAGACCATCAAATTCATTTGGCTGACGAAGTCGCGTTCTTTATTGC

AAAACGTCTGCGTTCCAACGTTCGTGAGTTGGAAGGCGCGTTGAACCGAGTCATTGCCAACGCGAACTTT

ACTGGCCGCCCAATCACCATCGACTTCGTTCGCGAAGCGCTGCGCGATCTGCTGGCGCTGCAAGAGAAGT

TGGTGACCATTGACAACATTCAAAAGACGGTTGCTGAGTACTACAAAATCAAAGTGGCGGATCTGCTGTC

TAAACGTCGTTCTCGCTCCGTTGCACGTCCTCGTCAATTGGCGATGGCACTGGCTAAAGAGCTAACTAAC

CACAGCTTGCCAGAAATCGGTGATGCGTTTGGTGGCCGAGACCATACGACCGTTTTGCATGCATGTCGCA

AGATTGAGCAGCTGCGTGAAGAGAGCCACGATATTAAGGAAGATTACTCGAACTTGATTCGCACCCTGTC

TTCTTAATCGGTTAGAATAACGGTATTCTAAGCAAATCAATTTTTGAGCTGACAGCGTAAAGAGCCAAGC

ATGAAATTTACTATTGAACGTAGTCATTTAATCAAACCGCTACAACAGGTTTCGGGCGCTTTGGGTGGCC

GACCAACCCTGCCTATTTTGGGCAACCTACTGATTAAAGTAGAAGAGAGCGTGCTATCAATGACCGCGAC

TGACTTAGAAGTGGAATTGGTGAGCAAAGTGACTCTAGAAGGGGATTTCGAAGCGGGTAGCATTACCGTT

CCTTCGCGTAAGTTTCTCGATATTTGCCGTGGCTTGCCAGACGATGCGATCATCACTTTTGTGTTAGAAG

GCGATCGCGTGCAAGTTCGTTCTGGTCGCAGCCGTTTCTCACTGGCAACGCTACCAGCGAATGACTTCCC

GAACATTGAAGATTGGCAAAGTGAAGTTGAAGTATCACTGAGCCAAGCGGATCTGCGTACGCTTATCGAC

AAAACTCAGTTCTCGATGGCAAACCAAGACGTACGTTACTACCTCAACGGTATGTTGTTTGAAATCGATG

GCACAACACTGCGTAGTGTGGCGACCGACGGTCACCGTATGGCAGTATCGCAAACTCAACTAGGTGCTGA

CTTCGCCCAAAAGCAAATCATCGTGCCACGCAAAGGTGTGCAGGAATTGGTTAAGCTGATGGATGCGCCA

GAGCAACCTGTCGTTTTGCAAATTGGCAGCTCAAACGTGCGTGCAGAAGTGAACAACTTTATCTTCACGT

CTAAACTGGTTGATGGCCGTTTCCCTGACTATCGCCGCGTATTACCGCAACATACCAACAAAACACTGAT

CGCAAGTTGTGATGAATTGCGCCAAGCGTTCTCGCGTGCTGCGATTCTTTCTAACGAAAAGTTCCGTGGT

GTGCGTGTTAACTTAGCGGGCAGCGAAATGCGCATTACGGCAAACAACCCAGAGCAGGAAGAAGCCGAAG

AGATGCTGGATGTGACCTTTGAAGGTGACCCAATTGAAATCGGCTTCAACGTTAGCTACGTGTTGGATGT

GCTGAACACGCTACGTTGCGAGAAGGTACAAGTGTCGATGTCGGATGCCAATGCCAGTGCATTGATTGAA

AATGCCGACGATGACAGTGCCATGTACGTGGTGATGCCAATTCGTCTATAAGCCAACTGAATGCCTCTTT

CTCGTCTCATCATTCAGCAGTTTCGCAACATTAAAGCCTGTGATATTCAGTTATCAGCAGGCTTTAACTT

TCTTATTGGACCGAACGGTAGCGGCAAAACCAGTGTCCTAGAAGCGATTTATTTACTCGGGCATGGTCGC

TCATTCAAAAGCTCTCTCACTGGTCGTGTGATTCAAAATGAGTGTGATGAACTGTTTGTTCATGGTCGTT

TTTTGAACTCGGATCAATTTGAGCTACCTATCGGCATTAATAAGCAGCGCGATGGCTCGACAGAGGTTAA

AATAGGCGGTCAATCTGGGCAAAAATTGGCGCAATTGGCGCAAGTGTTACCGCTGCAGTTGATACATCCA

>CP176357.1 Vibrio parahaemolyticus strain AG1 chromosome 1, complete sequence

AAAAAATACCCGAAAGGGAAAGGGTGGTTTTCTAGTTCTTTTATTATTAATATCCACATCATTAAGTAGC

AAGTTAAATGCCTTTTCATTTTTGAGAATGACTCTGATATGTAGGTAAATATTAATTTTCAAAATTGAAT

GTTTACTATATACGTTAAGTTGTATTTAATATGTATGTTTTTAGTTAGTTAGAGGTAAGCGCTCACACAT

GTGTATTACTAAATGGAAGGGAAACCTTATAACATTGATAATGATTGTTATTAGTCTTTCTGGTTGGGCT

ACTTCGATATATATGTATAACGATTTAATCGCCTACCAATCTCGAGTTTCTGAAGGTAAGGTCATAAACG

CTTATAATATATTAGTTTCAGCATTTAAACGGTCAGATTCAGAAGACGAGATCTTACTTATAGTGAATGA

TTGGAAAATTAAGGGGAGTAGTGCTGAACTCGGTTCTTTGACAACAATGTGCAATAATAACCCCTCTATT

TTATTAGACGTATCTAGTGCTTTCACTGAAGTTCTAATTCTGAAAATATGTAAAACTATTGAATCTAACA

CGTGATGCTGTTTTTCTTAATTTTGAGAACCGTGTTTGTTTTACCTTGTGTTTTTTATTTCTATTGATAT

CTTACTCACATCTCAAACATTTGGAGTTGGTTAACGAGTTTATTAAATTTCTCTTCAATCTCGATGTAAA

CTTAAGTCGTTGTAATTTATGTTATTTTTTAATTGAAATTATGCAGGTTCTTCGAGTGACGATATTAATG

TGCTTCAAAAGATGTTTGTAAATCCTAAGTTCTTAATTAAATAACTCTCTGTCACGTGTCGATTAAATAT

AACCTTCCGAGTCTATTTTTTTTCTTTCTATGAACTTTAGTTATAAGTCTAAAGTCGTAGAGTTTATTTC

TCAAAGTTTAGATATTGCTCACAATCCGAATTGGGTGAGCCCTTTTCTTTAAGACTAATATATAACTTGT

TTATTTAAATGAATTTTTCTGAAAAAAACATGAGAATGTATATGAAAAACCTTTTTTTAACGTTTGTCGT

TTTGTCGGTCAATTTCAATGTATCTGCTTCTGAGTCAGAAGCTGAGCCAAATTGGTATCTGGGGGCAGGC

CTTGGCGTTGGAGATTATGGTGATGTTAAACACCCTGATGCTTACGACAGTGCAACAGACAGATTAGCTG

GTGAGCTTTATCTTGGCTATCGACTTAATCCTTACCTTTCCTCTGAATTGAGCTACCAATATCTTGGAAG

CGCATATGCTAAATACCCTCAAGGAGAGATCAAAGGAGACTTCCAACAAGTGGTGTTAGCCGCAAGACTT

GGTTATCCGTTAGATTCGGCATTTTATCCCTACGCTAAGTTGGGTGGTGCAGCTTGGTTTGGTAATAGTG

AAGGATTAAGAGAGGCAAATGAACAAGGGTTTGCTCCCGTCATTGGAGCCGGTATCGAATATGCTTTTAC

CGAGTCGTTGGTTGGCCGTGTTGAGTATCAATATACAGATAGCCTTGGTGACGAGGAGGTCGGTTATACC

AATCACCACATGGCAACGTTAGGTCTAGCTTGGCAATTTGGTCATTCCAAACAACCGGTTATCGATGAGC

CGATTGAAGTGCCACCTCAACCAGTTGAGACGCAAACATTTGTTTTTTCTGAGCAGAAACAGAATCACCT

TTTTGCTTTTGATTCCAGTCAACTTAACAACAAGGCTGCGTTTACAGAAGTTCTTTCTTTTCTGGAAAAA

TACCCTCAGGCACAAGTCGTCATTGAAGGGCATACCGACAGTGTTGGCTCAGAAGAATACAACCAAAAAT

TATCATCAAGACGCGCGGAAGCTGTTGCTCAATACTTCATCGAACAAGGTGTAGAGCCTTCAAATATATC

GACCGTTGGAAAAGGGGAAGCATCTCCAATTGCTGACAATACAACAGTGGATGGGCGAGCTATGAATCGC

CGAATTGAAATTAGTATTCCGAGTTTGTCGGTATCAACAAAAGAATAAAAGTAATTAATGGAGTGAGTAT

GTCGTATAGCAACAAGATAGCGCCTTTGACGGCCGCAATACTGGCGACACTAGTCGTAGGTTGCGATGGG

GACAGTAAAGACAGTCAGTTCCTTGAGCCAGACCAACAAGTCAATCGTTTGACCGCCTACCCTCAAGCTT

TTGTTTTAAAAACAGGTGATGTACAAAATGTCGATTTAACTTCCTCAGTTATTGCTGAAAATATAGCAAG

TTGGAAATTGAGTGCACTGGATGATAAAAGCGGGCTGGGTCAAATTACTGCCCAGGCAGAGCAAGCTTTT

GAATACTCGACAGACACTGGAGGTGTTGGGCATATCAATTACACCGTCAGTGGTGATGGTCTAACTTCCA

GCTCTCAAGTTATTTTAGCCGTTAATGACGGTGGGACTCTTGGTAACAAAACTCCTGAGGCTCAAAACGT

TACTTTAAGTACTACCAACACGAGTGATGTGAGTGTTGACTTGCGTGATTATATCTCTGATGAAGATGGA

GACGAATTACAAATTACGTCACTGATTTCTGCATCGAACCGCTTTGCTATCGCAGATGATGGTTACCAAG

TCGTATTTACACCTGATGGTTATGTGGGCATAGATCAGGCTGTTTATAGTGTTGACGATGGTAAAGGGGG

ATATGCACTGGCGTACATTTTAGCGACTGCAGCTGACCCAACTGCACCTAACACGCCTCCAGTCGCGAAA

GATGATAGTCTGACAATGGACGTGGCATCACAATCGGTACTCAATATCGACTTAAATGCACTTATCAGTG

ATGTTGATGGTGATACCTTGATGGTTGAGCAACTCTACAGCGCCAACAATCGAGCTGAGCTTACTAGTTC

AAATAGCGTGCGCTACATTCCAAATGATTTCCGAGGGGTCGACCAAATCACTTATCGAGTCACTGACAAT

AAAGGTGGCGAGGCTTTTGGCACGATAACTATTACCGTTAGTGATTCATCAATACAACCGGAGGTGCCGG

CAATATCGGCTTTCCCTCAGACATTTAATCTTGAGCCGGGGGATTCGATTAACGTCGATGTTACTCAATC

AGTGGTTGCGACGAACATCGACAGTTGGGCATTGGTAAAGGCAGAAGATAGCAGTGGGCTTGGCATGGTT

TCTGGCCTCACAGCCACATCATTTGACTATCTTGCACAAGCGCCGGGTGTTGCAACGATTAATTATGATG

TTCAAGGAGGAGGGGTAAGCGATTCTTCGACAGTGATTGCGTTGATCAATACGCCACCATCACCAGACAA

TACACCGCCAACGGCAAAAAATGTCACGGCAGATACCACCAATGATACGAATGTGAGTGTTGACCTAAGT

AATGAAATTGCTGATGTCGATGGTGATACGCTCACAATCAGTCAATTAGTCTCAGCTTCAGGGCGTTTCA

CACTAACAGGCTCACAAGTTACTTTTAAGCCCGATGGGTTTATTGGGGTTGACCAAGCGGCTTATATTGT

GGAGGACGGTCAAGGTGGTTATGCCTCGGCTTATGTTGTTATCACCTCGACAGATTCGAACCCGACAACA

CCTAATACGCCACCAACGGCAGCAGATTATAGCTACGTAATGGATGTGGCTAGCTTACCCGTTTGGAATT

TTGACTTAGCAGCACTGAATTTGGTCGCTGATGCTGATGGTGATGCTTTAACGATTGAAAACATCTACAG

CGCAGACGGACGTGCGGTTAAGCAAGGTGCAACTGGAGTGACTTACACGCCTGGAGATTTTAGGGGTATC

GATCTTATTACCTACAGTGTCTCAGATGGCAATGGGGGCAGTGCGATAGGTTCGATAACGGTGGTTGTTA

ATGATTCAACTCCTGGAAATACAATCCCTACAGCCAATGCAGTGTCTACGACAATGCTTGATACAGATGC

GAGTAAAACGATCTCCGTAGCGAGCAGTGTATCGGATAGCGATGGTGACGCGCTAGAAATTGTGGAGCTT

CAGGCATCAATTGGTACTGCGAGCATCAATCCGAGCAATCCGTTAGAAGTTATTTACGCGCCTAAAGCGG

GATTTGTAGGTGAGGACCGCTTTGTTTATATCGTCAGCGATGGTAATGGTGGCTTCGCTATGGCGACGAT

TAGTGTGACGGTGAATGCGAGTAATCCAACAGCGCCTGTCGCGAATATTGTTCAAGTGTCGACGACACCG

GATGCTGCAACTGTAATTGATTTATCGGGTTACATCTCCGACAAAGAAACACCAACCGCGAATTTAGTGA

TCAGCAGCGTGAGTACACCGACTTCTCCTGCAACGTTGACACAAAGTGGTCAAAACGTGACTTACACTCC
