# Supplementary material for: Molecular Identification of Culturable Bacteria and Vibrio Virulence‐Related Genes Associated With Sea Turtle Eggshells and Nest Sediments in Pangandaran, Indonesia
Source: Int J Microbiol. 2026 Oct 5;2026:7422673. doi: 10.1155/ijm/7422673 (PMC13639395; doi:10.1155/ijm/7422673)
Supplement: Supplementary file 2 [file IJM-2026-7422673-s002.zip › Hasil NCBI/VS Consensus.docx]

>JN672574.1 Uncultured bacterium clone GBO51O2hO6 16S ribosomal RNA gene, partial sequence

AGCGGCGGACGGGTGAGTAACACGTGGGCAACCTGCCCTTGAGACTGGGATAACTTCGGGAAACCGGAGC

TAATACCGGATAATATAAGGAACCTCCTGGTTCCTTATTGAAAGATGGTTTCGGCTATCACTTAAGGATG

GGCCCGCGGCGCATTAGCTAGTTGGTGAGGTAACGGCTCACCAAGGCGACGATGCGTAGCCGACCTGAGA

GGGTGATCGGCCACACTGGGACTGAGACACGGCCCAGACTCCTACGGGAGGCAGCAGTAGGGAATCTTCC

ACAATGGACGAAAGTCTGATGGAGCAACGCCGCGTGAGTGATGAAGGCCTTCGGGTCGTAAAGCTCTGTT

GTTAGGGAAGAACAAGTGCGAGAGTAACTGCTCGCACCTTGACGGTACCTAACCAGAAAGCCACGGCTAA

CTACGTGCCAGCAGCCGCGGTAATACGTAGGTGGCAAGCGTTATCCGGAATTATTGGGCGTAAAGCGCGC

GCAGGTGGTTTCTTAAGTCTGATGTGAAAGCCCACGGCTCAACCGTGGAGGGTCATTGGAAACTGGGGAA

CTTGAGTGCAGAAGAGGAAAGTGGAATTCCAAGTGTAGCGGTGAAATGCGTAGAGATTTGGAGGAACACC

AGTGGCGAAGGCGACTTTCTGGTCTGTAACTGACACTGAGGCGCGAAAGCGTGGGGAGCAAACAGGATTA

GATACCCTGGTAGTCCACGCCGTAAACGATGAGTGCTAAGTGTTAGAGGGTTTCCGCCCTTTAGTGCTGC

AGCTAACGCATTAAGCACTCCGCCTGGGGAGTACGGTCGCAAGACTGAAACTCAAAGGAATTGACGGGGG

CCCGCACAAGCGGTGGAGCATGTGGTTTAATTCGAAGCAACGCGAAGAACCTTACCAGGTCTTGACATCC

TCTGACAACCCTAGAGATAGGGCGTTCCCCTTCGGGGGACAGAGTGACAGGTGGTGCATGGTTGTCGTCA

GCTCGTGTCGTGAGATGTTGGGTTAAGTCCCGCAACGAGCGCAACCCTTGATCTTAGTTGCCAGCATTAG

GTTGGGCACTCTAAGGTGACTGCCGGTGACAAACCGGAGGAAGGTGGGGATGACGTCAAATCATCATGCC

CCTTATGACCTGGGCTACACACGTGCTACAATGGACGGTACAAAGGGCAGCAAAACCGCGAGGTCGAGCC

AATCCCATAAAACCGTTCTCAGTTCGGATTGCAGGCTGCAACTCGCCTGCATGAAGCCGGAATCGCTAGT

AATCGCGGATCAGCATGCCGCGGTGAATACGTTCCCGGGCCTTGC

>MT829440.1 Bacillus sp. (in: firmicutes) strain DM2-16 16S ribosomal RNA gene, partial sequence

AGTCGAGCGGACTTACAAAAGCTTGCTTTTGTAAGTTAGCGGCGGACGGGTGAGTAACACGTGGGCAACC

TGCCCTTGAGACTGGGATAACTTCGGGAAACCGGAGCTAATACCGGATAATATAAGGAACCTCCTGGTTC

CTTGTTGAAAGATGGTTTCGGCTATCACTTAAGGATGGGCCCGCGGCGCATTAGCTAGTTGGTGAGGTAA

CGGCTCACCAAGGCGACGATGCGTAGCCGACCTGAGAGGGTGATCGGCCACACTGGGACTGAGACACGGC

CCAGACTCCTACGGGAGGCAGCAGTAGGGAATCTTCCACAATGGACGAAAGTCTGATGGAGCAACGCCGC

GTGAGTGATGAAGGCCTTCGGGTCGTAAAGCTCTGTTGTTAGGGAAGAACAAGTGCGAGAGTAACTGCTC

GCACCTTGACGGTACCTAACCAGAAAGCCACGGCTAACTACGTGCCAGCAGCCGCGGTAATACGTAGGTG

GCAAGCGTTATCCGGAATTATTGGGCGTAAAGCGCGCGCAGGTGGTTTCTTAAGTCTGATGTGAAAGCCC

ACGGCTCAACCGTGGAGGGTCATTGGAAACTGGGGAACTTGAGTGCAGAAGAGGAAAGTGGAATTCCAAG

TGTAGCGGTGAAATGCGTAGAGATTTGGAGGAACACCAGTGGCGAAGGCGACTTTCTGGTCTGTAACTGA

CACTGAGGCGCGAAAGCGTGGGGAGCAAACAGGATTAGATACCCTGGTAGTCCACGCCGTAAACGATGAG

TGCTAAGTGTTAGAGGGTTTCCGCCCTTTAGTGCTGCAGCTAACGCATTAAGCACTCCGCCTGGGGAGTA

CGGTCGCAAGACTGAAACTCAAAGGAATTGACGGGGGCCCGCACAAGCGGTGGAGCATGTGGTTTAATTC

GAAGCAACGCGAAGAACCTTACCAGGTCTTGACATCCTCTGACAACCCTAGAGATAGGGCGTTCCCCTTC

GGGGGACAGAGTGACAGGTGGTGCATGGTTGTCGTCAGCTCGTGTCGTGAGATGTTGGGTTAAGTCCCGC

AACGAGCGCAACCCTTGATCTTAGTTGCCAGCATTAGGTTGGGCACTCTAAGGTGACTGCCGGTGACAAA

CCGGAGGAAGGTGGGGATGACGTCAAATCATCATGCCCCTTATGACCTGGGCTACACACGTGCTACAATG

GACGGTACAAAGGGCAGCAAAACCGCGAGGTCGAGCCAATCCCATAAAACCGTTCTCAGTTCGGATTGCA

GGCTGCAACTCGCCTGCATGAAGCCGGAATCGCTAGTAATCGCGGATCAGCATGCCGCGGTGAATACGTT

CCCGGGCCTTGTACACACCGCCCGTCACACCACGAGAGTTTGTAACACCCGAAGTCGGTGGGGTAACCTT

TTGGAGCCAGCCGCCTAA

>OQ186772.1 Sutcliffiella sp. strain 81E02 16S ribosomal RNA gene, partial sequence

AAGCTTGCTTTTGTAAGTTAGCGGCGGACGGGTGAGTAACACGTGGGCAACCTGCCCTTGAGACTGGGAT

AACTTCGGGAAACCGGAGCTAATACCGGATAATATAAGGAACCTCCTGGTTCCTTGTTGAAAGATGGTTT

CGGCTATCACTTAAGGATGGGCCCGCGGCGCATTAGCTAGTTGGTGAGGTAACGGCTCACCAAGGCGACG

ATGCGTAGCCGACCTGAGAGGGTGATCGGCCACACTGGGACTGAGACACGGCCCAGACTCCTACGGGAGG

CAGCAGTAGGGAATCTTCCACAATGGACGAAAGTCTGATGGAGCAACGCCGCGTGAGTGATGAAGGCCTT

CGGGTCGTAAAGCTCTGTTGTTAGGGAAGAACAAGTGCGAGAGTAACTGCTCGCACCTTGACGGTACCTA

ACCAGAAAGCCACGGCTAACTACGTGCCAGCAGCCGCGGTAATACGTAGGTGGCAAGCGTTATCCGGAAT

TATTGGGCGTAAAGCGCGCGCAGGTGGTTTCTTAAGTCTGATGTGAAAGCCCACGGCTCAACCGTGGAGG

GTCATTGGAAACTGGGGAACTTGAGTGCAGAAGAGGAAAGTGGAATTCCAAGTGTAGCGGTGAAATGCGT

AGAGATTTGGAGGAACACCAGTGGCGAAGGCGACTTTCTGGTCTGTAACTGACACTGAGGCGCGAAAGCG

TGGGGAGCAAACAGGATTAGATACCCTGGTAGTCCACGCCGTAAACGATGAGTGCTAAGTGTTAGAGGGT

TTCCGCCCTTTAGTGCTGCAGCTAACGCATTAAGCACTCCGCCTGGGGAGTACGGTCGCAAGACTGAAAC

TCAAAGGAATTGACGGGGGCCCGCACAAGCGGTGGAGCATGTGGTTTAATTCGAAGCAACGCGAAGAACC

TTACCAGGTCTTGACATCCTCTGACAACCCTAGAGATAGGGCGTTCCCCTTCGGGGGACAGAGTGACAGG

TGGTGCATGGTTGTCGTCAGCTCGTGTCGTGAGATGTTGGGTTAAGTCCCGCAACGAGCGCAACCCTTGA

TCTTAGTTGCCAGCATTAGGTTGGGCACTCTAAGGTGACTGCCGGTGACAAACCGGAGGAAGGTGGGGAT

GACGTCAAATCATCATGCCCCTTATGACCTGGGCTACACACGTGCTACAATGGACGGTACAAAGGGCAGC

AAAACCGCGAGGTCGAGCCAATCCCATAAAACCGTTCTCAGTTCGGATTGCAGGCTGCAACTCGCCTGCA

TGAAGCCGGAATCGCTAGTAATCGCGGATCAGCATGCCGCGGTGAATACGTTCCCGGGCCTTGTACACAC

CGCCCGTCACACCACGAGAGTTTGTAACACCCGAAGTCGGTGGGGTAACCTTTGGAGCCAGCCG

>JN600607.1 Uncultured bacterium clone GTM1018h12 16S ribosomal RNA gene, partial sequence

TGCCTAACACATGCAAGTCGAGCGGACTTACAAAAGCTTGCTTTTGTAAGTTAGCGGCGGACGGGTGAGT

AACACGTGGGCAACCTGCCCTTGAGACTGGGATAACTTCGGGAAACCGGAGCTAATACCGGATAATATGA

GGAACCTCCTGGTTCCTTATTGAAAGATGGTTTCGGCTATCACTTAAGGATGGGCCCGCGGCGCATTAGC

TAGTTGGTGAGGTAACGGCTCACCAAGGCGACGATGCGTAGCCGACCTGAGAGGGTGATCGGCCACACTG

GGACTGAGACACGGCCCAGACTCCTACGGGAGGCAGCAGTAGGGAATCTTCCACAATGGACGAAAGTCTG

ATGGAGCAACGCCGCGTGAGTGATGAAGGCCTTCGGGTCGTAAAGCTCTGTTGTTAGGGAAGAACAAGTG

CGAGAGTAACTGCTCGCACCTTGACGGTACCTAACCAGAAAGCCACGGCTAACTACGTGCCAGCAGCCGC

GGTAATACGTAGGTGGCAAGCGTTATCCGGAATTATTGGGCGTAAAGCGCGCGCAGGTGGTTTCTTAAGT

CTGATGTGAAAGCCCACGGCTCAACCGTGGAGGGTCATTGGAAACTGGGGAACTTGAGTGCAGAAGAGGA

AAGTGGAATTCCAAGTGTAGCGGTGAAATGCGTAGAGATTTGGAGGAACACCAGTGGCGAAGGCGACTTT

CTGGTCTGTAACTGACACTGAGGCGCGAAAGCGTGGGGAGCAAACAGGATTAGATACCCTGGTAGTCCAC

GCCGTAAACGATGAGTGCTAAGTGTTAGAGGGTTTCCGCCCTTTAGTGCTGCAGCTAACGCATTAAGCAC

TCCGCCTGGGGAGTACGGTCGCAAGACTGAAACTCAAAGGAATTGACGGGGGCCCGCACAAGCGGTGGAG

CATGTGGTTTAATTCGAAGCAACGCGAAGAACCTTACCAGGTCTTGACATCCTCTGACAACCCTAGAGAT

AGGGCGTTCCCCTTCGGGGGACAGAGTGACAGGTGGTGCATGGTTGTCGTCAGCTCGTGTCGTGAGATGT

TGGGTTAAGTCCCGCAACGAGCGCAACCCTTGATCTTAGTTGCCAGCATTAGGTTGGGCACTCTAAGGTG

ACTGCCGGTGACAAACCGGAGGAAGGTGGGGATGACGTCAAATCATCATGCCCCTTATGACCTGGGCTAC

ACACGTGCTACAATGGACGGTACAAAGGGCAGCAAAACCGCGAGGTCGAGCCAATCCCATAAAACCGTTC

TCAGTTCGGATTGCAGGCTGCAACTCGCCTGCATGAAGCCGGAATCGCTAGTAATCGCGGATCAGCATGC

CGCGGTGAATACGTTCCCGGGCCTTGCACACACCGCCCGTCACA

>PV707101.1 Sutcliffiella sp. strain StRS-1 16S ribosomal RNA gene, partial sequence

CAAAAGCTTGCTTTTGTAAGTTAGCGGCGGACGGGTGAGTAACACGTGGGCAACCTGCCCTTGAGACTGG

GATAACTTCGGGAAACCGGAGCTAATACCGGATAATATAAGGAACCTCCTGGTTCCTTRTTGAAAGATGG

TTTCGGCTATCACTTAAGGATGGGCCCGCGGCGCATTAGCTAGTTGGTGAGGTAACGGCTCACCAAGGCG

ACGATGCGTAGCCGACCTGAGAGGGTGATCGGCCACACTGGGACTGAGACACGGCCCAGACTCCTACGGG

AGGCAGCAGTAGGGAATCTTCCACAATGGACGAAAGTCTGATGGAGCAACGCCGCGTGAGTGATGAAGGC

CTTCGGGTCGTAAAGCTCTGTTGTTAGGGAAGAACAAGTGCGAGAGTAACTGCTCGCACCTTGACGGTAC

CTAACCAGAAAGCCACGGCTAACTACGTGCCAGCAGCCGCGGTAATACGTAGGTGGCAAGCGTTATCCGG

AATTATTGGGCGTAAAGCGCGCGCAGGTGGTTTCTTAAGTCTGATGTGAAAGCCCACGGCTCAACCGTGG

AGGGTCATTGGAAACTGGGGAACTTGAGTGCAGAAGAGGAAAGTGGAATTCCAAGTGTAGCGGTGAAATG

CGTAGAGATTTGGAGGAACACCAGTGGCGAAGGCGACTTTCTGGTCTGTAACTGACACTGAGGCGCGAAA

GCGTGGGGAGCAAACAGGATTAGATACCCTGGTAGTCCACGCCGTAAACGATGAGTGCTAAGTGTTAGAG

GGTTTCCGCCCTTTAGTGCTGCAGCTAACGCATTAAGCACTCCGCCTGGGGAGTACGGTCGCAAGACTGA

AACTCAAAGGAATTGACGGGGGCCCGCACAAGCGGTGGAGCATGTGGTTTAATTCGAAGCAACGCGAAGA

ACCTTACCAGGTCTTGACATCCTCTGACAACCCTAGAGATAGGGCGTTCCCCTTCGGGGGACAGAGTGAC

AGGTGGTGCATGGTTGTCGTCAGCTCGTGTCGTGAGATGTTGGGTTAAGTCCCGCAACGAGCGCAACCCT

TGATCTTAGTTGCCAGCATTAGGTTGGGCACTCTAAGGTGACTGCCGGTGACAAACCGGAGGAAGGTGGG

GATGACGTCAAATCATCATGCCCCTTATGACCTGGGCTACACACGTGCTACAATGGACGGTACAAAGGGC

AGCAAAACCGCGAGGTCGAGCCAATCCCATAAAACCGTTCTCAGTTCGGATTGCAGGCTGCAACTCGCCT

GCATGAAGCCGGAATCGCTAGTAATCGCGGATCAGCATGCCGCGGTGAATACGTTCCCGGGCCTTGTACA

CACCGCCCGTCACACCACGAGAGTTTGTAACACCCGAAGTCGGTGGGGTAAC

>HM846047.1 Uncultured bacterium clone nby662a08c1 16S ribosomal RNA gene, partial sequence

GATGAACGCTGGCGGCGTGCCTAATACATGCAAGTCGAGCGGACTTACAAAAGCTTGCTTTTGTAAGTTA

GCGGCGGACGGGTGAGTAACACGTGGGCAACCTGCCCTTGAGACTGGGATAACTTCGGGAAACCGGAGCT

AATACCGGATAATATAAGGAACCTCCTGGTTCCTTGTTGAAAGATGGTTTCGGCTATCACTTAAGGATGG

GCCCGCGGCGCATTAGCTAGTTGGTGAGGTAACGGCTCACCAAGGCGACGATGCGTAGCCGACCTGAGAG

GGTGATCGGCCACACTGGGACTGAGACACGGCCCAGACTCCTACGGGAGGCAGCAGTAGGGAATCTTCCA

CAATGGACGAAAGTCTGATGGAGCAACGCCGCGTGAGTGATGAAGGCCTTCGGGTCGTAAAGCTCTGTTG

TTAGGGAAGAACAAGTGCGAGAGTAACTGCTCGCACCTTGACGGTACCTAACCAGAAAGCCACGGCTAAC

TACGTGCCAGCAGCCGCGGTAATACGTAGGTGGCAAGCGTTATCCGGAATTATTGGGCGTAAAGCGCGCG

CAGGTGGTTTCTTAAGTCTGATGTGAAAGCCCACGGCTCAACCGTGGAGGGTCATTGGAAACTGGGGAAC

TTGAGTGCAGAAGAGGAAAGTGGAATTCCAAGTGTAGCGGTGAAATGCGTAGAGATTTGGAGGAACACCA

GTGGCGAAGGCGACTTTCTGGTCTGTAACTGACACTGAGGCGCGAAAGCGTGGGGAGCAAACAGGATTAG

ATACCCTGGTAGTCCACGCCGTAAACGATGAGTGCTAAGTGTTAGAGGGTTTCCGCCCTTTAGTGCTGCA

GCTAACGCATTAAGCACTCCGCCTGGGGAGTACGGTCGCAAGACTGAAACTCAAAGGAATTGACGGGGGC

CCGCACAAGCGGTGGAGCATGTGGTTTAATTCGAAGCAACGCGAAGAACCTTACCAGGTCTTGACATCCT

CTGACAACCCTAGAGATAGGGCGTTCCCCTTCGGGGGACAGAGTGACAGGTGGTGCATGGTTGTCGTCAG

CTCGTGTCGTGAGATGTTGGGTTAAGTCCCGCAACGAGCGCAACCCTTGATCTTAGTTGCCAGCATTAGG

TTGGGCACTCTAAGGTGACTGCCGGTGACAAACCGGAGGAAGGTGGGGATGACGTCAAATCATCATGCCC

CTTATGACCTGGGCTACACACGTGCTACAATGGACGGTACAAAGGGCAGCAAAACCGCGAGGTCGAGCCA

ATCCCATAAAACCGTTCTCAGTTCGGATTGCAGGCTGCAACTCGCCTGCATGAAGCCGGAATCGCTAGTA

ATCGCGGATCAGCATGCCGCGGTGAATACGTTCCCGGGCCT
