# Supplementary material for: Molecular Identification of Culturable Bacteria and Vibrio Virulence‐Related Genes Associated With Sea Turtle Eggshells and Nest Sediments in Pangandaran, Indonesia
Source: Int J Microbiol. 2026 Oct 5;2026:7422673. doi: 10.1155/ijm/7422673 (PMC13639395; doi:10.1155/ijm/7422673)
Supplement: Supplementary file 2 [file IJM-2026-7422673-s002.zip › Hasil NCBI/RS Consensus.docx]

>PP725227.1 Pseudomonas fragi strain J5 16S ribosomal RNA gene, partial sequence

GGGCCCGTGCCGGCAGCTAACACATGCAGTCGAGCGGTAGAGAGAAGCTTGCTTCTCTTGAGAGCGGCGG

ACGGGTGAGTAATACCTAGGAATCTGCCTGATAGTGGGGGATAACGTTCGGAAACGGACGCTAATACCGC

ATACGTCCTACGGGAGAAAGCAGGGGACCTTCGGGCCTTGCGCTATCAGATGAGCCTAGGTCGGATTAGC

TAGTTGGTGAGGTAATGGCTCACCAAGGCTACGATCCGTAACTGGTCTGAGAGGATGATCAGTCACACTG

GAACTGAGACACGGTCCAGACTCCTACGGGAGGCAGCAGTGGGGAATATTGGACAATGGGCGAAAGCCTG

ATCCAGCCATGCCGCGTGTGTGAAGAAGGTCTTCGGATTGTAAAGCACTTTAAGTTGGGAGGAAGGGCAT

TAACCTAATACGTTGGTGTCTTGACGTTACCGACAGAATAAGCACCGGCTAACTCTGTGCCAGCAGCCGC

GGTAATACAGAGGGTGCAAGCGTTAATCGGAATTACTGGGCGTAAAGCGCGCGTAGGTGGTTTGTTAAGT

TGAATGTGAAATCCCCGGGCTCAACCTGGGAACTGCATCCAAAACTGGCAAGCTAGAGTATGGTAGAGGG

TAGTGGAATTTCCTGTGTAGCGGTGAAATGCGTAGATATAGGAAGGAACACCAGTGGCGAAGGCGACTAC

CTGGACTGATACTGACACTGAGGTGCGAAAGCGTGGGGAGCAAACAGGATTAGATACCCTGGTAGTCCAC

GCCGTAAACGATGTCAACTAGCCGTTGGGAGTCTTGAACTCTTAGTGGCGCAGCTAACGCATTAAGTTGA

CCGCCTGGGGAGTACGGCCGCAAGGTTAAAACTCAAATGAATTGACGGGGGCCCGCACAAGCGGTGGAGC

ATGTGGTTTAATTCGAAGCAACGCGAAGAACCTTACCAGGCCTTGACATCCAATGAACTTTCCAGAGATA

GATTGGTGCCTTCGGGAACATTGAGACAGGTGCTGCATGGCTGTCGTCAGCTCGTGTCGTGAGATGTTGG

GTTAAGTCCCGTAACGAGCGCAACCCTTGTCCTTAGTTACCAGCACGTAATGGTGGGCACTCTAAGGAGA

CTGCCGGTGACAAACCGGAGGAAGGTGGGGATGACGTCAAGTCATCATGGCCCTTACGGCCTGGGCTACA

CACGTGCTACAATGGTCGGTACAAAGGGTTGCCAAGCCGCGAGGTGGAGCTAATCCCATAAAACCGATCG

TAGTCCGGATCGCAGTCTGCAACTCGACTGCGTGAAGTCGGAATCGCTAGTAATCGTGAATCAGAATGTC

ACGGTGAATACGTTCCCGGGCCTTGTACACACCGCCCGTCACACCATGGGAGTGGGTTGCACCAGAAGTA

GCTAGTCTAACCTTCGGGAGGACGGTACCACGGTGGATAAGGGGCTC

>JX867753.1 Pseudomonas fragi strain 2-2 16S ribosomal RNA gene, partial sequence

GGCAGGGCAGCGGGTCTACTCTGCAGTCGAGCGGTAGAGAGAAGCTTGCTTCTCTTGAGAGCGGCGGACG

GGTGAGTAATACCTAGGAATCTGCCTGATAGTGGGGGATAACGTTCGGAAACGGACGCTAATACCGCATA

CGTCCTACGGGAGAAAGCAGGGGACCTTCGGGCCTTGCGCTATCAGATGAGCCTAGGTCGGATTAGCTAG

TTGGTGAGGTAATGGCTCACCAAGGCTACGATCCGTAACTGGTCTGAGAGGATGATCAGTCACACTGGAA

CTGAGACACGGTCCAGACTCCTACGGGAGGCAGCAGTGGGGAATATTGGACAATGGGCGAAAGCCTGATC

CAGCCATGCCGCGTGTGTGAAGAAGGTCTTCGGATTGTAAAGCACTTTAAGTTGGGAGGAAGGGCATTAA

CCTAATACGTTGGTGTCTTGACGTTACCGACAGAATAAGCACCGGCTAACTCTGTGCCAGCAGCCGCGGT

AATACAGAGGGTGCAAGCGTTAATCGGAATTACTGGGCGTAAAGCGCGCGTAGGTGGTTTGTTAAGTTGA

ATGTGAAATCCCCGGGCTCAACCTGGGAACTGCATCCAAAACTGGCAAGCTAGAGTATGGTAGAGGGTAG

TGGAATTTCCTGTGTAGCGGTGAAATGCGTAGATATAGGAAGGAACACCAGTGGCGAAGGCGACTACCTG

GACTGATACTGACACTGAGGTGCGAAAGCGTGGGGAGCAAACAGGATTAGATACCCTGGTAGTCCACGCC

GTAAACGATGTCAACTAGCCGTTGGGAGTCTTGAACTCTTAGTGGCGCAGCTAACGCATTAAGTTGACCG

CCTGGGGAGTACGGCCGCAAGGTTAAAACTCAAATGAATTGACGGGGGCCCGCACAAGCGGTGGAGCATG

TGGTTTAATTCGAAGCAACGCGAAGAACCTTACCAGGCCTTGACATCCAATGAACTTTCCAGAGATAGAT

TGGTGCCTTCGGGAACATTGAGACAGGTGCTGCATGGCTGTCGTCAGCTCGTGTCGTGAGATGTTGGGTT

AAGTCCCGTAACGAGCGCAACCCTTGTCCTTAGTTACCAGCACGTAATGGTGGGCACTCTAAGGAGACTG

CCGGTGACAAACCGGAGGAAGGTGGGGATGACGTCAAGTCATCATGGCCCTTACGGCCTGGGCTACACAC

GTGCTACAATGGTCGGTACAAAGGGTTGCCAAGCCGCGAGGTGGAGCTAATCCCATAAAACCGATCGTAG

TCCGGATCGCAGTCTGCAACTCGACTGCGTGAAGTCGGAATCGCTAGTAATCGTGAATCAGAATGTCACG

GTGAATACGTTCCCGGGCCTTGTACACACCGCCCGTCACACCATGGGAGTGGGTTGCACCAGAAGTAGCT

AGTCTAACCTTCGGGAGGACGGTACCAACGGTTAATAAACGGGT

>OM070140.1 Uncultured bacterium clone Y13.2 16S ribosomal RNA gene, partial sequence

GTCGAGCGGTAGAGAGAAGCTTGCTTCTCTTGAGAGCGGCGGACGGGTGAGTAATACCTAGGAATCTGCC

TGATAGTGGGGGATAACGTTCGGAAACGGACGCTAATACCGCATACGTCCTACGGGAGAAAGCAGGGGAC

CTTCGGGCCTTGCGCTATCAGATGAGCCTAGGTCGGATTAGCTAGTTGGTGAGGTAATGGCTCACCAAGG

CTACGATCCGTAACTGGTCTGAGAGGATGATCAGTCACACTGGAACTGAGACACGGTCCAGACTCCTACG

GGAGGCAGCAGTGGGGAATATTGGACAATGGGCGAAAGCCTGATCCAGCCATGCCGCGTGTGTGAAGAAG

GTCTTCGGATTGTAAAGCACTTTAAGTTGGGAGGAAGGGCATTAACCTAATACGTTGGTGTCTTGACGTT

ACCGACAGAATAAGCACCGGCTAACTCTGTGCCAGCAGCCGCGGTAATACAGAGGGTGCAAGCGTTAATC

GGAATTACTGGGCGTAAAGCGCGCGTAGGTGGTTTGTTAAGTTGAATGTGAAATCCCCGGGCTCAACCTG

GGAACTGCATCCAAAACTGGCAAGCTAGAGTATGGTAGAGGGTAGTGGAATTTCCTGTGTAGCGGTGAAA

TGCGTAGATATAGGAAGGAACACCAGTGGCGAAGGCGACTACCTGGACTGATACTGACACTGAGGTGCGA

AAGCGTGGGGAGCAAACAGGATTAGATACCCTGGTAGTCCACGCCGTAAACGATGTCAACTAGCCGTTGG

GAGTCTTGAACTCTTAGTGGCGCAGCTAACGCATTAAGTTGACCGCCTGGGGAGTACGGCCGCAAGGGTA

AAACTCAAATGAATTGACGGGGGCCCGCACAAGCGGTGGAGCATGTGGTTTAATTCGAAGCAACGCGAAG

AACCTTACCAGGCCTTGACATCCAATGAACTTTCCAGAGATAGATTGGTGCCTTCGGGAACATTGAGACA

GGTGCTGCATGGCTGTCGTCAGCTCGTGTCGTGAGATGTTGGGTTAAGTCCCGTAACGAGCGCAACCCTT

GTCCTTAGTTACCAGCACGTAATGGTGGGCACTCTAAGGAGACTGCCGGTGACAAACCGGAGGAAGGTGG

GGATGACGTCAAGTCATCATGGCCCTTACGGCCTGGGCTACACACGTGCTACAATGGTCGGTACAAAGGG

TTGCCAAGCCGCGAGGTGGAGCTAATCCCATAAAACCGATCGTAGTCCGGATCGCAGTCTGCAACTCGAC

TGCGTGAAGTCGGAATCGCTAGTAATCGTGAATCAGAATGTCACGGTGAATACGTTCCCGGGCCTTGTAC

ACACCGCCCGTCACACCATGGGAGTGGGTTGCACCAGAAGTAGCTAGTCTAACCTTCGGGAGGACGGT

>PP725234.1 Pseudomonas fragi strain J12 16S ribosomal RNA gene, partial sequence

AGGGCTCTTGCCGGCCAGCCCTAACACATGCAAGTCGAGCGGTAGAGAGAAGCTTGCTTCTCTTGAGAGC

GGCGGACGGGTGAGTAATACCTAGGAATCTGCCTGATAGTGGGGGATAACGTTCGGAAACGGACGCTAAT

ACCGCATACGTCCTACGGGAGAAAGCAGGGGACCTTCGGGCCTTGCGCTATCAGATGAGCCTAGGTCGGA

TTAGCTAGTTGGTGAGGTAATGGCTCACCAAGGCTACGATCCGTAACTGGTCTGAGAGGATGATCAGTCA

CACTGGAACTGAGACACGGTCCAGACTCCTACGGGAGGCAGCAGTGGGGAATATTGGACAATGGGCGAAA

GCCTGATCCAGCCATGCCGCGTGTGTGAAGAAGGTCTTCGGATTGTAAAGCACTTTAAGTTGGGAGGAAG

GGCATTAACCTAATACGTTGGTGTCTTGACGTTACCGACAGAATAAGCACCGGCTAACTCTGTGCCAGCA

GCCGCGGTAATACAGAGGGTGCAAGCGTTAATCGGAATTACTGGGCGTAAAGCGCGCGTAGGTGGTTTGT

TAAGTTGAATGTGAAATCCCCGGGCTCAACCTGGGAACTGCATCCAAAACTGGCAAGCTAGAGTATGGTA

GAGGGTAGTGGAATTTCCTGTGTAGCGGTGAAATGCGTAGATATAGGAAGGAACACCAGTGGCGAAGGCG

ACTACCTGGACTGATACTGACACTGAGGTGCGAAAGCGTGGGGAGCAAACAGGATTAGATACCCTGGTAG

TCCACGCCGTAAACGATGTCAACTAGCCGTTGGGAGTCTTGAACTCTTAGTGGCGCAGCTAACGCATTAA

GTTGACCGCCTGGGGAGTACGGCCGCAAGGTTAAAACTCAAATGAATTGACGGGGGCCCGCACAAGCGGT

GGAGCATGTGGTTTAATTCGAAGCAACGCGAAGAACCTTACCAGGCCTTGACATCCAATGAACTTTCCAG

AGATAGATTGGTGCCTTCGGGAACATTGAGACAGGTGCTGCATGGCTGTCGTCAGCTCGTGTCGTGAGAT

GTTGGGTTAAGTCCCGTAACGAGCGCAACCCTTGTCCTTAGTTACCAGCACGTAATGGTGGGCACTCTAA

GGAGACTGCCGGTGACAAACCGGAGGAAGGTGGGGATGACGTCAAGTCATCATGGCCCTTACGGCCTGGG

CTACACACGTGCTACAATGGTCGGTACAAAGGGTTGCCAAGCCGCGAGGTGGAGCTAATCCCATAAAACC

GATCGTAGTCCGGATCGCAGTCTGCAACTCGACTGCGTGAAGTCGGAATCGCTAGTAATCGTGAATCAGA

ATGTCACGGTGAATACGTTCCCGGGCCTTGTACACACCGCCCGTCACACCATGGGAGTGGGTTGCACCAG

AAGTAGCTAGTCTAACCTTCGGGAGGACGGTTACCACGGTGGATTAAATTGGGT

>PP725225.1 Pseudomonas fragi strain J3 16S ribosomal RNA gene, partial sequence

CGGGTCTGGCGGCAGGCTACACATGCAAGTCGAGCGGTAGAGAGAAGCTTGCTTCTCTTGAGAGCGGCGG

ACGGGTGAGTAATACCTAGGAATCTGCCTGATAGTGGGGGATAACGTTCGGAAACGGACGCTAATACCGC

ATACGTCCTACGGGAGAAAGCAGGGGACCTTCGGGCCTTGCGCTATCAGATGAGCCTAGGTCGGATTAGC

TAGTTGGTGAGGTAATGGCTCACCAAGGCTACGATCCGTAACTGGTCTGAGAGGATGATCAGTCACACTG

GAACTGAGACACGGTCCAGACTCCTACGGGAGGCAGCAGTGGGGAATATTGGACAATGGGCGAAAGCCTG

ATCCAGCCATGCCGCGTGTGTGAAGAAGGTCTTCGGATTGTAAAGCACTTTAAGTTGGGAGGAAGGGCAT

TAACCTAATACGTTGGTGTCTTGACGTTACCGACAGAATAAGCACCGGCTAACTCTGTGCCAGCAGCCGC

GGTAATACAGAGGGTGCAAGCGTTAATCGGAATTACTGGGCGTAAAGCGCGCGTAGGTGGTTTGTTAAGT

TGAATGTGAAATCCCCGGGCTCAACCTGGGAACTGCATCCAAAACTGGCAAGCTAGAGTATGGTAGAGGG

TAGTGGAATTTCCTGTGTAGCGGTGAAATGCGTAGATATAGGAAGGAACACCAGTGGCGAAGGCGACTAC

CTGGACTGATACTGACACTGAGGTGCGAAAGCGTGGGGAGCAAACAGGATTAGATACCCTGGTAGTCCAC

GCCGTAAACGATGTCAACTAGCCGTTGGGAGTCTTGAACTCTTAGTGGCGCAGCTAACGCATTAAGTTGA

CCGCCTGGGGAGTACGGCCGCAAGGTTAAAACTCAAATGAATTGACGGGGGCCCGCACAAGCGGTGGAGC

ATGTGGTTTAATTCGAAGCAACGCGAAGAACCTTACCAGGCCTTGACATCCAATGAACTTTCCAGAGATA

GATTGGTGCCTTCGGGAACATTGAGACAGGTGCTGCATGGCTGTCGTCAGCTCGTGTCGTGAGATGTTGG

GTTAAGTCCCGTAACGAGCGCAACCCTTGTCCTTAGTTACCAGCACGTAATGGTGGGCACTCTAAGGAGA

CTGCCGGTGACAAACCGGAGGAAGGTGGGGATGACGTCAAGTCATCATGGCCCTTACGGCCTGGGCTACA

CACGTGCTACAATGGTCGGTACAAAGGGTTGCCAAGCCGCGAGGTGGAGCTAATCCCATAAAACCGATCG

TAGTCCGGATCGCAGTCTGCAACTCGACTGCGTGAAGTCGGAATCGCTAGTAATCGTGAATCAGAATGTC

ACGGTGAATACGTTCCCGGGCCTTGTACACACCGCCCGTCACACCATGGGAGTGGGTTGCACCAGAAGTA

GCTAGTCTAACCTTCGGGAGGACGGTACCACGGTGGATCAGTTGCCT
