# Supplementary material for: Molecular Identification of Culturable Bacteria and Vibrio Virulence‐Related Genes Associated With Sea Turtle Eggshells and Nest Sediments in Pangandaran, Indonesia
Source: Int J Microbiol. 2026 Oct 5;2026:7422673. doi: 10.1155/ijm/7422673 (PMC13639395; doi:10.1155/ijm/7422673)
Supplement: Supplementary file 2 [file IJM-2026-7422673-s002.zip › Hasil NCBI/TS Consensus.docx]

>MF537181.1 Bacillus sp. (in: firmicutes) strain CJKOP-136 16S ribosomal RNA gene, partial sequence

GGCCGGACGGCGTGCTATACATGCAAGTCGAGCGGATTGATGGGAGCTTGCTCCCTGATATCAGCGGCGG

ACGGGTGAGTAACACGTGGGTAACCTGCCTGTAAGACTGGGATAACTCCGGGAAACCGGGGCTAATACCG

GATAACTCATTTCCTCGCATGAGGAAATGTTGAAAGATGGCTTCTTGCTATCACTTACAGATGGACCCGC

GGCGCATTAGCTAGTTGGTGAGGTAACGGCTCACCAAGGCAACGATGCGTAGCCGACCTGAAAGGGTGAT

CGGCCACACTGGGACTGAAACACGGCCCAAACTCCTACGGGAGGCAGCAGTAGGGAATCTTCCGCAATGG

ACGAAAGTCTGACGGAGCAACGCCGCGTGAGTGATGAAGGTTTTCGGATCGTAAAACTCTGTTGTTAGGG

AAAAACAAGTACCGTTCGAATAGGGCGGTACCTTGACGGTACCTAACCAGAAAGCCACGGCTAACTACGT

GCCAGCAGCCGCGGTAATACGTAGGTGGCAAGCGTTGTCCGGAATTATTGGGCGTAAAGCGCGCGCAGGT

GGTTTCTTAAGTCTGATGTGAAAGCCCACGGCTCAACCGTGGAGGGTCATTGGAAACTGGGGAACTTGAG

TGCAGAARAGGAAAGTGGAATTCCAAGTGTAGCGGTGAAATGCGTAGATATTTGGAGGAACACCAGTGGC

GAAGGCGACTTTCTGGTCTGTAACTGACACTGAGGCGCGAAAGCGTGGGGAGCAAACAGGATTAGATACC

CTGGTAGTCCACGCCGTAAACGATGAGTGCTAAGTGTTAGRGGGTTTCCGCCCCTTAGTGCTGCAGCTAA

CGCATTAAGCACTCCGCCTGGGGAGTACGGTCGCAAGACTGAAACTCAAAGGAATTGACGGGGGCCCGCA

CAAGCGGTGGAGCATGTGGTTTAATTCGAAGCAACGCGAAGAACCTTACCAGGTCTTGACATCCTCTGAC

AACCCTAGAGATAGGGCTTTCCCCTTCGGGGGACAGAGTGACAGGTGGTGCATGGTTGTCGTCAGCTCGT

GTCGTGAGATGTTGGGTTAAGTCCCGCAACGAGCGCAACCCTTGATCTTAGTTGCCAGCATTCAGTTGGG

CACTCTAAGATGACTGCCGGTGACAAACCGGAGGAAGGTGGGGATGACGTCAAATCATCATGCCCCTTAT

GACCTGGGCTACACACGTGCTACAATGGACGGTACAAAGGGCAGCAAGACCGCGAGGTTTAGCCAATCCC

ATAAAACCGTTCTCAGTTCGGATTGTAGGCTGCAACTCGCCTACATGAAGCTGGAATCGCTAGTAATCGC

GGATCAGCATGCCGCGGTGAATACGTTCCCGGGCCTTGTACACACCGCCCGTCACACCACGAGAGTTTGT

AACACCCGAAGTCGGTGAGGTAACCTTTTGGAGCCAGCCGCCTAAGGGGGACCAGATGTGG

>HQ433449.1 Bacillus sp. A140(2010) 16S ribosomal RNA gene, partial sequence

TAGAGTTTATCCTGGCTCAGGACGAACGCTGGCGGCGTGCCTAATACATGCAAGTCGAGCGGATTGATGG

GAGCTTGCTCCCTGATATCAGCGGCGGACGGGTGAGTAACACGTGGGTAACCTGCCTGTAAGACTGGGAT

AACTCCGGGAAACCGGGGCTAATACCGGATAACTCATTTCCTCGCATGAGGAAATGTTGAAAGATGGCTT

CTTGCTATCACTTACAGATGGACCCGCGGCGCATTAGCTAGTTGGTGAGGTAACGGCTCACCAAGGCAAC

GATGCGTAGCCGACCTGAGAGGGTGATCGGCCACACTGGGACTGAAACACGGCCCAAACTCCTACGGGAG

GCAGCAGTAGGGAATCTTCCGCAATGGACAAAAGTCTGACGGAGCAACGCCGCGTGAGTGATGAAGGTTT

TCGGATCGTAAAACTCTGTTGTTAGGGAAGAACAAGTACCGTTCGAATAGGGCGGTACCTTGACGGTACC

TAACCAGAAAGCCACGGCTAACTACGTGCCAGCAGCCGCGGTAATACGTAGGTGGCAAGCGTTGTCCGGA

ATTATTGGGCGTAAAGCGCGCGCAGGTGGTTTCTTAAGTCTGATGTGAAAGCCCACGGCTCAACCGTGGA

GGGTCATTGGAAACTGGGGAACTTGAGTGCAGAAGAGGAAAGTGGAATTCCAAGTGTAGCGGTGAAAATG

CGTAGATATTTGGAGGAACACCAGTGGCGAAGGCGACTTTCTGGTCTGTAACTGACACTGAGGCGCGAAA

GCGTGGGGAGCAAACAGGATTAGATACCCTGGTAGTCCACGCCGTAAACGATGAGTGCTAAGTGTTAGGG

GGTTTCCGCCCCTTAGTGCTGCAGCTAACGCATTAAGCACTCCGCCTGGGGAGTACGGTCGCAAGACTGA

AACTCAAAGGAATTTGACGGGGGCCCGCACAAGCGGTGGAGCATGTGGTTTAATTCGAAGCAACGCGAAG

AACCTTACCAGGTCTTGACATCCTCTGACAACCCTAGAGATAGGGCTTTCCCCTTCGGGGGACAGAGTGA

CAGGTGGTGCATGGTTGTCGTCAGCTCGTGTCGTGAGAATGTTGGGTTAAGTCCCGCAACGAGGCGCAAC

CCTTGATCTTAGTTGCCAGCATTCAGTTGGGCACTCTAAGATGACTGCCGGTGACAAACCGGAGGAAGGT

GGGGATGACGTCAAATCATCATGCCCCTTATGACCTGGGCTACACACGTGCTACAATGGACGGTACAAAG

GGCAGCAAGACCGCGAGGTTTAGCCAATCCCATAAAACCGTTCTCAGTTCGGATTGTAGGCTGCAACTCG

CCTACATGAAGCTGGAATCGCTAGTAATCGCGGATCAGCATGCCGCGGTGAATACGTTCCCGGGCCTTGT

ACACACCGCCCGTCACACCACGAGAGTTTGTAACACCCGAAGTCGGTGAGGTAACCTTTGGAGCCAGCCG

CCTAAGGTGGACA

>KX350006.1 Rossellomorea aquimaris strain F2-1-19 16S ribosomal RNA gene, partial sequence

TAGAGTTTGATCATGGCTCAGGACGAACGCTGGCGGCGTGCCTAATACATGCAAGTCGAGCGGATTGATG

GGAGCTTGCTCCCTGATATCAGCGGCGGACGGGTGAGTAACACGTGGGTAACCTGCCTGTAAGACTGGGA

TAACTCCGGGAAACCGGGGCTAATACCGGATAACTCATTTCCTCGCATGAGGAAATGTTGAAAGATGGCT

TCTTGCTATCACTTACAGATGGACCCGCGGCGCATTAGCTAGTTGGTGAGGTAACGGCTCACCAAGGCAA

CGATGCGTAGCCGACCTGAGAGGGTGATCGGCCACACTGGGACTGAGACACGGCCCAGACTCCTACGGGA

GGCAGCAGTAGGGAATCTTCCGCAATGGACGAAAGTCTGACGGAGCAACGCCGCGTGAGTGATGAAGGTT

TTCGGATCGTAAAACTCTGTTGTTAGGGAAGAACAAGTACCGTTCGAATAGGGCGGTACCTTGACGGTAC

CTAACCAGAAAGCCACGGCTAACTACGTGCCAGCAGCCGCGGTAATACGTAGGTGGCAAGCGTTGTCCGG

AATTATTGGGCGTAAAGCGCGCGCAGGTGGTTTCTTAAGTCTGATGTGAAAGCCCACGGCTCAACCGTGG

AGGGTCATTGGAAACTGGGGAACTTGAGTGCAGAAGAGGAAAGTGGAATTCCAAGTGTAGCGGTGAAATG

CGTAGATATTTGGAGGAACACCAGTGGCGAAAGGCGACTTTCTGGTCTGTAACTGACACTGAGGCGCGAA

AGCGTGGGGAGCAAACAGGATTAGATACCCTGGTAGTCCACGCCGTAAACGATGAGTGCTAAGTGTTAGG

GGGTTTCCGCCCCTTAGTGCTGCAGCTAACGCATTAAGCACTCCGCCTGGGGAGTACGGTCGCAAGACTG

AAACTCAAAGGAATTGACGGGGGCCCGCACAAGCGGTGGAGCATGTGGTTTAATTCGAAGCAACGCGAAG

AACCTTACCAGGTCTTGACATCCTCTGACAACCCTAGAGATAGGGCTTTCCCCTTCGGGGGACAGAGTGA

CAGGTGGTGCATGGTTGTCGTCAGCTCGTGTCGTGAGATGTTGGGTTAAGTCCCGCAACGAGCGCAACCC

TTGATCTTAGTTGCCAGCATTCAGTTGGGCACTCTAAGATGACTGCCGGTGACAAACCGGAGGAAGGTGG

GGATGACGTCAAATCATCATGCCCCTTATGACCTGGGCTACACACGTGCTACAATGGACGGTACAAAGGG

CAGCAAGACCGCGAGGTTTAGCCAATCCCATAAAACCGTTCTCAGTTCGGATTGTAGGCTGCAACTCGCC

TACATGAAGCTGGAATCGCTAGTAATCGCGGATCAGCATGCCGCGGTGAATACGTTCCCGGGCCTTGTAC

ACACCGCCCGTCACACCACGAGAGTTTGTAACACCCGAAGTCGGTGAGGTAACCTTTTGGAGCCAGCCGC

CTAAGGTGGGACAGATGATTGGGGTGAAGTCGTAACAAGGTAACCA

>JN208048.1 Bacillus aquimaris strain CCMM B683 16S ribosomal RNA gene, partial sequence

TGCTCCCTGATATCAGCGGCGGACGGGTGAGTAACACGTGGGTAACCTGCCTGTAAGACTGGGATAACTC

CGGGAAACCGGGGCTAATACCGGATAACTCATTTCCTCGCATGAGGAAATGTTGAAAGATGGCTTCTTGC

TATCACTTACAGATGGACCCGCGGCGCATTAGCTAGTTGGTGAGGTAACGGCTCACCAAGGCAACGATGC

GTAGCCGACCTGAGAGGGTGATCGGCCACACTGGGACTGAGACACGGCCCAGACTCCTACGGGAGGCAGC

AGTAGGGAATCTTCCGCAATGGACGAAAGTCTGACGGAGCAACGCCGCGTGAGTGATGAAGGTTTTCGGA

TCGTAAAACTCTGTTGTTAGGGAAGAACAAGTACCGTTCGAATAGGGCGGTACCTTGACGGTACCTAACC

AGAAAGCCACGGCTAACTACGTGCCAGCAGCCGCGGTAATACGTAGGTGGCAAGCGTTGTCCGGAATTAT

TGGGCGTAAAGCGCGCGCAGGTGGTTTCTTAAGTCTGATGTGAAAGCCCACGGCTCAACCGTGGAGGGTC

ATTGGAAACTGGGGAACTTGAGTGCAGAAGAGGAAAGTGGAATTCCAAGTGTAGCGGTGAAATGCGTAGA

TATTTGGAGGAACACCAGTGGCGAAGGCGACTTTCTGGTCTGTAACTGACACTGAGGCGCGAAAGCGTGG

GGAGCAAACAGGATTAGATACCCTGGTAGTCCACGCCGTAAACGATGAGTGCTAAGTGTTAGGGGGTTTC

CGCCCCTTAGTGCTGCAGCTAACGCATTAAGCACTCCGCCTGGGGAGTACGGTCGCAAGACTGAAACTCA

AAGGAATTGACGGGGGCCCGCACAAGCGGTGGAGCATGTGGTTTAATTCGAAGCAACGCGAAGAACCTTA

CCAGGTCTTGACATCCTCTGACAACCCTAGAGATAGGGCTTTCCCCTTCGGGGGACAGAGTGACAGGTGG

TGCATGGTTGTCGTCAGCTCGTGTCGTGAGATGTTGGGTTAAGTCCCGCAACGAGCGCAACCCTTGATCT

TAGTTGCCAGCATTCAGTTGGGCACTCTAAGATGACTGCCGGTGACAAACCGGAGGAAGGTGGGGATGAC

GTCAAATCATCATGCCCCTTATGACCTGGGCTACACACGTGCTACAATGGACGGTACAAAGGGCAGCAAG

ACCGCGAGGTTTAGCCAATCCCATAAAACCGTTCTCAGTTCGGATTGTAGGCTGCAACTCGCCTACATGA

AGCTGGAATCGCTAGTAATCGCGGATCAGCATGCCGCGGTGAATACGTTCCCGGGCCTTGTACACACCGC

CCGTCACACCACGAGAGTTTGTAACACCCGAAGTCGGTGAGGTAACCTTTGGAGCC

>MN238728.1 Rossellomorea aquimaris strain MS1-1 16S ribosomal RNA gene, partial sequence

GTCGAGCGGATTGATGGGAGCTTGCTCCCTGATATCAGCGGCGGACGGGTGAGTAACACGTGGGTAACCT

GCCTGTAAGACTGGGATAACTCCGGGAAACCGGGGCTAATACCGGATAACTCATTTCCTCGCATGAGGAA

ATGTTGAAAGATGGCTTCTTGCTATCACTTACAGATGGACCCGCGGCGCATTAGCTAGTTGGTGAGGTAA

CGGCTCACCAAGGCAACGATGCGTAGCCGACCTGAGAGGGTGATCGGCCACACTGGGACTGAGACACGGC

CCAGACTCCTACGGGAGGCAGCAGTAGGGAATCTTCCGCAATGGACGAAAGTCTGACGGAGCAACGCCGC

GTGAGTGATGAAGGTTTTCGGATCGTAAAACTCTGTTGTTAGGGAAGAACAAGTACCGTTCGAATAGGGC

GGTACCTTGACGGTACCTAACCAGAAAGCCACGGCTAACTACGTGCCAGCAGCCGCGGTAATACGTAGGT

GGCAAGCGTTGTCCGGAATTATTGGGCGTAAAGCGCGCGCAGGTGGTTTCTTAAGTCTGATGTGAAAGCC

CACGGCTCAACCGTGGAGGGTCATTGGAAACTGGGGAACTTGAGTGCAGAAGAGGAAAGTGGAATTCCAA

GTGTAGCGGTGAAATGCGTAGATATTTGGAGGAACACCAGTGGCGAAGGCGACTTTCTGGTCTGTAACTG

ACACTGAGGCGCGAAAGCGTGGGGAGCAAACAGGATTAGATACCCTGGTAGTCCACGCCGTAAACGATGA

GTGCTAAGTGTTAGGGGGTTTCCGCCCCTTAGTGCTGCAGCTAACGCATTAAGCACTCCGCCTGGGGAGT

ACGGTCGCAAGACTGAAACTCAAAGGAATTGACGGGGGCCCGCACAAGCGGTGGAGCATGTGGTTTAATT

CGAAGCAACGCGAAGAACCTTACCAGGTCTTGACATCCTCTGACAACCCTAGAGATAGGGCTTTCCCCTT

CGGGGGACAGAGTGACAGGTGGTGCATGGTTGTCGTCAGCTCGTGTCGTGAGATGTTGGGTTAAGTCCCG

CAACGAGCGCAACCCTTGATCTTAGTTGCCAGCATTCAGTTGGGCACTCTAAGATGACTGCCGGTGACAA

ACCGGAGGAAGGTGGGGATGACGTCAAATCATCATGCCCCTTATGACCTGGGCTACACACGTGCTACAAT

GGACGGTACAAAGGGCAGCAAGACCGCGAGGTTTAGCCAATCCCATAAAACCGTTCTCAGTTCGGATTGT

AGGCTGCAACTCGCCTACATGAAGCTGGAATCGCTAGTAATCGCGGATCAGCATGCCGCGGTGAATACGT

TCCCGGGCCTTGTACACACCGCCCGTCACACCACGAGAGTTTGTAACACCCGAAGTCGGTGAGGTAACCT

TTTGGAGCCAGCCGCTAAGGTGGGACAAGATGATTGG
