# Supplementary material for: Molecular Identification of Culturable Bacteria and Vibrio Virulence‐Related Genes Associated With Sea Turtle Eggshells and Nest Sediments in Pangandaran, Indonesia
Source: Int J Microbiol. 2026 Oct 5;2026:7422673. doi: 10.1155/ijm/7422673 (PMC13639395; doi:10.1155/ijm/7422673)
Supplement: Supplementary file 2 [file IJM-2026-7422673-s002.zip › Hasil NCBI/Ws- consensus .docx]

>JQ833503.1 Pseudomonas corrugata strain p17_D02 16S ribosomal RNA gene, partial sequence

CGTAAGCGCGCGTAGGTGGTTCGTTAAGTTGGATGTGAAAGCCCCGGGCTTAACCTGGGAACTGCATTCA

AAACTGACGAGCTAGAGTATGGTAGAGGGTGGTGGAATTTCCTGTGTAGCGGTGAAATGCGTACATATAG

GAAGGAACACCAGTGGCGAAGGCGACCACCTGGACTGATACTGACACTGAGGTGCGAAAGCGTGGGGAGC

AAACAGGATTAGATACCCTGGTATTCCACGCCGTAAACGATGTCAACTAGCCGTTGGGAGCCTTGAGCTC

TTAGTGGCGCAGCTAACGCATTAAGTTGACCGCCTGGGGAGTACGGCCGCAAGGTTAAAACTCAAATGAA

TTGACTGGGGCCCGCACACGCGGTGGAGCATGTGGTTTAATTCGAAGCAACACAAAGAACCTTACCAAGC

CTTGACATCCAATGAACTTTCCTTAGATGGATTGGTGCCTTCGGGAGCATTGAGACAGGTGCTGCATGGC

TGTCGTCTGCTCGTGT

B>KC715834.1 Rhodococcus sp. BRILB51 16S ribosomal RNA gene, partial sequence

CGCCGTTTGTCTGCGTCGTTCGTCACCACTTGGCTGCCCTATTCCAAGCTTGTCGATGATGCGGGCACTA

CTTGAGTACTGCAGGGGAGACTGTACTTCCTGGTGTAGCGGTGAAATGCGCAGATATCGAGAGGAACACC

GGTGGCGGACGCCGGTCTCTGGGCCGTAACTGACGCTGAGGAGCGAAAGCGTGAGTAGCGAACAGGATTC

GATACCCTGGCAGTCCACGCCGTAAACGGCGGGCGCTAGGTGTGGGTTTCCTTCCACTGGATCCGCGCCG

TACCTAACGCATTAACCGCCCCGCCTGGGGAGTACGGCCACTAGGCCAAAACTCAAATGAATTGACGCCG

CCCCGCACAAGCGGCGGAGCATGTGGATTAATTCCACGCAACGCGAAGAACCTTACCTGGGTTTGACATA

TACCGGAAAGACGTAGAGATACGGCCCCCCTTGTGGTCAGTATACAGGTGGTGCATGGCTGTCATCTGCT

CGTGTCGTGAGATGTTGGGTTAAGTCCCGCAACGACCGCCACCTTTGTTCTGTGCTGCCAGCGCGTAATG

GCGGCTACTCGGAGGAGACTGCCGGGGTCAACTCGGAGGAAGGTGGGGACGACATCAAGTCATCATCGCC

CCCTTACGTCCACGGCTTCACACATGCTACAATGGCCGGTACAGAGGGCTGCAATACCGTGAGGTGCAGC

GAATCCCTTAAAGCCGGTCTCAGTTCGGATCGCGGCCTGCCACTCGACCCCGTGAAGTTCTGATCCCCTT

ATAATCGCAGATCAGCAACGCTGCCGTGAATACGTTTCCCCGCCCCTCCCACCCCGCCCGTCATGTCATG

AATGTGCGTAACCCCCAAAACCGGTGGCCTAACCCTTGTTGATGGAGTCCTCCAAGGGG

>KT150075.1 Lysinibacillus sp. ErII_B2.9 16S ribosomal RNA gene, partial sequence

GAGCTTGCTCCTTTGACGTTAGCGGCGGACGGGTGAGTAACACGTGGGCAACCTACCCTATAGTTTGGGA

TAACTCCGGGAAACCGGGGCTAATACCGAATAATCTTTTGTCCCTCATGGGACAATACTGAAAGACGGTT

TCGGCTGTCGCTATAAGATGGGCCCGCCGCGCATTATCTAGTTGGTGAGGTAACGGCTCACCAAGGCCAC

GATGCGTAGCCCACCTGAGAGGGTGATCGGCCACACTGGGACTGAAACACGGCCCACACTCCTACGGGAG

GCAGCAGTAGGGAATCTTCCACAATGGGCGAAAGCCTGGTGGAACCACCCCGCGTGAGTGAAGAAGGATT

TCAGTTCGTAAAACTCTGTTGTAAGGGAAGAACAAGTACAATAGTAACTGGGTGTACCTTGACGGTACCT

TATTAGAAAGCCACGGCTAACTACGTGCCAACAGCCGCGGTAATACGTATGTGGCAAGCGTTGTCCGGAA

TTATTGGGCGTAAAGCGCGCGCAGGTGGTTTCTTAAGTCTGATGTGAAAGCCCACGGCTCAACCGGGGAG

GGTCATTGGAAACTGGGAGACTTGAGTGCAAAAGAAGATAGTGGAATTCCAAGTGTAGCGGTGAAATGCG

TAGAGATTTAGAAGAACACCAGTGGCGAAGGCGACTATCTGGTCTGTAACTGACACTGAAGCGCAAAAGC

GTGGGGAGCAAACAAGATTAGATACCCTGGTAGTCCACGCCGTAAACGATGAGTGCTAAGTGTTAGGGGG

TTTCCGCCCCTTAGTGCTGCATCTAACGCATTACACACTCCGCCTGGAGAGTACGGTCGCAAGACTGAAA

CTCAAAGGAATTGACGGG
