# Supplementary material for: Molecular Identification of Culturable Bacteria and Vibrio Virulence‐Related Genes Associated With Sea Turtle Eggshells and Nest Sediments in Pangandaran, Indonesia
Source: Int J Microbiol. 2026 Oct 5;2026:7422673. doi: 10.1155/ijm/7422673 (PMC13639395; doi:10.1155/ijm/7422673)
Supplement: Supplementary file 2 [file IJM-2026-7422673-s002.zip › Hasil NCBI/MS Cosenssus .docx]

```
 >,.1 Vibrio alginolyticus strain AA62 16S ribosomal RNA gene, partial sequence
ATGCAGTCGAGCGGAACGAGTTATCTGAACCTTCGGGGAACGATAACGGCGTCGAGCGGCGGACGGGTGA
GTAATGCCTAGGAAATTGCCCTGATGTGGGGGATAACCATTGGAAACGATGGCTAATACCGCATGATGCC
TACGGGCCAAAGAGGGGGACCTTCGGGCCTCTCGCGTCAGGATATGCCTAGGTGGGATTAGCTAGTTGGT
GAGGTAAGGGCTCACCAAGGCGACGATCCCTAGCTGGTCTGAGAGGATGATCAGCCACACTGGAACTGAG
ACACGGTCCAGACTCCTACGGGAGGCAGCAGTGGGGAATATTGCACAATGGGCGCAAGCCTGATGCAGCC
ATGCCGCGTGTGTGAAGAAGGCCTTCGGGTTGTAAAGCACTTTCAGTCGTGAGGAAGGTAGTGTAGTTAA
TAGCTGCATTATTTGACGTTAGCGACAGAAGAAGCACCGGCTAACTCCGTGCCAGCAGCCGCGGTAATAC
GGAGGGTGCGAGCGTTAATCGGAATTACTGGGCGTAAAGCGCATGCAGGTGGTTTGTTAAGTCAGATGTG
AAAGCCCGGGGCTCAACCTCGGAATAGCATTTGAAACTGGCAGACTAGAGTACTGTAGAGGGGGGTAGAA
TTTCAGGTGTAGCGGTGAAATGCGTAGAGATCTGAAGGAATACCGGTGGCGAAGGCGGCCCCCTGGACAG
ATACTGACACTCAGATGCGAAAGCGTGGGGAGCAAACAGGATTAGATACCCTGGTAGTCCACGCCGTAAA
CGATGTCTACTTGGAGGTTGTGGCCTTGAGCCGTGGCTTTCGGAGCTAACGCGTTAAGTAGACCGCCTGG
GGAGTACGGTCGCAAGATTAAAACTCAAATGAATTGACGGGGGCCCGCACAAGCGGTGGAGCATGTGGTT
TAATTCGATGCAACGCGAAGAACCTTACCTACTCTTGACATCCAGAGAACTTTCCAGAAATGGATTGGTG
CCTTCGGGAACTCTGAAACAGGTGCTGCATGGCTGTCGTCAGCTCGTGTTGTGAAATGTTGGGTTAAGTC
CCGCAACGAGCGCAACCCTTATCCTTGTTTGCCAGCGAGTAATGTCGGGAACTCCAGGGAGACTGCCGGT
GATAAACCGGAGGAAGGTGGGGACGACGTCAAGTCATCATGGCCCTTACGAGTAGGGCTACACACGTGCT
ACAATGGCGCATACAGAGGGCGGCCAACTTGCGAAAGTGAGCGAATCCCAAAAAGTGCGTCGTAGTCCGG
ATTGGAGTCTGCAACTCGACTCCATGAAGTCGGAATCGCTAGTAATCGTGGATCAGAATGCCACGGTGAA
TACGTTCCCGGGCCTTGTACACACCGCCCGTCACACCATGGGAGTGGGCTGCAAAAGAAGTAGGTAGTTT
AACCTTCGGGGGGACGCTTA

>CP082319.1 Vibrio alginolyticus strain XWV9 chromosome 1, complete sequence
GTGTCATCTTCGCTTTGGCTGCAATGTTTGCAGCAGCTTCAAGAAGAGCTACCAGCTACAGAATTCAGCA
TGTGGGTTCGTCCGCTACAAGCGGAGCTCAATGACAATACTCTCACTCTATTCGCGCCAAACCGCTTTGT
GTTGGACTGGGTGCGTGATAAGTATCTGAACAATATTACTCGCTTACTGCAGGAATACTGCGGTGGCGAT
ATTCCTAACTTACGTTTTGAAGTGGGGAGCCGACCTGTCTCTGCACCAAAACCAGCGCCGACACGTACGC
CTGCAGATGTGGCTGCCGAGTCATCTGCACCTGCGCAATTACAGGCGCGTAAGCCAGTACACAAAACCTG
GGATGACGACGCTCAAGCGATTGCGGCTATCAATCACCGCTCAAACATGAACCCAAAACACAAGTTCGAT
AACTTTGTTGAAGGTAAGTCGAACCAACTGGGTTTGGCTGCGGCGCGTCAGGTATCGGATAATCCTGGTG
CAGCATACAACCCGCTGTTCCTATACGGCGGCACGGGCTTGGGTAAAACGCACTTGTTGCATGCGGTGGG
CAACGCGATTGTGGATAACAACCCGAACGCAAAAGTGGTCTACATGCACTCAGAACGTTTTGTGCAAGAC
ATGGTAAAAGCGCTGCAAAACAATGCGATTGAAGAATTCAAACGCTACTACCGCAGCGTGGATGCGCTCC
TTATCGATGACATCCAATTCTTTGCTAATAAAGAACGTTCACAAGAAGAATTCTTCCATACCTTCAACGC
ACTATTGGAAGGTAATCAGCAGATCATTCTAACGTCTGACCGTTATCCTAAAGAAATTAATGGCGTTGAA
GATCGTCTTAAATCTCGCTTTGGTTGGGGTCTTACTGTTGCGATCGAGCCGCCTGAGCTTGAAACACGTG
TGGCGATCTTGATGAAAAAAGCTGAAGATCACCAAATCCACCTTGCTGATGAAGTGGCATTCTTCATTGC
CAAGCGTCTGCGTTCGAACGTTCGTGAGCTAGAAGGTGCACTGAACCGTGTTATCGCGAACGCGAACTTT
ACTGGCCGTCCAATTACGATTGATTTTGTTCGTGAAGCGCTGCGCGATCTGCTTGCTCTACAAGAGAAGC
TTGTGACAATCGATAACATTCAAAAGACCGTGGCGGAATACTACAAGATTAAAGTAGCGGACTTGCTGTC
TAAGCGTCGTTCTCGTTCTGTAGCGCGTCCTCGTCAGCTGGCTATGGCGTTAGCAAAAGAGCTAACCAAC
CACAGTTTGCCTGAAATTGGCGATGCGTTTGGGGGCCGCGATCATACGACCGTACTTCATGCTTGTCGCA
AGATTGAGCAGCTGCGTGAAGAGAGCCACGACATTAAAGAAGATTACTCTAACTTGATTCGCACCCTGTC
TTCGTAATCGGTTAGAATAACGGTATTCTAAGCAAATCATTTATTGAGCTGACAGCGTAAAGAGCCAAGC
ATGAAATTTACCATTGAACGTAGTCATTTAATCAAACCGCTACAGCAGGTTTCGGGCGCTTTAGGTGGCC
GACCAACCCTGCCAATCCTGGGTAACCTACTTCTTAAAGTAGAAGAGAACGTATTGTCGATGACGGCAAC
CGATCTTGAAGTAGAGTTAGTAAGCAAAGTGACGCTAGAAGGTGATTTTGAAGCGGGCAGCATTACCGTT
CCTTCGCGTAAGTTTCTCGATATCTGCCGTGGTTTACCGGACGATGCGATCATCACATTTGTATTAGAAG
GTGATCGCGTACAAGTTCGCTCTGGTCGCAGCCGTTTCTCGTTGGCAACGCTTCCTGCGAACGATTTCCC
TAATATTGAAGACTGGCAAAGCGAAGTGGAAGTATCACTGAGCCAAGCCGATCTACGTACGCTGATCGAC
AAAACTCAGTTCTCGATGGCGAATCAAGACGTTCGTTACTACCTCAACGGCATGTTGTTTGAAATTGACG
GCACCACGTTACGCAGCGTGGCGACGGACGGCCACCGTATGGCGGTATCGCAAACTCAGCTAGGTGCAGA
CTTTGCGCAGAAGCAAATCATCGTGCCACGCAAAGGTGTGCAAGAATTAGTAAAGTTGATGGATGCGCCA
GAGCAGCCTGTAGTACTTCAGATCGGTAGCTCCAATGTTCGTGCTGAGGTGAATAACTTTACTTTTACCT
CTAAGTTGGTTGATGGTCGTTTCCCTGACTACCGCCGTGTATTGCCTCAGAGCACCAATAAGACACTAAT
AGCAAGTTGTGACGAGTTGCGCCAAGCCTTCTCTCGTGCTGCGATTCTTTCCAACGAAAAATTCCGTGGC
GTGCGTGTGAATCTGGCTGATACTGAAATGCGCATAACCGCGAATAACCCAGAGCAAGAAGAAGCGGAAG
AGATGCTTGATGTAACCTTTGAAGGTGACGCCATCGAAATCGGCTTTAACGTAAGCTATGTGCTTGATGT


>PP389753.1 Vibrio alginolyticus strain A2 16S ribosomal RNA gene, partial sequence
GCCTACCATGCAGTCGAGCGGAACGAGTTATCTGAACCTTCGGGGAACGATAACGGCGTCGAGCGGCGGA
CGGGTGAGTAATGCCTAGGAAATTGCCCTGATGTGGGGGATAACCATTGGAAACGATGGCTAATACCGCA
TGATGCCTACGGGCCAAAGAGGGGGACCTTCGGGCCTCTCGCGTCAGGATATGCCTAGGTGGGATTAGCT
AGTTGGTGAGGTAAGGGCTCACCAAGGCGACGATCCCTAGCTGGTCTGAGAGGATGATCAGCCACACTGG
AACTGAGACACGGTCCAGACTCCTACGGGAGGCAGCAGTGGGGAATATTGCACAATGGGCGCAAGCCTGA
TGCAGCCATGCCGCGTGTGTGAAGAAGGCCTTCGGGTTGTAAAGCACTTTCAGTCGTGAGGAAGGTGGTG
TAGTTAATAGCTGCATTATTTGACGTTAGCGACAGAAGAAGCACCGGCTAACTCCGTGCCAGCAGCCGCG
GTAATACGGAGGGTGCGAGCGTTAATCGGAATTACTGGGCGTAAAGCGCATGCAGGTGGTTTGTTAAGTC
AGATGTGAAAGCCCGGGGCTCAACCTCGGAATTGCATTTGAAACTGGCAGACTAGAGTACTGTAGAGGGG
GGTAGAATTTCAGGTGTAGCGGTGAAATGCGTAGAGATCTGAAGGAATACCGGTGGCGAAGGCGGCCCCC
TGGACAGATACTGACACTCAGATGCGAAAGCGTGGGGAGCAAACAGGATTAGATACCCTGGTAGTCCACG
CCGTAAACGATGTCTACTTGGAGGTTGTGGCCTTGAGCCGTGGCTTTCGGAGCTAACGCGTTAAGTAGAC
CGCCTGGGGAGTACGGTCGCAAGATTAAAACTCAAATGAATTGACGGGGGCCCGCACAAGCGGTGGAGCA
TGTGGTTTAATTCGATGCAACGCGAAGAACCTTACCTACTCTTGACATCCAGAGAACTTTCCAGAGATGG
ATTGGTGCCTTCGGGAACTCTGAGACAGGTGCTGCATGGCTGTCGTCAGCTCGTGTTGTGAAATGTTGGG
TTAAGTCCCGCAACGAGCGCAACCCTTATCCTTGTTTGCCACCGAGTAATGTCGGGAACTCCAGGGAAAC
TGCCGGTGAATAACCGGAGGAAGGTGGGGACAACTTCAAGTCATCATGGCCCTTACAAATAGGGCTACAC
ACGTGCTACAATGGCCCATACAGAGGGCGGCCAACTTGCAAATGTGAACCAATCCCAAAAGGCGCGCTCT
ATTCCGGATTGGAGTTCCCACCCCCTCTCCATGAAAACGGAACCCTTAGTAATTTGGGAATAGAATGCCC
CCGGGGAAACGTTCCCCGGCTTTTAACCCCCCCCCCCCCACACCATGGGAGTGGGCTGCAAAAGAAGTAG
GTAGTTTAACCTTCGGGGGGACGCTACCATTGA

>MT490871.1 Vibrio alginolyticus strain ROA011 16S ribosomal RNA gene, partial sequence
TCATGGCTCAGATTGAACGCTGGCGGCAGGCCTAACACATGCAAGTCGAGCGGAAACGAGTTATCTGAAC
CTTCGGGGAACGATAACGGCGTCGAGCGGCGGACGGGTGAGTAATGCCTAGGAAATTGCCCTGATGTGGG
GGATAACCATTGGAAACGATGGCTAATACCGCATGATGCCTACGGGCCAAAGAGGGGGACCTTCGGGCCT
CTCGCGTCAGGATATGCCTAGGTGGGATTAGCTAGTTGGTGAGGTAAGGGCTCACCAAGGCGACGATCCC
TAGCTGGTCTGAGAGGATGATCAGCCACACTGGAACTGAGACACGGTCCAGACTCCTACGGGAGGCAGCA
GTGGGGAATATTGCACAATGGGCGCAAGCCTGATGCAGCCATGCCGCGTGTGTGAAGAAGGCCTTCGGGT
TGTAAAGCACTTTCAGTCGTGAGGAAGGTGGTGTAGTTAATAGCTGCATTATTTGACGTTAGCGACAGAA
GAAGCACCGGCTAACTCCGTGCCAGCAGCCGCGGTAATACGGAGGGTGCGAGCGTTAATCGGAATTACTG
GGCGTAAAGCGCATGCAGGTGGTTTGTTAAGTCAGATGTGAAAGCCCGGGGCTCAACCTCGGAATAGCAT
TTGAAACTGGCAGACTAGAGTACTGTAGAGGGGGGTAGAATTTCAGGTGTAGCGGTGAAATGCGTAGAGA
TCTGAAGGAATACCGGTGGCGAAGGCGGCCCCCTGGACAGATACTGACACTCAGATGCGAAAGCGTGGGG
AGCAAACAGGATTAGATACCCTGGTAGTCCACGCCGTAAACGATGTCTACTTGGAGGTTGTGGCCTTGAG
CCGTGGCTTTCGGAGCTAACGCGTTAAGTAGACCGCCTGGGGAGTACGGTCGCAAGATTAAAACTCAAAT
GAATTGACGGGGGCCCGCACAAGCGGTGGAGCATGTGGTTTAATTCGATGCAACGCGAAGAACCTTACCT
ACTCTTGACATCCAGAGAACTTTCCAGAGATGGATTGGTGCCTTCGGGAACTCTGAGACAGGTGCTGCAT
GGCTGTCGTCAGCTCGTGTTGTGAAATGTTGGGTTAAGTCCCGCAACGAGCGCAACCCTTATCCTTGTTT
GCCAGCGAGTAATGTCGGGAACTCCAGGGAGACTGCCGGTGATAAACCGGAGGAAGGTGGGGACGACGTC
AAGTCATCATGGCCCTTACGAGTAGGGCTACACACGTGCTACAATGGCGCATACAGAGGGCGGCCAACTT
GCGAAAGTGAGCGAATCCCAAAAGTGCGTCGTAGTCCGGATTGGAGTCTGCAACTCGACTCCATGAAGTC
GGAATCGCTAGTAATCGTGGATCAGAATGCCACGGTGAATACGTTCCCGGGCCTTGTACACACCGCCCGT
CACACCATGGGAGTGGGCTGCAAAAGAAGTAGT

>ON003465.1 Vibrio alginolyticus strain 4.1 16S ribosomal RNA gene, partial sequence
TGCAAGTCGAGCGGAAACGAGTTATCTGAACCTTCGGGGGACGATAACGGCGTCGAGCGGCGGACGGGTG
AGTAATGCCTAGGAAATTGCCCTGATGTGGGGGATAACCATTGGAAACGATGGCTAATACCGCATGATGC
CTACGGGCCAAAGAGGGGGACCTTCGGGCCTCTCGCGTCAGGATATGCCTAGGTGGGATTAGCTAGTTGG
TGAGGTAAGGGCTCACCAAGGCGACGATCCCTAGCTGGTCTGAGAGGATGATCAGCCACACTGGAACTGA
GACACGGTCCAGACTCCTACGGGAGGCAGCAGTGGGGAATATTGCACAATGGGCGCAAGCCTGATGCAGC
CATGCCGCGTGTGTGAAGAAGGCCTTCGGGTTGTAAAGCACTTTCAGTCGTGAGGAAGGTGGTGTAGTTA
ATAGCTGCATTATTTGACGTTAGCGACAGAAGAAGCACCGGCTAACTCCGTGCCAGCAGCCGCGGTAATA
CGGAGGGTGCGAGCGTTAATCGGAATTACTGGGCGTAAAGCGCATGCAGGTGGTTTGTTAAGTCAGATGT
GAAAGCCCGGGGCTCAACCTCGGAATAGCATTTGAAACTGGCAGACTAGAGTACTGTAGAGGGGGGTAGA
ATTTCAGGTGTAGCGGTGAAATGCGTAGAGATCTGAAGGAATACCGGTGGCGAAGGCGGCCCCCTGGACA
GATACTGACACTCAGATGCGAAAGCGTGGGGAGCAAACAGGATTAGATACCCTGGTAGTCCACGCCGTAA
ACGATGTCTACTTGGAGGTTGTGGCCTTGAGCCGTGGCTTTCGGAGCTAACGCGTTAAGTAGACCGCCTG
GGGAGTACGGTCGCAAGATTAAAACTCAAATGAATTGACGGGGGCCCGCACAAGCGGTGGAGCATGTGGT
TTAATTCGATGCAACGCGAAGAACCTTACCTACTCTTGACATCCAGAGAACTTTCCAGAGATGGATTGGT
GCCTTCGGGAACTCTGAGACAGGTGCTGCATGGCTGTCGTCAGCTCGTGTTGTGAAATGTTGGGTTAAGT
CCCGCAACGAGCGCAACCCTTATCCTTGTTTGCCAGCGAGTAATGTCGGGAACTCCAGGGAAACTGCCGG
TGATAAACCGGAGGAAGGTGGGGACGACGTCAAGTCATCATGGCCCTTACAAGTAGGCTAGCTACACACG
TGCTACAATGGCGCATACAGAGGGCGGCCAACTTGCGAAAGTGAGCGAATCCCAAAAAGTGCGTCGTAGT
CCGGATTGGAGTCTGCAACTCGACTCCATGAAGTCGGAATCGCTAGTAATCGTGGATCAGAATGCCACGG
TGAATACGTTCCCGGGCCTTGTACACACCGCCCGTCACACCATGGGAGTGGGCTGCAAAAGAAGTAGGTA
GTTTAACCTTCGGGGGACGCTTACCCACTTT
```
