# Supplementary material for: Molecular Identification of Culturable Bacteria and Vibrio Virulence‐Related Genes Associated With Sea Turtle Eggshells and Nest Sediments in Pangandaran, Indonesia
Source: Int J Microbiol. 2026 Oct 5;2026:7422673. doi: 10.1155/ijm/7422673 (PMC13639395; doi:10.1155/ijm/7422673)
Supplement: Supplementary file 2 [file IJM-2026-7422673-s002.zip › Hasil NCBI/FS consensus.docx]

>PQ496001.1 Providencia rettgeri strain CR 16S ribosomal RNA gene, partial sequence

TGCAGTCGAGCGGTAACAGGGGAAGCTTGCTTCTCGCTGACGAGCGGCGGACGGGTGAGTAATGTATGGG

GATCTGCCCGATAGAGGGGGATAACTACTGGAAACGGTAGCTAATACCGCATAATCTCTCAGGAGCAAAG

CAGGGGAACTTCGGTCCTTGCGCTATCGGATGAACCCATATGGGATTAGCTAGTAGGTGAGGTAATGGCT

CACCTAGGCGACAATCCCTAGCTGGTCTGAGAGGATGATCAGCCACACTGGGACTGAGACACGGCCCACA

CTCCTACAGGAGGCAGCAGTGGGGAATATTGCACAATGGGCGCAAGCCTGATGCACCCATGCCGCGTGTA

TGAAAAAGGCCCTAGGGTTGTAAAGTACTTTCACTCGGGAGGAAGGCGTTGATGCTAATATCATCAACGA

TTGACGTTACCGACAGAAAAAACACCGGCTAACTCCGTGCCAGCAGCCGCGGTAATACGGAGGGTGCCAA

GCGTTAATCGGAATTACTGGGCGTAAAGCGCACGCAGGCGGTTGATTAAGTTTAGATGTGAAATCCCCGG

GCTTAACCTGGGAATGGCATCTAAGACTGGTCAGCTAGAGTCTTGTAGAGGGGGGTAGAATTCCATGTGT

AGCGGTGAAATGCGTAGAGATGTGGAGGAATACCGGTGGCGAAGGCGGCCCCCTGGACAAAGACTGACGC

TCAGGTGCGAAAGCGTGGGGAGCAAACAGGATTAGATACCCTGGTAGTCCACGCTGTAAACGATGTCGAT

TTGAAGGTTGTTCCCTAGAGGAGTGGCTTTCGGAGCTAACGCGTTATATCGACCGCCTGGGGAGTACGGC

CGCAAGGTTAAAACTCAAATGAATTGACGGGGGCCCGCACAAGCGGTGGAGCATGTGGTTTAATTCGATG

CAACGCGAAGAACCTTACCTACTCTTGACATCCAGAGAATTTAGCAGAGATGCTTTGGTCCCTTCGGGAA

CTTTGAGACAGGTGCTGCATGGCTGTCGTCAGCTCGTGTTGTGAAATGTTGGGTTAAGTCCCGCAACGAG

CGCAACCCTTATCCTTTGTTGCCAGCGATTCGGTCGGGAACTCAAAGGAGACTGCCGGTGATAAACCGGA

GGAAGGTGGGGATGACGTCAAGTCATCATGGCCCTTACGAGTAGGGCTACACACGTGCTACAATGGCGTA

TACAAAGAGAAGCGACCTCGCGAGAGCAAGCGGAACTCATAAAGTACGTCGTAGTCCGGATTGGAGTCTG

CAACTCGACTCCATGAAGTCGGAATCGCTAGTAATCGTAGATCAGAATGCTACGGTGAATACGTTCCCGG

GCCTTGTACACACCGCCCGTCACACCATGGGAGTGGGTTGCAAAAGAAGTAGGTAGCTTAACCTTCGGGA

>KX702377.1 Providencia rettgeri strain B4A1 16S ribosomal RNA gene, partial sequence

CTACACATGCAGTCGAGCGGTAACAGGGGAAGCTTGCTTCTCGCTGACGAGCGGCGGACGGGTGAGTAAT

GTATGGGGATCTGCCCGATAGAGGGGGATAACTACTGGAAACGGTAGCTAATACCGCATAATCTCTCAGG

AGCAAAGCAGGGGAACTTCGGTCCTTGCGCTATCGGATGAACCCATATGGGATTAGCTAGTAGGTGAGGT

AATGGCTCACCTAGGCGACAATCCCTATCTGGTCTGAGAGGATGATCACCCACACTGGGACTGAGACACG

GCCCACACTCCTACGGGAGGCAGCAGTGGGGAATATTGCACAATGGGCGCAAGCCTGATGCACCCATGCC

GCGTGTATGAAAAAAGCCCTATGGTTGTAAAGTACTTTCAGTCGGGAGGAAGGCGTTGATGCTAATATCA

TCAACGATTGACGTTACCGACAGAAAAAACACCGGCTAACTCCGTGCCAGCAGCCGCGGTAATACGGAGG

GTGCAAGCGTTAATCGGAATTACTGGGCGTAAAGCGCACGCAGGCGGTTGATTAAGTTAGATGTGAAATC

CCCGGGCTTAACCTGGGAATGGCATCTAAGACTGGTCAGCTAGAGTCTTGTAGAGGGGGGTAGAATTCCA

TGTGTAGCGGTGAAATGCGTATAGATGTGGAGGAATACCGGTGGCGAAGGCGGCCCCCCTGGACAAAGAC

TGACGCTCACGGTGCGAAAGCGTGTGGGAGCAAACAGGATTATATACCCTGGTAGTCCACGCTGTAAACG

ATGTCTATTTGAAGGTTGTTCCCTAGAGGAGTGGCTTTCGGAGCTAACGCGTTAAATCGACCGCCCTGGG

GAGTACGGCCGCAAGGTTAAAACTAACATGAATTGACGGGGGGCCCGCACAAGCGGTGGAGCATGTGTTT

TATTTCGATGCAACGCGAAGAGCCTTACCTACTCTTGACATCCACAGAATTTAGCAGAGATGCTTTAGTG

CCTTCGGGAACTCTGAGACAGGTGCTGCATGGCTGTCGTCAGCTCGTGTTGTGAAATGTTGGGTTAAGTT

CCGCACCGGGCGCAACCCTTATCCTTTGTTGGCAGCGATTCGG

>OR542989.1 Providencia sp. strain L21 16S ribosomal RNA gene, partial sequence

ACTTTCAGTCGGGAGGAAGGCGTTGATACTAATATCATCAACGATTGACGTTACCGACAGAAAAAACACC

GGCTAACTCCGTGCCAGCAGCCGCGGTAATACAGAGGGTGCAAGCGTTAATCTGAATTACTGGGCGTAAA

GCGCACGCACGCGATTGATTAAGTTAGATGTGAAATCCCCGGGCTTAACCTGGGAATGGCATCTAAGACT

GGTCAGCTATAGTCTTGTAGAGAGGGGTAGAATTCCATGTGTAGCGGTGAAATGCGTAGAAGATGTGGAA

GGAATACCGGTGGCGAARGGCGGCCCCCTGGACAAAAGACTGACRCTCAGGTGCGAAAGCGTGGGGAGCA

AAMAGGATTAGATACCCTGGTAGTCCACGCTGTAAACGATGTCGATTTGAAGGTTGTTCCCTTGAGGAGT

GGCTTTCGGAGCTAACGCGTTAAATCGACCGCCTGGGGAGTACGGCCGCAAGGTTAAAACTCAAATGAAT

TGACGGGGGCCCGCACAAGCGGTGGAGCATGTGGTTTAATTCGATGCAACGCGAAGAACCTTACCTACTC

TTGACATCCAGAGAATTTAGCAGAGATGCTTTAGTGCCTTCGGGAACTCTGAGACAGGTGCTGCATGGCT

GTCGTCAGCTCGTGTTGTGAAATGTTGGGTTAAGTCCCGCAACGAGCGCAACCCTTATCCTTTGTTCCAG

TGATTCGGTCGGGAACTCAAAGGAGACTGCCAGTGATAAACTGGAGGAAGGTGGGGATGACGTCAAGTCA

TCATGGGCTTTAGGTTTAGGGCTACACACGTGCTTCAAT

>GQ060660.1 Uncultured bacterium clone nbw03g09c1 16S ribosomal RNA gene, partial sequence

ATTGAACGCTGGCGGCAGGCCTAACACATGCAAGTCGAACGGCAGCGGGGGATAGCTTGCTATCCTGCCG

GCGAGTGGCGGACGGGTGAGTAAGGCATGGGGAGCTGCCCAATCGAGGGGGATAACAACTGGAAACGGTT

GCTAATACCGCATACGCCCTGCGGGGGAAAGGTGGGGACCTTCGGGCCTACCGCGATTGGATGCACCCAT

GTGGGATTAGCTGGTTGGTGAGGTAATGGCTCACCAAGGCGACGATCTCTAGCTGGTCTGAGAGGATGAC

CAGCCACACTGGAACTGAGACACGGTCCAGACTCCTACGGGAGGCAGCAGTGGGGAATATTGCACAATGG

GGGAAACTCTGATGCAGCCATGCCGCGTGTGTGAAGAAGGCCTTCGGGTTGTAAAGCACTTTCAGTCAGG

AGGAAGGGGAGCAGACTAATATTCCGTTTCATTGACGTTACTGACAGAAGAAGCACCGGCTAACTCCGTG

CCAGCAGCCGCGGTAATACGGAGGGTGCAAGCGTTAATCGCAATAACTGGGCGTAAAGCGCACGCAGGCG

GTTGGATAAGCGAGATGTGAAAGCCCCGGGCTCAACCTGAGAACTGCATTTCGAACTGTCCGACTAGAGT

CTTGTAGAGGGGGGTAGAATTCCAGGTGTAGCGGTGAAATGCGTAGAGATCTGGAGGAATACCGGTGGCG

AAGGCGGCCCCCTGGACAAAGACTGACGCTCAGGTGCGAAAGCGTGGGGAGCAAACAGGATTAGATACCC

TGGTAGTCCACGCTGTAAACGATGTCGATTTGGAGTTTGTGCCATTATGAGCGTGGGTTCCGAAGCTAAC

GCGATAAATCGACCGCCTGGGGAGTACGGCCGCAAGGTTAAAACTCAAATGAATTGACGGGGGCCCGCAC

AAGCGGTGGAGCATGTGGTTTAATTCGATGCAACGCGAAGAACCTTACCTGGCCTTGACATGCTGAGAAC

TTTCCAGAGATGGATTGGTGCCTTCGGGAACTCAGACACAGGTGCTGCATGGCTGTCGTCAGCTCGTGTC

GTGAGATGTTGGGTTAAGTCCCGCAACGAGCGCAACCCCTATCCTTTGTTGCCAGCGGGTAATGCCGGGA

ACTCAAGGGAGACTGCCGGTGATAAACCGGAGGAAGGTGGGGATGACGTCAAGTCATCATGGCCCTTACG

GCCAGGGCTACACACGTGCTACAATGGCGTATACAGAGGGAAGCGACCTCGCGAGAGCAAGCGGAACCCA

AAAAGTACGTCGTAGTCCGGATCGGAGTCTGCAACTCGACTCCGTGAAGTCGGAATCGCTAGTAATCGCA

AATCAGAATGTTGCGGTGAATACGTTCCCGGGCCT

>MN626636.1 Oceanimonas smirnovii strain ST3 16S ribosomal RNA gene, partial sequence

GTAGCTTGCTCCTTTTGCTGACGAGCGGCGGACGGGTGAGTAATGCTTGGGGATCTGCCCGGTCGAGGGG

GATAACCGTTGGAAACGACGGCTAATACCGCATACGCCCTACGGGGGAAAGCAGGGGACCTTCGGGCCTT

GCGCGATTGGATGAACCCAAGTGAGATTAGCTTGTTGGTGAGGTAACGGCTCACCAAGGCGACGATCTCT

AGCTGGTCTGAGAGGATGACCAGTCACACTGGGACTGAGACACGGCCCAGACTCCTACGGGAGGCAGCAG

TGGGGAATATTGCACAATGGGGGAAACCCTGATGCAGCCATGCCGCGTGTGTGAAGAAGGCCTTCGGGTT

GTAAAGCACTTTCAGTGGTGAGGAAAGGCGGTTGGCTAATACCCGACCGCTGTGACGTTAACCACAGAAG

AAGCACCGGCTAACTCCGTGCCAGCAGCCGCGGTAATACGGAGGGTGCAAGCGTTAATCGCAATAACTGG

GCGTAAAGCGCACGCAGGCGGTTTGTTAAGCCAGATGTGAAAGCCCCGGGCTCAACCCGGGAACTGCATT

TGGAACTGGCAGACTAGAGTCTTGGAGAGGGGGGTAGAATTTCCGGTGTAGCGGTGAAATGCGTAGAGAT

CGGAAGGAATACCAGTGGCGAAGGCGGCCCCCTGGCCAAAGACTGACGCTCAGGTGCGAGGCGTGGGGAG

CAAACAGGATTAGATACCCTGGTAGTCCACGCCGTAAACGATGTCAACTTGTAGTCTGTTCCATTTGAGC

GCGGGTTTCGGAGCTAACGCGTTAAGTTGACCGCCTGGGGAGTACGGCCGCAAGGTTAAAACTCAAATGA

ATTGACGGGGGCCCGCACAAGCGGTGGAGCATGTGGTTTAATTCGATGCAACGCGAAGAACCTTACCTAC

CCTTGACATACCAAGAAATTTTCAGAGATGCATTTGTGCCTTCGGGGAATTGTATACAGGTGCTGCATGG

CTGTTGTCAGCTTGTGTTGTGAGATGTTGGGTTAAATCCCGCAACGAGCGCCACCCTTGTCCTTTTTTTC

CCACGGGTTATGCCGGGAACTCAAAGGAGACTGCCGGTGATAAACCGGAGGAAGGTGGGGACGACGTCAA

GTCATCATGGCCCTTACGGGTAGGGCTACCCCCGTGCTACAATGGCGCGTACAGAGGGCAGCGAACTCGC

GAGAGTAAGCGAATCCCAAAAAACCCGTCGTAGTCCCGATCGGAGTTTGCAACTTGACTCCGTGAAGTCG

GAATCGCTAGTAATCGTGGATCAGAATGCCACGGTGAATACGTTCCCGGGCCTTGTACCCCCCCCCCGTC

CCCCCATGGGAGTGGGTTGCTCCAGAAGTAGATAGCTT
