# Supplementary material for: Molecular Identification of Culturable Bacteria and Vibrio Virulence‐Related Genes Associated With Sea Turtle Eggshells and Nest Sediments in Pangandaran, Indonesia
Source: Int J Microbiol. 2026 Oct 5;2026:7422673. doi: 10.1155/ijm/7422673 (PMC13639395; doi:10.1155/ijm/7422673)
Supplement: Supplementary file 2 [file IJM-2026-7422673-s002.zip › Hasil NCBI/Xs- Consensus .docx]

>OL477328.1 Aeromonas sp. strain CQ11 16S ribosomal RNA gene, partial sequence

TGCAAGTCGAGCGGCAGCGGGAAAGTAGCTTGCTACTTTTGCCGGCGAGCGGCGGACGGGTGAGTAATGC

CTGGGGATCTGCCCAGTCGAGGGGGATAACAGTTGGAAACGACTGCTAATACCGCATACGCCCTACGGGG

GAAAGGAGGGGACCTTCGGGCCTTTCGCGATTGGATGAACCCAGGTGGGATTAGCTAGTTGGTGAGGTAA

TGGCTCACCAAGGCGACGATCCCTAGCTGGTCTGAGAGGATGATCAGCCACACTGGAACTGAGACACGGT

CCAGACTCCTACGGGAGGCAGCAGTGGGGAATATTGCACAATGGGGGAAACCCTGATGCAGCCATGCCGC

GTGTGTGAAGAAGGCCTTCGGGTTGTAAAGCACTTTCAGCGAGGAGGAAAGGATGCTGGCTAATATCCAG

CCTCTGTGACGTTACTCGCAGAAGAAGCACCGGCTAACTCCGTGCCAGCAGCCGCGGTAATACGGAGGGT

GCAAGCGTTAATCGGAATTACTGGGCGTAAAGCGCACGCAGGCGGTTGGATAAGTTAGATGTGAAAGCCC

CGGGCTCAACCTGGGAATTGCATTTAAAACTGTCCAGCTAGAGTCTTGTAGAGGGGGGTAGAATTCCAGG

TGTAGCGGTGAAATGCGTAGAGATCTGGAGGAATACCGGTGGCGAAGGCGGCCCCCTGGACAAAGACTGA

CGCTCAGGTGCGAAAGCGTGGGGAGCAAACAGGATTAGATACCCTGGTAGTCCACGCCGTAAACGATGTC

GATTTGGAGGCTGTGTCCTTGAGACGTGGCTTCCGGAGCTAACGCGTTAAATCGACCGCCTGGGGAGTAC

GGCCGCAAGGTTAAAACTCAAATGAATTGACGGGGGCCCGCACAAGCGGTGGAGCATGTGGTTTAATTCG

ATGCAACGCGAAGAACCTTACCTGGCCTTGACATGTCTGGAATCCTGTAGAGATACGGGAGTGCCTTCGG

GAATCAGAACACAGGTGCTGCATGGCTGTCGTCAGCTCGTGTCGTGAGATGTTGGGTTAAGTCCCGCAAC

GAGCGCAACCCCTGTCCTTTGTTGCCAGCACGTAATGGTGGGAACTCAAGGGAGACTGCCGGTGATAAAC

CGGAGGAAGGTGGGGATGACGTCAAGTCATCATGGCCCTTACGGCCAGGGCTACACACGTGCTACAATGG

CGCGTACAGAGGGCTGCAAGCTAGCGATAGTGAGCGAATCCCAAAAAGCGCGTCGTAGTCCGGATCGGAG

TCTGCAACTCGACTCCGTGAAGTCGGAATCGCTAGTAATCGCGAATCAGAATGTCGCGGTGAATACGTTC

CCGGGCCTTGTACACACCGCCCGTCACACCATGGGAGTGGGTTGCACCAGAAGTAGATAGCTTAACCTTC

GGGAGGGCGTTACCACGGGTGA

>NR_043885.1 Aeromonas bivalvium strain 868E 16S ribosomal RNA, partial sequence

AGAGTTTGATCATGGCTCAGATTGAACGCTGGCGGCAGGCCTAACACATGCAAGTCGAGCGGCAGCGGGA

AAGTAGCTTGCTACTTTTGCCGGCGAGCGGCGGACGGGTGAGTAATGCCTGGGGATCTGCCCAGTCGAGG

GGGATAACAGTTGGAAACGACTGCTAATACCGCATACGCCCTACGGGGGAAAGGAGGGGACCTTCGGGCC

TTTCGCGATTGGATGAACCCAGGTGGGATTAGCTAGTTGGTGNGGTAATGGCTCACCAAGGCGACGATCC

CTAGCTGGTCTGAGAGGATGATCAGCCACACTGGAACTGAGACACGGTCCAGACTCCTACGGGAGGCAGC

AGTGGGGAATATTGCACAATGGGGGAAACCCTGATGCAGCCATGCCGCGTGTGTGAAGAAGGCCTTCGGG

TTGTAAAGCACTTTCAGCGAGGAGGAAAGGATGCTGGCTAATATCCAGCATCTGTGACGTTACTCGCAGA

AGAAGCACCGGCTAACTCCGTGCCAGCAGCCGCGGTAATACGGAGGGTGCAAGCGTTAATCGGAATTACT

GGGCGTAAAGCGCACGCAGGCGGTTGGATAAGTTAGATGTGAAAGCCCCGGGCTCAACCTGGGAATTGCA

TTTAAAACTGTCCAGCTAGAGTCTTGTAGAGGGGGGTAGAATTCCAGGTGTAGCGGTGAAATGCGTAGAG

ATCTGGAGGAATACCGGTGGCGAAGGCGGCCCCCTGGACAAAGACTGACGCTCAGGTGCGAAAGCGTGGG

GAGCAAACAGGATTAGATACCCTGGTAGTCCACGCCGTAAACGATGTCGATTTGGAGGCTGTGTCCTTGA

GACGTGGCTTCCGGAGCTAACGCGTTAAATCGACCGCCTGGGGAGTACGGCCGCAAGGTTAAAACTCAAA

TGAATTGACGGGGGCCCGCACAAGCGGTGGAGCATGTGGTTTAATTCGATGCAACGCGAAGAACCTTACC

TGGCCTTGACATGTCTGGAATCCTGTAGAGATACGGGAGTGCCTTCGGGAATCAGAACACAGGTGCTGCA

TGGCTGTCGTCAGCTCGTGTCGTGAGATGTTGGGTTAAGTCCCGCAACGAGCGCAACCCCTGTCCTTTGT

TGCCAGCACGTAATGGTGGGAACTCAAGGGAGACTGCCGGTGATAAACCGGAGGAAGGTGGGGATGACGT

CAAGTCATCATGGCCCTTACGGCCAGGGCTACACACGTGCTACAATGGCGCGTACAGAGGGCTGCAAGCT

AGCGATAGTGAGCGAATCCCAAAAAGCGCGTCGTAGTCCGGATCGGAGTCTGCAACTCGACTCCGTGAAG

TCGGAATCGCTAGTAATCGCAAATCAGAATGTTGCGGTGAATACGTTCCCGGGCCTTGTACACACCGCCC

GTCACACCATGGGAGTGGGTTGCACCAGAAGTAGATAGCTTAACCTTCGGGAGGGCGTTTACCACGGTGT

GATTCATGACTGGGGTGAAGTCGTAACAAGGTAACC

>MW893670.1 Aeromonas bivalvium strain CT-WL5-9 16S ribosomal RNA gene, partial sequence

AGCGGCAGCGGGAAAGTAGCTTGCTACTTTTGCCGGCGAGCGGCGGACGGGTGAGTAATGCCTGGGGATC

TGCCCAGTCGAGGGGGATAACAGTTGGAAACGACTGCTAATACCGCATACGCCCTACGGGGGAAAGGAGG

GGACCTTCGGGCCTTTCGCGATTGGATGAACCCAGGTGGGATTAGCTAGTTGGTGAGGTAATGGCTCACC

AAGGCGACGATCCCTAGCTGGTCTGAGAGGATGATCAGCCACACTGGAACTGAGACACGGTCCAGACTCC

TACGGGAGGCAGCAGTGGGGAATATTGCACAATGGGGGAAACCCTGATGCAGCCATGCCGCGTGTGTGAA

GAAGGCCTTCGGGTTGTAAAGCACTTTCAGCGAGGAGGAAAGGATGCTGGCTAATATCCAGCATCTGTGA

CGTTACTCGCAGAAGAAGCACCGGCTAACTCCGTGCCAGCAGCCGCGGTAATACGGAGGGTGCAAGCGTT

AATCGGAATTACTGGGCGTAAAGCGCACGCAGGCGGTTGGATAAGTTAGATGTGAAAGCCCCGGGCTCAA

CCTGGGAATTGCATTTAAAACTGTCCAGCTAGAGTCTTGTAGAGGGGGGTAGAATTCCAGGTGTAGCGGT

GAAATGCGTAGAGATCTGGAGGAATACCGGTGGCGAAGGCGGCCCCCTGGACAAAGACTGACGCTCAGGT

GCGAAAGCGTGGGGAGCAAACAGGATTAGATACCCTGGTAGTCCACGCCGTAAACGATGTCGATTTGGAG

GCTGTGTCCTTGAGACGTGGCTTCCGGAGCTAACGCGTTAAATCGACCGCCTGGGGAGTACGGCCGCAAG

GTTAAAACTCAAATGAATTGACGGGGGCCCGCACAAGCGGTGGAGCATGTGGTTTAATTCGATGCAACGC

GAAGAACCTTACCTGGCCTTGACATGTCTGGAATCCTGCAGAGATNCGGGAGTGCCTTCGGGAATCAGAA

CACAGGTGCTGCATGGCTGTCGTCAGCTCGTGTCGTGAGATGTTGGGTTAAGTCCCGCAACGAGCGCAAC

CCCTGTCCTTTGTTGCCAGCACGTAATGGTGGGAACTCAAGGGAGACTGCCGGTGATAAACCGGAGGAAG

GTGGGGATGACGTCAAGTCATCATGGCCCTTACGGCCAGGGCTACACACGTGCTACAATGGCGCGTACAG

AGGGCTGCAAGCTAGCGATAGTGAGCGAATCCCAAAAAGCGCGTCGTAGTCCGGATCGGAGTCTGCAACT

CGACTCCGTGAAGTCGGAATCGCTAGTAATCGCGAATCAGAATGTCGCGGTGAATACGTTCCCGGGCCTT

GTACACACCGCCCGTCACACCATGGGAGTGGGTTGCACCAGAAGTAGATAGCT

>OR603974.1 Aeromonas bivalvium strain 5C9 16S ribosomal RNA gene, partial sequence

GCGGCAGCGGGAAAGTAGCTTGCTACTTTTGCCGGCGAGCGGCGGACGGGTGAGTAATGCCTGGGGATCT

GCCCAGTCGAGGGGGATAACAGTTGGAAACGACTGCTAATACCGCATACGCCCTACGGGGGAAAGGAGGG

GACCTTCGGGCCTTTCGCGATTGGATGAACCCAGGTGGGATTAGCTAGTTGGTGAGGTAATGGCTCACCA

AGGCGACGATCCCTAGCTGGTCTGAGAGGATGATCAGCCACACTGGAACTGAGACACGGTCCAGACTCCT

ACGGGAGGCAGCAGTGGGGAATATTGCACAATGGGGGAAACCCTGATGCAGCCATGCCGCGTGTGTGAAG

AAGGCCTTCGGGTTGTAAAGCACTTTCAGCGAGGAGGAAAGGATGCTGGCTAATATCCAGCATCTGTGAC

GTTACTCGCAGAAGAAGCACCGGCTAACTCCGTGCCAGCAGCCGCGGTAATACGGAGGGTGCAAGCGTTA

ATCGGAATTACTGGGCGTAAAGCGCACGCAGGCGGTTGGATAAGTTAGATGTGAAAGCCCCGGGCTCAAC

CTGGGAATTGCATTTAAAACTGTCCAGCTAGAGTCTTGTAGAGGGGGGTAGAATTCCAGGTGTAGCGGTG

AAATGCGTAGAGATCTGGAGGAATACCGGTGGCGAAGGCGGCCCCCTGGACAAAGACTGACGCTCAGGTG

CGAAAGCGTGGGGAGCAAACAGGATTAGATACCCTGGTAGTCCACGCCGTAAACGATGTCGATTTGGAGG

CTGTGTCCTTGAGACGTGGCTTCCGGAGCTAACGCGTTAAATCGACCGCCTGGGGAGTACGGCCGCAAGG

TTAAAACTCAAATGAATTGACGGGGGCCCGCACAAGCGGTGGAGCATGTGGTTTAATTCGATGCAACGCG

AAGAACCTTACCTGGCCTTGACATGTCTGGAATCCTGCAGAGATGCGGGAGTGCCTTCGGGAATCAGAAC

ACAGGTGCTGCATGGCTGTCGTCAGCTCGTGTCGTGAGATGTTGGGTTAAGTCCCGCAACGAGCGCAACC

CCTGTCCTTTGTTGCCAGCACGTAATGGTGGGAACTCAAGGGAGACTGCCGGTGATAAACCGGAGGAAGG

TGGGGATGACGTCAAGTCATCATGGCCCTTACGGCCAGGGCTACACACGTGCTACAATGGCGCGTACAGA

GGGCTGCAAGCTAGCGATAGTGAGCGAATCCCAAAAAGCGCGTCGTAGTCCGGATCGGAGTCTGCAACTC

GACTCCGTGAAGTCGGAATCGCTAGTAATCGCAAATCAGAATGTTGCGGTGAATACGTTCCCGGGCCTTG

TACACACCGCCCGTCACACCATGGGAGTGGGTTGCACCAGAAGTAGTATAGC

>LC737961.1 Aeromonas bivalvium SK6610 gene for 16S rRNA, partial sequence

GTCGAGCGGCAGCGGGAAAGTAGCTTGCTACTTTTGCCGGCGAGCGGCGGACGGGTGAGTAATGCCTGGG

GATCTGCCCAGTCGAGGGGGATAACAGTTGGAAACGACTGCTAATACCGCATACGCCCTACGGGGGAAAG

GAGGGGACCTTCGGGCCTTTCGCGATTGGATGAACCCAGGTGGGATTAGCTAGTTGGTGAGGTAATGGCT

CACCAAGGCGACGATCCCTAGCTGGTCTGAGAGGATGATCAGCCACACTGGAACTGAGACACGGTCCAGA

CTCCTACGGGAGGCAGCAGTGGGGAATATTGCACAATGGGGGAAACCCTGATGCAGCCATGCCGCGTGTG

TGAAGAAGGCCTTCGGGTTGTAAAGCACTTTCAGCGAGGAGGAAAGGATGCTGGCTAATATCCAGCATCT

GTGACGTTACTCGCAGAAGAAGCACCGGCTAACTCCGTGCCAGCAGCCGCGGTAATACGGAGGGTGCAAG

CGTTAATCGGAATTACTGGGCGTAAAGCGCACGCAGGCGGTTGGATAAGTTAGATGTGAAAGCCCCGGGC

TCAACCTGGGAATTGCATTTAAAACTGTCCAGCTAGAGTCTTGTAGAGGGGGGTAGAATTCCAGGTGTAG

CGGTGAAATGCGTAGAGATCTGGAGGAATACCGGTGGCGAAGGCGGCCCCCTGGACAAAGACTGACGCTC

AGGTGCGAAAGCGTGGGGAGCAAACAGGATTAGATACCCTGGTAGTCCACGCCGTAAACGATGTCGATTT

GGAGGCTGTGTCCTTGAGACGTGGCTTCCGGAGCTAACGCGTTAAATCGACCGCCTGGGGAGTACGGCCG

CAAGGTTAAAACTCAAATGAATTGACGGGGGCCCGCACAAGCGGTGGAGCATGTGGTTTAATTCGATGCA

ACGCGAAGAACCTTACCTGGCCTTGACATGTCTGGAATCCTGCAGAGATGCGGGAGTGCCTTCGGGAATC

AGAACACAGGTGCTGCATGGCTGTCGTCAGCTCGTGTCGTGAGATGTTGGGTTAAGTCCCGCAACGAGCG

CAACCCCTGTCCTTTGTTGCCAGCACGTAATGGTGGGAACTCAAGGGAGACTGCCGGTGATAAACCGGAG

GAAGGTGGGGATGACGTCAAGTCATCATGGCCCTTACGGCCAGGGCTACACACGTGCTACAATGGCGCGT

ACAGAGGGCTGCAAGCTAGCGATAGTGAGCGAATCCCAAAAAGCGCGTCGTAGTCCGGATCGGAGTCTGC

AACTCGACTCCGTGAAGTCGGAATCGCTAGTAATCGCAAATCAGAATGTTGCGGTGAATACGTTCCCGGG

CCTTGTACACACCGCCCATCACACCATGGGAGTGGGTTGCACCAGAAGTAGATAGCTTAACCTTCGGGAG

GGCGTTA
