# Supplementary material for: Molecular Identification of Culturable Bacteria and Vibrio Virulence‐Related Genes Associated With Sea Turtle Eggshells and Nest Sediments in Pangandaran, Indonesia
Source: Int J Microbiol. 2026 Oct 5;2026:7422673. doi: 10.1155/ijm/7422673 (PMC13639395; doi:10.1155/ijm/7422673)
Supplement: Supplementary file 2 [file IJM-2026-7422673-s002.zip › Hasil NCBI/PS Consensus .docx]

>OQ570258.1 Marinobacter sp. strain S2BR75-25.1 16S ribosomal RNA gene, partial sequence

TGCAAGTCGAGCGGTAACAGGGGGAGCTTGCTCCCCGCTGACGAGCGGCGGACGGGTGAGTAATGCTTAG

GAAACTGCCCAGTAGTGGGGGATAGCCCGGGGAAACCCGGATTAATACCGCGTACGCCCTTCGGGGGAAA

GCAGGGGATCTTCGGACCTTGCGCTATTGGATGTGCCTAAGTCGGATTAGCTAGTTGGTGGGGTAAAGGC

CTACCAAGGCGACGATCCGTAGCTGGTCTGAGAGGATGATCAGCCACATCGGGACTGAGACACGGCCCGA

ACTCCTACGGGAGGCAGCAGTGGGGAATATTGGACAATGGGGGCAACCCTGATCCAGCCATGCCGCGTGT

GTGAAGAAGGCTTTCGGGTTGTAAAGCACTTTCAGTGAGGAGGAAGGCTCTAAGGTTAATACCCTTTGGG

ATTGACGTTACTCACAGAAGAAGCACCGGCTAACTCCGTGCCAGCAGCCGCGGTAATACGGAGGGTGCAA

GCGTTAATCGGAATTACTGGGCGTAAAGCGCGCGTAGGTGGTTTGATAAGCGAGATGTGAAAGCCCCGGG

CTTAACCTGGGAACGGCATTTCGAACTGTCAGGCTAGAGTGTGGTAGAGGGTAGTGGAATTTCCTGTGTA

GCGGTGAAATGCGTAGATATAGGAAGGAACACCAGTGGCGAAGGCGGCTACCTGGACCAACACTGACACT

GAGGTGCGAAAGCGTGGGGAGCAAACAGGATTAGATACCCTGGTAGTCCACGCCGTATACGATGTCTACT

AGCCGTTGGGACTCTTGAAGTCTTAGTGGCGCAGCTAACGCACTAAGTTGACCGCCTGGGGAGTACGGCC

GCAAGGTTAAAACTCAAATGAATTGACGGGGGCCCGCACAAGCGGTGGAGCATGTGGTTTAATTCGACGC

AACGCGAAGAACCTTACCTGGCCTTGACATGCACAGAACTTTCCAGAGATGGATTGGTGCCTTCGGGAAC

TCTGACACAGGTGCTGCATGGCCGTCGTCAGCTCGTGTCATGAGATGTTGGGTTAAGTCCCGTAACGAGC

GCAACCCCTATCCCTAGTTGCTAGCAGTTCGGCTGAAAACTCTAGGGAAACTGCCGGTGACAAACCGGAG

GAAGGGGGGGAT

>OQ570230.1 Marinobacter sp. strain 2APP75-4 16S ribosomal RNA gene, partial sequence

TGCAAGTCGAGCGGTAACAGGTCCTTCGGGATGCTGACGAGCGGCGGACGGGTGAGTAATGCTTAGGAAT

CTGCCTGGTAGTGGGGGATAGCCCGGGGAAACCCGGATTAATACCGCGTACGCCCTTCGGGGGAAAGCAG

GGGATCTTCGGACCTTGCGCTATCAGATGAGCCTAAGCCGGATTAGCTAGTTGGTGGGGTAAAGGCCCAC

CAAGGCGACGATCCGTAGCTGGTCTGAGAGGATGATCAGCCACATCGGGACTGAGACACGGCCCGAACTC

CTACGGGAGGCAGCAGTGGGGAATATTGGACAATGGGGGCAACCCTGATCCAGCCATGCCGCGTGTGTGA

AGAAGGCTTTCGGGTTGTAAAGCACTTTCAGTGAGGAGGAAGGCCTTAAGGTTAATACCCTTGAGGATTG

ACGTTACTCACAGAAGAAGCACCGGCTAACTCCGTGCCAGCAGCCGCGGTAATACGGAGGGTGCAAGCGT

TAATCGGAATTACTGGGCGTAAAGCGCGCGTAGGTGGTTTGATAAGCGAGATGTGAAAGCCCCGGGCTTA

ACCTGGGAACGGCATTTCGAACTGTCAGGCTAGAGTGTGGTAGAGGGGTGTGGAATTTCCTGTGTAGCGG

TGAAATGCGTAGATATAGGAAGGAACACCAGTGGCGAAGGCGGCACCCTGGACCAACACTGACACTGAGG

TGCGAAAGCGTGGGGAGCAAACAGGATTAGATACCCTGGTAGTCCACGCCGTAAACGATGTCAACTAGCC

GTTGGGACTCTTGAAGTCTTAGTGGCGCAGCTAACGCACTAAGTTGACCGCCTGGGGAGTACGGCCGCAA

GGTTAAAACTCAAATGAATTGACGGGGGCCCGCACAAGCGGTGGAGCATGTGGTTTAATTCGACGCAACG

CGAAGAACCTTACCTGGCCTTGACATGCTGAGAACTTTCCAGAGATGGATTGGTGCCTTCGGGAACTCAG

ACACAGGTGCTGCATGGCCGTCGTCAGCTCGTGTCGTGAGATGTTGGGTTAAGTCCCGTAACGAGCGCAA

CCCCTATCCCTAATTGCTAGCAGTTTGGCTGAAAACTCTAGGGAAACTGCCGGTGACAAACCGG

>OQ570255.1 Marinobacter sp. strain S2AR25-21 16S ribosomal RNA gene, partial sequence

ATGCAAGTCGAGCGGTAACAGGGGGTGCTTGCACCCTGCTGACGAGCGGCGGACGGGTGAGTAATGCATA

GGAATCTGCCCAGTAGTTGGGGATAGCCCGGGGAAACCCGGATTAATACCGCGTACGCCCTTCGGGGGAA

AGCAGGGGATCTTCGGACCTTGCGCTATTGGATGAGCCTATGTCGGATTAGCTAGTTGGTGGGGTAACGG

CCTACCAAGGCGACGATCCGTAGCTGGTCTGAGAGGATGATCAGCCACATCGGGACTGAGACACGGCCCG

AACTCCTACGGGAGGCAGCAGTGGGGAATATTGGACAATGGGGGCAACCCTGATCCAGCCATGCCGCGTG

TGTGAAGAAGGCTTTCGGGTTGTAAAGCACTTTCAGTGAGGAGGAAAACTTGGTGGCTAATACCCGTGAG

GCTTGACGTTACTCACAGAAGAAGCACCGGCTAACTCCGTGCCAGCAGCCGCGGTAATACGGAGGGTGCA

AGCGTTAATCGGAATTACTGGGCGTAAAGCGCGCGTAGGTGGTTTGATAAGCGAGATGTGAAAGCCCCGG

GCTTAACCTGGGAACGGCATTTCGAACTGTCAGGCTAGAGTGTGGTAGAGGGTAGTGGAATTTCCTGTGT

AGCGGTGAAATGCGTAGATATAGGAAGGAACACCAGTGGCGAAGGCGGCTACCTGGACCAACACTGACAC

TGAGGTGCGAAAGCGTGGGGAGCAAACAGGATTAGATACCCTGGTAGTCCACGCCGTAAACGATGTCAAC

TAGCCGTTGGGACTCTTGAATCCTTAGTGGCGCAGCTAACGCACTAAGTTGACCGCCTGGGGAGTACGGC

CGCAAGGTTAAAACTCAAATGAATTGACGGGGGCCCGCACAAGCGGTGGAGCATGTGGTTTAATTCGACG

CAACGCGAAGAACCTTACCTGGCCTTGACATGCTGAGAACTTTCCAGAGATGGATTGGTGCCTTCGGGAA

CTCTGNGACAGGTGCTGCATGGCCGTCGTCAGCTCGTGTCGTGAGATGTTGGGTTAAGTCCCGTAACGAG

CGCACCCCTATCCCTAGTTGCTAGCAGTTCGGCTGAAAACTCTAGGGAAACTGCCGGTGACAACCGGAGG

AAGG

>OQ570224.1 Marinobacter sp. strain 2APP15-2 16S ribosomal RNA gene, partial sequence

TGCAAGTCGAGCGGTAACAGGTCCTTCGGGATGCTGACGAGCGGCGGACGGGTGAGTAATGCTTAGGAAT

CTGCCTGGTAGTGGGGGATAGCCCGGGGAAACCCGGATTAATACCGCGTACGCCCTTCGGGGGAAAGCAG

GGGATCTTCGGACCTTGCGCTATCAGATGAGCCTAAGCCGGATTAGCTAGTTGGTGGGGTAAAGGCCCAC

CAAGGCGACGATCCGTAGCTGGTCTGAGAGGATGATCAGCCACATCGGGACTGAGACACGGCCCGAACTC

CTACGGGAGGCAGCAGTGGGGAATATTGGACAATGGGGGCAACCCTGATCCAGCCATGCCGCGTGTGTGA

AGAAGGCTTTCGGGTTGTAAAGCACTTTCAGTGAGGAGGAAGGCCTTAAGGTTAATACCCTTGAGGATTG

ACGTTACTCACAGAAGAAGCACCGGCTAACTCCGTGCCAGCAGCCGCGGTAATACGGAGGGTGCAAGCGT

TAATCGGAATTACTGGGCGTAAAGCGCGCGTAGGTGGTTTGATAAGCGAGATGTGAAAGCCCCGGGCTTA

ACCTGGGAACGGCATTTCGAACTGTCAGGCTAGAGTGTGGTAGAGGGGTGTGGAATTTCCTGTGTAGCGG

TGAAATGCGTAGATATAGGAAGGAACACCAGTGGCGAAGGCGGCACCCTGGACCAACACTGACACTGAGG

TGCGAAAGCGTGGGGAGCAAACAGGATTAGATACCCTGGTAGTCCACGCCGTAAACGATGTCAACTAGCC

GTTGGGACTCTTGAAGTCTTAGTGGCGCACCTAACGCACTAAGTTGACCGCCTGGGGAGTACGGCCGCAA

GGTTAAAACTCAAATGAATTGACGGGGGCCCGCACAAGCGGTGGAACATGTGGTTTAATTCGACGCAACG

CAAAAAACCTTACCTGGCCTTGACATGCTGAGAACTTTCCAGAGATGGATTGGTGCCTTCGGGAACTC

>PP575671.1 Pseudomonas syringae strain SHR02 16S ribosomal RNA gene, partial sequence

AGCGGCGGACGGGTGAGTAATGCCTAGGAATCTGCCTGGTAGTGGGGGATAACGCTCGGAAACGGACGCT

AATACCGCGTACGTCCTACGGGAGAAAGCAGGGGACCTTCGGGCCTTGCGCTATCAGATGAGCCTAGGTC

GGATTAGCTAGTTGGTGAGGTAATGGCTCACCAAGGCGACGATCCGTAACTGGTCTGAGAGGATGATCAG

TCACACTGGAACTGAGACACGGTCCAGACTCCTACGGGAGGCAGCAGTGGGGAATATTGGACAATGGGCG

AAAGCCTGATCCAGCCATGCCGCGTGTGTGAAGAAGGTCTTCGGATTGTAAAGCACTTTAAGTTGGGAGG

AAGGGCAGTTACCTAATACGTNTCTGTTTTGACGTTACCGACAGAATAAGCACCGGCTAACTCTGTGCCA

GCAGCCGCGGTAATACAGAGGGTGCAAGCGTTAATCGGAATTACTGGGCGTAAAGCGCGCGTAGGTGGTT

TGTTAAGGTGAATGTGAAATCCCCGGGCTCAACCTGGGAACTGCATCCAAAACTGGCAAGCTAGAGTATG

GTAGAGGGTGGTGGAATTTCCTGTGTAGCGGTGAAATGCGAAGATATAGGAAGGAACACCAGTGGCGAAG

GCGACCACCTGGACTGATACTGACACTGAGGTGCGAAAGCGTGGGGAGCAAACAGGATTAGATACCCTGG

TAGTCCACGCCGTAAACGATGTCAACTAGCCGTTGGGAGCCTTGAGCTCTTAGTGGCGCAGCTAACGCAT

TAAGTTGACCGCCTGGGGAGTACGGCCGCAAGGTTAAAACTCAAATGAATTGACGGGGGCCCGCACAAGC

GGTGGAGCATGTGGTTTAATTCGAAGCAACGCGAAGAACCTTACCAGGCCTTGACATCCAATGAACTTTC

CAGAGATGGATTGGTGCCTTCGGGAACATTGAGACAGGTGCTGCATGGCTGTCGTCAGCTCGTGTCGTGA

GATGTTGGGTTAAGTCCCGTAACGAGCGCAACCCTTGTCCTTAGTTACCAGCACGTAATGGTGGGCACTC

TAAGGAGACTGCCGGTGACAAACCGGAGGAAGGTGGGGATGACGTCAAGTCATCATGGCCCTTACGGCCT

GGGCTACACACGTGCTACAATGGTCGGTACAGAGGGTTGCCAAGCCGCGAGGTGGAGCTAATCTCACAAA

ACCGATCGTAGTCCGGATCGCACTCTGCAACTCGACTGCGTGAAGTCGGAAT
