# Supplementary material for: Molecular Identification of Culturable Bacteria and Vibrio Virulence‐Related Genes Associated With Sea Turtle Eggshells and Nest Sediments in Pangandaran, Indonesia
Source: Int J Microbiol. 2026 Oct 5;2026:7422673. doi: 10.1155/ijm/7422673 (PMC13639395; doi:10.1155/ijm/7422673)
Supplement: Supplementary file 2 [file IJM-2026-7422673-s002.zip › Hasil NCBI/Ds Consensus.docx]

>OR426156.1 Citrobacter sp. strain CPO 2.210 16S ribosomal RNA gene, partial sequence

CTCAGATTGAACGCTGGCGGCAGGCCTAACACATGCAAGTCGAACGGTAACAGGAAGCAGCTTGCTGCTT

TGCTGACGAGTGGCGGACGGGTGAGTAATGTCTGGGAAACTGCCCGATGGAGGGGGATAACTACTGGAAA

CGGTAGCTAATACCGCATAATGTCGCAAGACCAAAGAGGGGGACCTTCGGGCCTCTTGCCATCGGATGTG

CCCAGATGGGATTAGCTAGTTGGTGAGGTAACGGCTCACCAAGGCGACGATCCCTAGCTGGTCTGAGAGG

ATGACCAGCCACACTGGAACTGAGACACGGTCCAGACTCCTACGGGAGGCAGCAGTGGGGAATATTGCAC

AATGGGCGCAAGCCTGATGCAGCCATGCCGCGTGTATGAAGAAGGCCTTCGGGTTGTAAAGTACTTTCAG

CGGGGAGGAAGGGGTTAAGGTTAATAACCTTAGCCATTGACGTTACCCGCAGAAGAAGCACCGGCTAACT

CCGTGCCAGCAGCCGCGGTAATACGGAGGGTGCAAGCGTTAATCGGAATTACTGGGCGTAAAGCGCACGC

AGGCGGTCTGTCAAGTCGGATGTGAAATCCCCGGGCTCAACCTGGGAACTGCATTCGAAACTGGCAGGCT

TGAGTCTCGTAGAGGGGGGTAGAATTCCAGGTGTAGCGGTGAAATGCGTAGAGATCTGGAGGAATACCGG

TGGCGAAGGCGGCCCCCTGGACGAAGACTGACGCTCAGGTGCGAAAGCGTGGGGAGCAAACAGGATTAGA

TACCCTGGTAGTCCACGCCGTAAACGATGTCTATTTGGAGGTTGTGCCCTTGAGGCGTGGCTTCCGGAGC

TAACGCGTTAAATAGACCGCCTGGGGAGTACGGCCGCAAGGTTAAAACTCAAATGAATTGACGGGGGCCC

GCACAAGCGGTGGAGCATGTGGTTTAATTCGATGCAACGCGAAGAACCTTACCTGGTCTTGACATCCACA

GAACTTGGCAGAGATGCCTTGGTGCCTTCGGGAACTGTGAGACAGGTGCTGCATGGCTGTCGTCAGCTCG

TGTTGTGAAATGTTGGGTTAAGTCCCGCAACGAGCGCAACCCTTATCCTTTGTTGCCAGCGGTTAGGCCG

GGAACTCAAAGGAGACTGCCAGTGATAAACTGGAGGAAGGTGGGGATGACGTCAAGTCATCATGGCCCTT

ACGACCAGGGCTACACACGTGCTACAATGGCATATACAAAGAGAAGCGACCTCGCGAGAGCAAGCGGACC

TCATAAAGTATGTCGTAGTCCGGATTGGAGTCTGCAACTCGACTCCATGAAGTCGGAATCGCTAGTAATC

GTGGATCAGAATGCCACGGTGAATACGTTCCCGGGCCTTGTACACACCGCCCGTCACACCATGGGAGTGG

GTTGCAAAAGAAGTAGGTAGCTTAACCTTCGGGAGGGCGCTTACCACTTTGTGATTCATGACTGGGGTGA

A

>OR426156.1 Citrobacter sp. strain CPO 2.210 16S ribosomal RNA gene, partial sequence

CTCAGATTGAACGCTGGCGGCAGGCCTAACACATGCAAGTCGAACGGTAACAGGAAGCAGCTTGCTGCTT

TGCTGACGAGTGGCGGACGGGTGAGTAATGTCTGGGAAACTGCCCGATGGAGGGGGATAACTACTGGAAA

CGGTAGCTAATACCGCATAATGTCGCAAGACCAAAGAGGGGGACCTTCGGGCCTCTTGCCATCGGATGTG

CCCAGATGGGATTAGCTAGTTGGTGAGGTAACGGCTCACCAAGGCGACGATCCCTAGCTGGTCTGAGAGG

ATGACCAGCCACACTGGAACTGAGACACGGTCCAGACTCCTACGGGAGGCAGCAGTGGGGAATATTGCAC

AATGGGCGCAAGCCTGATGCAGCCATGCCGCGTGTATGAAGAAGGCCTTCGGGTTGTAAAGTACTTTCAG

CGGGGAGGAAGGGGTTAAGGTTAATAACCTTAGCCATTGACGTTACCCGCAGAAGAAGCACCGGCTAACT

CCGTGCCAGCAGCCGCGGTAATACGGAGGGTGCAAGCGTTAATCGGAATTACTGGGCGTAAAGCGCACGC

AGGCGGTCTGTCAAGTCGGATGTGAAATCCCCGGGCTCAACCTGGGAACTGCATTCGAAACTGGCAGGCT

TGAGTCTCGTAGAGGGGGGTAGAATTCCAGGTGTAGCGGTGAAATGCGTAGAGATCTGGAGGAATACCGG

TGGCGAAGGCGGCCCCCTGGACGAAGACTGACGCTCAGGTGCGAAAGCGTGGGGAGCAAACAGGATTAGA

TACCCTGGTAGTCCACGCCGTAAACGATGTCTATTTGGAGGTTGTGCCCTTGAGGCGTGGCTTCCGGAGC

TAACGCGTTAAATAGACCGCCTGGGGAGTACGGCCGCAAGGTTAAAACTCAAATGAATTGACGGGGGCCC

GCACAAGCGGTGGAGCATGTGGTTTAATTCGATGCAACGCGAAGAACCTTACCTGGTCTTGACATCCACA

GAACTTGGCAGAGATGCCTTGGTGCCTTCGGGAACTGTGAGACAGGTGCTGCATGGCTGTCGTCAGCTCG

TGTTGTGAAATGTTGGGTTAAGTCCCGCAACGAGCGCAACCCTTATCCTTTGTTGCCAGCGGTTAGGCCG

GGAACTCAAAGGAGACTGCCAGTGATAAACTGGAGGAAGGTGGGGATGACGTCAAGTCATCATGGCCCTT

ACGACCAGGGCTACACACGTGCTACAATGGCATATACAAAGAGAAGCGACCTCGCGAGAGCAAGCGGACC

TCATAAAGTATGTCGTAGTCCGGATTGGAGTCTGCAACTCGACTCCATGAAGTCGGAATCGCTAGTAATC

GTGGATCAGAATGCCACGGTGAATACGTTCCCGGGCCTTGTACACACCGCCCGTCACACCATGGGAGTGG

GTTGCAAAAGAAGTAGGTAGCTTAACCTTCGGGAGGGCGCTTACCACTTTGTGATTCATGACTGGGGTGA

A

>MH341951.1 Citrobacter sp. strain WA14-1-4 16S ribosomal RNA gene, partial sequence

GTCGAACGGTAACAGGAAGCAGCTTGCTGCTTTGCTGACGAGTGGCGGACGGGTGAGTAATGTCTGGGAA

ACTGCCCGATGGAGGGGGATAACTACTGGAAACGGTAGCTAATACCGCATAATGTCGCAAGACCAAAGAG

GGGGACCTTCGGGCCTCTTGCCATCGGATGTGCCCAGATGGGATTAGCTAGTTGGTGAGGTAACGGCTCA

CCAAGGCGACGATCCCTAGCTGGTCTGAGAGGATGACCAGCCACACTGGAACTGAGACACGGTCCAGACT

CCTACGGGAGGCAGCAGTGGGGAATATTGCACAATGGGCGCAAGCCTGATGCAGCCATGCCGCGTGTATG

AAGAAGGCCTTCGGGTTGTAAAGTACTTTCAGCGGGGAGGAAGGGGTTAAGGTTAATAACCTTAGCCATT

GACGTTACCCGCAGAAGAAGCACCGGCTAACTCCGTGCCAGCAGCCGCGGTAATACGGAGGGTGCAAGCG

TTAATCGGAATTACTGGGCGTAAAGCGCACGCAGGCGGTCTGTCAAGTCGGATGTGAAATCCCCGGGCTC

AACCTGGGAACTGCATTCGAAACTGGCAGGCTTGAGTCTCGTAGAGGGGGGTAGAATTCCAGGTGTAGCG

GTGAAATGCGTAGAGATCTGGAGGAATACCGGTGGCGAAGGCGGCCCCCTGGACGAAGACTGACGCTCAG

GTGCGAAAGCGTGGGGAGCAAACAGGATTAGATACCCTGGTAGTCCACGCCGTAAACGATGTCTATTTGG

AGGTTGTGCCCTTGAGGCGTGGCTTCCGGAGCTAACGCGTTAAATAGACCGCCTGGGGAGTACGGCCGCA

AGGTTAAAACTCAAATGAATTGACGGGGGCCCGCACAAGCGGTGGAGCATGTGGTTTAATTCGATGCAAC

GCGAAGAACCTTACCTGGTCTTGACATCCACAGAACTTGGCAGAGATGCCTTGGTGCCTTCGGGAACTGT

GAGACAGGTGCTGCATGGCTGTCGTCAGCTCGTGTTGTGAAATGTTGGGTTAAGTCCCGCAACGAGCGCA

ACCCTTATCCTTTGTTGCCAGCGGTTAGGCCGGGAACTCAAAGGAGACTGCCAGTGATAAACTGGAGGAA

GGTGGGGATGACGTCAAGTCATCATGGCCCTTACGACCAGGGCTACACACGTGCTACAATGGCATATACA

AAGAGAAGCGACCTCGCGAGAGCAAGCGGACCTCATAAAGTATGTCGTAGTCCGGATTGGAGTCTGCAAC

TCGACTCCATGAAGTCGGAATCGCTAGTAATCGTGGATCAGAATGCCACGGTGAATACGTTCCCGGGCCT

TGTACACACCGCCCGTCACACCATGGGAGTGGGTTGCAAAAGAAGTAGTTAGCTTAACCTTCGGGAGGGC

>GQ417434.1 Uncultured Citrobacter sp. clone F2aug.2 16S ribosomal RNA gene, partial sequence

ATTGAACGCTGGCGGCAGGCCTAACACATGCAAGTCGAACGGTAACAGGAAGCGGCTTGCTGCTTCGCTG

ACGAGTGGCGGACGGGTGAGTAATGTCTGGGAAACTGCCCGATGGAGGGGGATAACTACTGGAAACGGTA

GCTAATACCGCATAATGTCGCAAGACCAAAGAGGGGGACCTTCGGGCCTCTTGCCATCGGATGTGCCCAG

ATGGGATTAGCTAGTTGGTGAGGTAACGGCTCACCAAGGCGACGATCCCTAGCTGGTCTGAGAGGATGAC

CAGCCACACTGGAACTGAGACACGGTCCAGACTCCTACGGGAGGCAGCAGTGGGGAATATTGCACAATGG

GCGCAAGCCTGATGCAGCCATGCCGCGTGTATGAAGAAGGCCTTCGGGTTGTAAAGTACTTTCAGCGGGG

AGGAAGGGGTTAAGGTTAATAACCTTAGCCATTGACGTTACCCGCAGAAGAAGCACCGGCTAACTCCGTG

CCAGCAGCCGCGGTAATACGGAGGGTGCAAGCGTTAATCGGAATTACTGGGCGTAAAGCGCACGCAGGCG

GTCTGTCAAGTCGGATGTGAAATCCCCGGGCTCAACCTGGGAACTGCATTCGAAACTGGCAGGCTTGAGT

CTCGTAGAGGGGGGTAGAATTCCAGGTGTAGCGGTGAAATGCGTAGAGATCTGGAGGAATACCGGTGGCG

AAGGCGGCCCCCTGGACGAAGACTGACGCTCAGGTGCGAAAGCGTGGGGAGCAAACAGGATTAGATACCC

TGGTAGTCCACGCCGTAAACGATGTCTATTTGGAGGTTGTGCCCTTGAGGCGTGGCTTCCGGAGCTAACG

CGTTAAATAGACCGCCTGGGGAGTACGGCCGCAAGGTTAAAACTCAAATGAATTGACGGGGGCCCGCACA

AGCGGTGGAGCATGTGGTTTAATTCGATGCAACGCGAAGAACCTTACCTGGTCTTGACATCCACAGAACT

TGGCAGAGATGCCTTGGTGCCTTCGGGAACTGTGAGACAGGTGCTGCATGGCTGTCGTCAGCTCGTGTTG

TGAAATGTTGGGTTAAGTCCCGCAACGAGCGCAACCCTTATCCTTTGTTGCCAGCGGTTAGGCCGGGAAC

TCAAAGGAGACTGCCAGTGATAAACTGGAGGAAGGTGGGGATGACGTCAAGTCATCATGGCCCTTACGAC

CAGGGCTACACACGTGCTACAATGGCATATACAAAGAGAAGCGACCTCGCGAGAGCAAGCGGACCTCATA

AAGTATGTCGTAGTCCGGATTGGAGTCTGCAACTCGACTCCATGAAGTCGGAATCGCTAGTAATCGTGGA

TCAGAATGCCACGGTGAATACGTTCCCGGGCCTTGTACACACCGCCCGTCACACCATGGGAGTGGGTTGC

AAAAGAAGTAGGTAGCTTAACCTTCGGGAGGGCGCTTACCACTTTGTGATTCATGACTGGGGTGA

>MK104500.1 Citrobacter sp. strain 9B 16S ribosomal RNA gene, partial sequence

ATAATGTCGCAAGACCAAAGAGGGGGACCTTCGGGCCTCTTGCCATCGGATGTGCCCAGATGGGATTAGC

TAGTTGGTGAGGTAACGGCTCACCAAGGCGACGATCCCTAGCTGGTCTGAGAGGATGACCAGCCACACTG

GAACTGAGACACGGTCCAGACTCCTACGGGAGGCAGCAGTGGGGAATATTGCACAATGGGCGCAAGCCTG

ATGCAGCCATGCCGCGTGTATGAAGAAGGCCTTCGGGTTGTAAAGTACTTTCAGCGGGGAGGAAGGGGTT

AAGGTTAATAACCTTAGCCATTGACGTTACCCGCAGAAGAAGCACCGGCTAACTCCGTGCCAGCAGCCGC

GGTAATACGGAGGGTGCAAGCGTTAATCGGAATTACTGGGCGTAAAGCGCACGCAGGCGGTCTGTCAAGT

CGGATGTGAAATCCCCGGGCTCAACCTGGGAACTGCATTCGAAACTGGCAGGCTTGAGTCTCGTAGAGGG

GGGTAGAATTCCAGGTGTAGCGGTGAAATGCGTAGAGATCTGGAGGAATACCGGTGGCGAAGGCGGCCCC

CTGGACGAAGACTGACGCTCAGGTGCGAAAGCGTGGGGAGCAAACAGGATTAGATACCCTGGTAGTCCAC

GCCGTAAACGATGTCTATTTGGAGGTTGTGCCCTTGAGGCGTGGCTTCCGGAGCTAACGCGTTAAATAGA

CCGCCTGGGGAGTACGGCCGCAAGGTTAAAACTCAAATGAATTGACGGGGGCCCGCACAAGCGGTGGAGC

ATGTGGTTTAATTCGATGCAACGCGAAGAACCTTACCTGGTCTTGACATCCACAGAACTTGGCAGAGATG

CCTTGGTGCCTTCGGGAACTGTGAGACAGGTGCTGCATGGCTGTCGTCAGCTCGTGTTGTGAAATGTTGG

GTTAAGTCCCGCAACGAGCGCAACCCTTATCCTTTGTTGCCAGCGGTTAGGCCGGGAACTCAAAGGAGAC

TGCCAGTGATAAACTGGAGGAAGGTGGGGATGACGTCAAGTCATCATGGCCCTTACGACCAGGGCTACAC

ACGTGCTACAATGGCATATACAAAGAGAAGCGACCTCGCGAGAGCAAGCGGACCTCATAAAGTATGTCGT

AGTCCGGATTGGAGTCTGCAACTCGACTCCATGAAGTCGGAATCGCTAGTAATCGTGGATCAGAATGCCA

CGGTGAATACGTTCCCGGGCCTTGTACACACCGCCCGTCACACCATGGGAGTGGGTTGCAAAAGAAGTA
