# Supplementary material for: Molecular Identification of Culturable Bacteria and Vibrio Virulence‐Related Genes Associated With Sea Turtle Eggshells and Nest Sediments in Pangandaran, Indonesia
Source: Int J Microbiol. 2026 Oct 5;2026:7422673. doi: 10.1155/ijm/7422673 (PMC13639395; doi:10.1155/ijm/7422673)
Supplement: Supplementary file 2 [file IJM-2026-7422673-s002.zip › Hasil NCBI/AS_Consensus.docx]

>KT294091.1 Uncultured Klebsiella sp. clone M01598_122_000000000-ADV8A_1_1101_18039_17404 16S ribosomal RNA gene, partial sequence

CCTACGGGTGGCTGCAGTGGGGAATATTGCACAATGGGCGCAAGCCTGATGCAGCCATGCCGCGTGTGTG

AAGAAGGCCTTCGGGTTGTAAAGCACTTTCAGCGGGGAGGAAGGCGTTAAGGTTAATAACCTTGTCGATT

GACGTTACCCGCAGAAGAAGCACCGGCTAACTCCGTGCCAGCAGCCGCGGTAATACGGAGGGTGCAAGCG

TTAATCGGAATTACTGGGCGCAAAGCGCACGCAGGCGGTCTGTCAAGTCGGATGTGAAATCCCCGGGCTC

AACCTGGGAACTGCATTCGAAACTGGCAGGCTAGAGTCTTGTAGAGGGGGGTAGAATTCCAGGTGTAGCG

GTGAAATGCGTAGAGATCTGGAGGAATACCGGTGGCGAAGGCGGCCCCCTGGACAAAGACTGACGCTCAG

GTGCGAAAGCGTGGGGAGCAAACAGGATTAGATACCCGTGTAGTC

>GQ062442.1 Uncultured bacterium clone nbw32f04c1 16S ribosomal RNA gene, partial sequence

ATTGAACGCTGGCGGCAGGCCTAACACATGCAAGTCGAGCGGCAGCGGAAAGTAGCTTGCTACTTTGCCG

GCGAGCGGCGGACGGGTGAGTAATGTCTGGGAAACTGCCTGATGGAGGGGGATAACTACTGGAAACGGTA

GCTAATACCGCATAACGTCGCAAGACCAAAGAGGGGGACCTTCGGGCCTCTTGCCATCAGATGTGCCCAG

ATGGGATTAGCTAGTAGGTGGGGTAACGGCTCACCTAGGCGACGATCCCTAGCTGGTCTGAGAGGATGAC

CAGCCACACTGGAACTGAGACACGGTCCAGACTCCTACGGGAGGCAGCAGTGGGGAATATTGCACAATGG

GCGCAAGCCTGATGCAGCCATGCCGCGTGTATGAAGAAGGCCTTCGGGTTGTAAAGTACTTTCAGCGGGG

AGGAAGGCGATAAGGTTAATAACCTTGTCGATTGACGTTACCCGCAGAAGAAGCACCGGCTAACTCCGTG

CCAGCAGCCGCGGTAATACGGAGGGTGCAAGCGTTAATCGGAATTACTGGGCGCAAAGCGCACGCAGGCG

GTCTGTCAAGTCGGATGTGAAATCCCCGGGCTCAACCTGGGAACTGCATTCGAAACTGGCAGGCTAGAGT

CTTGTAGAGGGGGGTAGAATTCCAGGTGTAGCGGTGAAATGCGTAGAGATCTGGAGGAATACCGGTGGCG

AAGGCGGCCCCCTGGACAAAGACTGACGCTCAGGTGCGAAAGCGTGGGGAGCAAACAGGATTAGATACCC

TGGTAGTCCACGCCGTAAACGATGTCGACTTGGAGGTTGTGCCCTTGAGGCGTGGCTTCCGGAGCTAACG

CGTTAAGTCGACCGCCTGGGGAGTACGGCCGCAAGGTTAAAACTCAAATGAATTGACGGGGGCCCGCACA

AGCGGTGGAGCATGTGGTTTAATTCGATGCAACGCGAAGAACCTTACCTACTCTTGACATCCAGAGAACT

TTCCAGAGATGGATTGGTGCCTTCGGGAACTCTGAGACAGGTGCTGCATGGCTGTCGTCAGCTCGTGTTG

TGAAATGTTGGGTTAAGTCCCGCAACGAGCGCAACCCTTATCCTTTGTTGCCAGCGGTTCGGCCGGGAAC

TCAAAGGAGACTGCCAGTGATAAACTGGAGGAAGGTGGGGATGACGTCAAGTCATCATGGCCCTTACGAG

TAGGGCTACACACGTGCTACAATGGCGCATACAAAGAGAAGCGACCTCGCGAGAGCAAGCGGACCTCATA

AAGTGCGTCGTAGTCCGGATCGGAGTCTGCAACTCGACTCCATGAAGTCGGAATCGCTAGTAATCGTAGA

TCAGAATGCTACGGTGAAAACGTTCCCGGGCCT

>HQ756502.1 Uncultured organism clone ELU0039-T206-S-NIPCRAMgANa_000026 small subunit ribosomal RNA gene, partial sequence

TGGCGGCAGGCCTAACACATGCAAGTCGAGCGGTAGCACAGAGAGCTTGCCCTCGGGTGACGAGCGGCGG

ACGGGTGAGTAATGTCTGGGAAACTGCCTGATGGAGGGGGATAACTACTGGAAACGGTAGCTAATACCGC

ATAACGTCGCAAGACCAAAGTGGGGGACCTTCGGGCCTCATGCCATCAGATGTGCCCAGATGGGATTAGC

TAGTAGGTGGGGTAACGGCTCACCTAGGCGACGATCCCTAGCTGGTCTGAGAGGATGACCAGCCACACTG

GAACTGAGACACGGTCCAGACTCCTACGGGAGGCAGCAGTGGGGAATATTGCACAATGGGCGCAAGCCTG

ATGCAGCCATGCCGCGTGTGTGAAGAAGGCCTTCGGGTTGTAAAGCACTTTCAGCGGGGAGGAAGGCGTT

AAGGTTAATAACCTTGTCGATTGACGTTACCCGCAGAAGAAGCACCGGCTAACTCCGTGCCAGCAGCCGC

GGTAATACGGAGGGTGCAAGCGTTAATCGGAATTACTGGGCGCAAAGCGCACGCAGGCGGTCTGTCAAGT

CGGATGTGAAATCCCCGGGCTCAACCTGGGAACTGCATTCGAAACTGGCAGGCTAGAGTCTTGTAGAGGG

GGGTAGAATTCCAGGTGTAGCGGTGAAATGCGTAGAGATCTGGAGGAATACCGGTGGCGAAGGCGGCCCC

CTGGACAAAGACTGACGCTCAGGTGCGAAAGCGTGGGGAGCAAACAGGATTAGATACCCTGGTAGTCCAC

GCCGTAAACGATGTCGATTTGGAGGTTGTGCCCTTGAGGCGTGGCTTCCGGAGCTAACGCGTTAAATCGA

CCGCCTGGGGAGTACGGCCGCAAGGTTAAAACTCAAATGAATTGACGGGGGCCCGCACAAGCGGTGGAGC

ATGTGGTTTAATTCGATGCAACGCGAAGAACCTTACCTGGTCTTGACATCCACAGAACTTTCCAGAGATG

GATTGGTGCCTTCGGGAACTGTGAGACAGGTGCTGCATGGCTGTCGTCAGCTCGTGTTGTGAAATGTTGG

GTTAAGTCCCGCAACGAGCGCAACCCTTATCCTTTGTTGCCAGCGGTTAGGCCGGGAACTCAAAGGAGAC

TGCCAGTGATAAACTGGAGGAAGGTGGGGATGACGTCAAGTCATCATGGCCCTTACGACCAGGGCTACAC

ACGTGCTACAATGGCATATACAAAGAGAAGCGACCTCGCGAGAGCAAGCGGACCTCATAAAGTATGTCGT

AGTCCGGATTGGAGTCTGCAACTCGACTCCATGAAGTCGGAATCGCTAGTAATCGTAGATCAGAATGCTA

CGGTGAATACGTTCCCGGGCCTTGTACACACCGCCCGTCACACCATGGGAGTGGGTTGCAAAAGAAGTAG

GTAGCTTAACCTTCGGGAGGGCGCTTACCACTTTGTGATTCATGACTGGGGTG

>KX994496.1 Uncultured bacterium clone Otu04531 16S ribosomal RNA gene, partial sequence

CCTACGGGACGCACCAGTGGGGAATATTGCACAATGGGCGCAAGCCAGATGCAGCCATGCCGCGTGTGTG

AAGAAGGCCTTCGGGTTGTAAAGCACTTTCAGCGGGGAGGAAGGCGATAAGGTTAATAACCTTGTCGATT

GACGTTACCCGCAGAAGAAGCACCGGCTAACTCCGTGCCAGCAGCCGCGGTAATACGGAGGGTGCAAGCG

TTAATCGGAATTACTGGGCGCAAAGCGCACGCAGGCGGTCCGTCAAGTCGGATGTGAAATCCCCGGGCTC

AACCTGGGAACTGCATTCGAAACTGGCAGGCTAGAGTCTCGTAGAGGGGGGTAGAATTCCAGGTGTAGCG

GTGAAATGCGTAGAGATCTGGAGGAATACCGGTGGCGAAGGCGGCCCACTGGACGAAGACTGACGCTCAG

GTGCGAAAGCGTGGGGAGCAAACAGGATTAGATACCCCAGTAGTC

>KT300999.1 Uncultured Klebsiella sp. clone M01598_122_000000000-ADV8A_1_1104_23423_6008 16S ribosomal RNA gene, partial sequence

CCTACGGGTGGCTGCAGTGGGGAATATTGCACAATGGGCGCAAGCCTGATGCAGCCATGCCGCGTGTGTG

AAGAAGGCCTTCGGGTTGTAAAGCACTTTCAGCGGGGAGGAAGGCGTTAAGGTTAATAACCTTGTCGATT

GACGTTACCCGCAGAAGAAGCACCGGCTAACTCCGTGCCAGCAGCCGCGGTAATACGGAGGGTGCAAGCG

TTAATCGGAATTACTGGGCGCAAAGCGCACGCAGGCGGTCTGTCAAGTCGGATGTGAAATCCCCGGGCTC

AACCTGGGAACTGCATTCGAAACTGGCAGGCTAGAGTCTTGTAGAGGGGGGTAGAATTCCAGGTGTAGCG

GTGAAATGCGTAGAGATCTGGAGGAATACCGGTGGCGAAGGCGGCCCCCTGGACAAAGACTGACGCTCAG

GTGCGAAAGCGTGGGGAGCAAACAGGATTAGATACCCCAGTAGTC
