# Supplementary material for: Molecular Identification of Culturable Bacteria and Vibrio Virulence‐Related Genes Associated With Sea Turtle Eggshells and Nest Sediments in Pangandaran, Indonesia
Source: Int J Microbiol. 2026 Oct 5;2026:7422673. doi: 10.1155/ijm/7422673 (PMC13639395; doi:10.1155/ijm/7422673)
Supplement: Supplementary file 2 [file IJM-2026-7422673-s002.zip › Hasil NCBI/ES Consensus.docx]

>LK392351.1 Shewanella sp. A2 MF-2014 partial 16S rRNA gene, isolate A2

CGAGCGGCGGACGGGTGAGTAATGCCTAGGGATCTGCCCAGTCGAGGGGGATAACAGTTGGAAACGACTG

CTAATACCGCATACGCCCTACGGGGGAAAGGAGGGGACCTTCGGGCCTTCCGCGATTGGATGAACCTAGG

TGGGATTAGCTAGTTGGTGAGGTAATGGCTCACCAAGGCGACGATCCCTAGCTGTTCTGAGAGGATGATC

AGCCACACTGGGACTGAGACACGGCCCAGACTCCTACGGGAGGCAGCAGTGGGGAATATTGCACAATGGG

GGAAACCCTGATGCAGCCATGCCGCGTGTGTGAAGAAGGCCTTCGGGTTGTAAAGCACTTTCAGTAGGGA

GGAAAGGGTGTAACTTAATACGTTATATCTGTGACGTTACCTACAGAAGAAGGACCGGCTAACTCCGTGC

CAGCAGCCGCGGTAATACGGAGGGTCCGAGCGTTAATCGGAATTACTGGGCGTAAAGCGTGCGCAGGCGG

TTTGTTAAGCGAGATGTGAAAGCCCTGGGCTCAACCTAGGAATAGCATTTCGAACTGGCGAACTAGAGTC

TTGTAGAGGGGGGTAGAATTCCAGGTGTAGCGGTGAAATGCGTAGAGATCTGGAGGAATACCGGTGGCGA

AGGCGGCCCCCTGGACAAAGACTGACGCTCATGCACGAAAGCGTGGGGAGCAAACAGGATTATATACCCT

GGTAGTCCACGCCATAAACGATGTCTACTCGGAGTTTGGTGTCTTGAACACTGGGCTCTCAAGCTAACGC

ATTAAGTAGACCGCCTGGGGAGTACGGCCGCAAGGTTAAAACTCAAATGAATTGACGGGGGCCCGCACAA

GCGGTGGAGCATGTGGTTTAATTCGATGCAACCCGAAAAACCTTACCTACTCTCGACATCTCAAGAAGAC

GCTAGAGATGGATTGGTGCCTTCGGAAACTGCGAGACAGGTGCTGCATGTCTGTCGTCAGCTCGTGTTGT

GAAATGTTGGGTTAAGTCCCGCAACGAGCGCAACCCCTATCCTTATTTGCCAGCACGTAATGGTGGGAAC

TCTAGGGAGACTGCCGGTGATAAACCGGAGGAAGGTGGGGACGACGTCAAGTCATCATGGCCCTTACGAG

TAGGGCTACACACGTGCTACAATGGCGAGTACAGAGGGTTGCAAAGCCGCGAGGTGGAGCTAATCTCACA

AAGCTCGTCGTAGTCCGGATTGGAGTCTGCAACTCGACTCCATGAAGTCGGAATCGCTAGTAATCGTGGA

TCAGAATGCCACGGTGAATACGTTCCCGGGCCTTGTACACACCGCCCGTCACACCATGGGAGTGGGCTGC

AAAAGA

>MF893336.1 Serratia marcescens strain D1 16S ribosomal RNA gene, partial sequence

AGAGTTTGATCCTGGCTCAGATTGAACGCTGGCGGCAGGCTTAACAGATGCAAGTCGAGCGGTAGCACAG

GGGAGCTTGCTCCCCGGGTGACGAGCGGCGGACGGGTGAGTAATGTCTGGGAAACTGCCTGATGGAGGGG

GATAACTACTGGAAACGGTAGCTAATACCGCATAACGTCGCAAGACCAAAGAGGGGGACCTTCGGGCCTC

TTGCCATCAGATGTGCCCAGATGGGATTAGCTAGTAGGTGGGGTAATGGCTCACCTAGGCGACGATCCCT

AGCTGGTCTGAGAGGATGACCAGCCACACTGGAACTGAGACACGGTCCAGACTCCTACGGGAGGCAGCAG

TGGGGAATATTGCACAATGGGCGCAAGCCTGATGCAGCCATGCCGCGTGTGTGAAGAAGGCCTTCGGGTT

GTAAAGCACTTTCAGCGAGGAGGAAGGTGGTGAGCTTAATACGTTCATCAATTGACGTTACTCGCAGAAG

AAGCACCGGCTAACTCCGTGCCAGCAGCCGCGGTAATACGGAGGGTGCAAGCGTTAATCGGAATTACTGG

GCGTAAAGCGCACGCAGGCGGTTTGTTAAGTCAGATGTGAAATCCCCGGGCTCAACCTGGGAACTGCATT

TGAAACTGGCAAGCTAGAGTCTCGTAGAGGGGGGTAGAATTCCAGGTGTAGCGGTGAAATGCGTAGAGAT

CTGGAGGAATACCGGTGGCAAAGGCGGCCCCCTGGACAAAGACTGACGCTCAGGTGCAAAATCGTGGGGA

GCTAACGGGATTAGATACCCTGGTAGTCCACGCTGTAAACGATGTCGATTTGGAAGGTTGTGCCCTTGAG

GCCTGGCTTCCCGGAGCTAACGCGTTTAAATCGACCCTCCTGGGGGAGTACCGCCCGCAAGGTTAAAAAC

TCAAATGAATTTGACGGGGGGCCCCGCAAGCGGTGGAGCATGTGGTTTAATTCGATGCAGACGCGAAGAA

CCTTACCTACTCTTGACATCGAAAGAACTTTCCCTTCTTCTTTGGAGATCCCAGAAACTCTTTGACAGGT

GCTGCATGGCTGTCGTCAGCCCTTCCGGTGAAATCCTGGGTTAAGTCCGCAACGAGCGCAACCCTTATCC

TTGTTGCCAGCGGTTCGGCCGGGAACTCAAAGGAGACTGCCAGTGATAAACTGGAGGAAGGTGGGGATGA

CGTCAAGTCATCATGGCCCTTACGAGTAGGGCTACACACGTGCTACAATGGCGTATACAAAGAGAAGCGA

CCTCGCGAGAGCACAGCGGACCTCATAAAGTACGTCGTAGTCCGGATTGGAGTCTGCAACTCGACTCCAT

GAAGTCGGAATCGCTAGTAATCGTAGATCAGAATGCTACGGTGAATACGTTCCCGGGTCTTGTACACACC

GACCGTCACATCATGCGACTTTATTGCAAAAGAAGTAGGTAGCTTAACCTTCGGGAGGGCGCTTACCACT

TTGTGATTCATGACTGGGGTGAAGTCGTAACAAGGTAGCCGT

>DQ342407.1 Uncultured bacterium clone AD1-2F2C 16S ribosomal RNA gene, partial sequence

GNCTACACATGCAAGTCGAACGGTAACAGGAACACTTNCTGCTTCGCTGACGAGTGGCGGACGGGTGAGT

AATGTCTGGGAAACTGCCTGATGGAGGGGGATAACTACTGGAAACGGTAGCTAATACCGCATAACGTCAC

AAGACCAAAGAGGGGGACCTTCGGGCCTCTTGCCATCGGATGTGCCCAGATGGGATTAGCTAGTAGGTGG

GGTAACGGCTCACCTAGGCGACGATCCCTAGCTGGCCTGAGAGGATGACCAGCCACACTGGAACTGAGAC

ACGGTCCAGACTCCTACGGGAGGCAGCAGTGGGGAATATTGCACAATGGGCGCAAGCCTGATGCAGCCAT

GCCGCGTGTATGAAGAAGGCCTTCGGGTTGTAAAGTACTTTCAGCGGGGAGGAAGGCGATAAGGTTAATA

ACCTTGTCGATTGACGTTACCCGCAGAAGAAGCACCGGCTAACTCCGTGCCAGCAGCCGCGGTAATACGG

AGGGTGCAAGCGTTAATCGGAATTACTGGGCGTAAAGCGCACGCAGGCGGTCTGTCAAGTCGGATGTGAA

ATCCCTGGGCTCAACCTGGGAACTGCATTCGAAACTGGCAGGCTAGAGTCTTGTAGAGGGGGGTAGAATT

CCAGGTGTAGCGGTGAAATGCGTAGAGATCTGGAGGAATACCGGTGGCGAAGGCGGCCCCCTGGACAAAG

ACTGACGCTCAGGTGCGAAAGCGTGGGGAGCAAACAGGATTAGATACCCTGGTAGTCCACGCCGTAAACG

ATGTCGACTTGGAAGTTGTGCCCTTGAGGCGTGGCTTCCGGAGCTTACGCGTTAAGTCGACCGCCTGGGG

GAGTACCGCCCGCAAGGTTAAAAACTCAAATGAATTTGACGGGGGCCCCGCACAAGCGGTGGGAGCATGT

GGGTTTAATTCCGATGCAACGCGAAAAAACCTTACCTACTTCTTGA

>JX296062.1 Uncultured bacterium clone FB1_1A 16S ribosomal RNA gene, partial sequence

AGTGGGGAATATTGCACAATGGGGGAAACCCTGATGCAGCCATGCCGCGTGTGTGAAGAAGGCCTTCGGG

TTGTAAAGCACTTTCAGTAGGGAGGAAAGGTAATGGTTTAATACACCATTACTGTGACGTTACCTACAGA

AGAAGGACCGGCTAACTCCGTGCCAGCAGCCGCGGTAATACGGAGGGTCCGAGCGTTAATCGGAATTACT

GGGCGTAAAGCGTGCGCAGGCGGTTTGTTAAGCGAGATGTGAAAGCCCTGGGCTCAACCTAGGAATAGCA

TTTCGAACTGGCGAACTAGAGTCTTGTAGAGGGGGGTAGAATTCCAGGTGTAGCGGTGAAATGCGTAGAG

ATCTGGAGGAATACCGGTGGCGAAGGCGGCCCCCTGGACAAAGACTGACGCTCAGGCACGAAAGCGTGGG

GAGCAAACAGGATTAGATACCCTGGTAGTCCACGCCATAAACGATGTCTACTCGGAGTTTGGTGTCTTGA

ACACTGGGCTCTCAAGCTAACGCATTAAGTAGACCGCCTGGGGAGTACGGCCGCAAGGTTA
