# Supplementary material for: Molecular Identification of Culturable Bacteria and Vibrio Virulence‐Related Genes Associated With Sea Turtle Eggshells and Nest Sediments in Pangandaran, Indonesia
Source: Int J Microbiol. 2026 Oct 5;2026:7422673. doi: 10.1155/ijm/7422673 (PMC13639395; doi:10.1155/ijm/7422673)
Supplement: Supplementary file 2 [file IJM-2026-7422673-s002.zip › Hasil NCBI/OS Consensus .docx]

```
>OR965054.1 Vibrio alginolyticus strain CICC 10889 16S 16S ribosomal RNA gene, partial sequence
GGAAACGAGTTATCTGAACCTTCGGGGAACGATAACGGCGTCGAGCGGCGGACGGGTGAGTAATGCCTAG
GAAATTGCCCTGATGTGGGGGATAACCATTGGAAACGATGGCTAATACCGCATGATGCCTACGGGCCAAA
GAGGGGGACCTTCGGGCCTCTCGCGTCAGGATATGCCTAGGTGGGATTAGCTAGTTGGTGAGGTAAGGGC
TCACCAAGGCGACGATCCCTAGCTGGTCTGAGAGGATGATCAGCCACACTGGAACTGAGACACGGTCCAG
ACTCCTACGGGAGGCAGCAGTGGGGAATATTGCACAATGGGCGCAAGCCTGATGCAGCCATGCCGCGTGT
GTGAAGAAGGCCTTCGGGTTGTAAAGCACTTTCAGTCGTGAGGAAGGTAGTATAGTTAATAGCTGTATTA
TTTGACGTTAGCGACAGAAGAAGCACCGGCTAACTCCGTGCCAGCAGCCGCGGTAATACGGAGGGTGCGA
GCGTTAATCGGAATTACTGGGCGTAAAGCGCATGCAGGTGGTTTGTTAAGTCAGATGTGAAAGCCCGGGG
CTCAACCTCGGAATAGCATTTGAAACTGGCAGACTAGAGTACTGTAGAGGGGGGTAGAATTTCAGGTGTA
GCGGTGAAATGCGTAGAGATCTGAAGGAATACCGGTGGCGAAGGCGGCCCCCTGGACAGATACTGACACT
CAGATGCGAAAGCGTGGGGAGCAAACAGGATTAGATACCCTGGTAGTCCACGCCGTAAACGATGTCTACT
TGGAGGTTGTGGCCTTGAGCCGTGGCTTTCGGAGCTAACGCGTTAAGTAGACCGCCTGGGGAGTACGGTC
GCAAGATTAAAACTCAAATGAATTGACGGGGGCCCGCACAAGCGGTGGAGCATGTGGTTTAATTCGATGC
AACGCGAAGAACCTTACCTACTCTTGACATCCAGAGAACTTTCCAGAGATGGATTGGTGCCTTCGGGAAC
TCTGAGACAGGTGCTGCATGGCTGTCGTCAGCTCGTGTTGTGAAATGTTGGGTTAAGTCCCGCAACGAGC
GCAACCCTTATCCTTGTTTGCCAGCGAGTAATGTCGGGAACTCCAGGGAGACTGCCGGTGATAAACCGGA
GGAAGGTGGGGACGACGTCAAGTCATCATGGCCCTTACGAGTAGGGCTACACACGTGCTACAATGGCGCA
TACAGAGGGCGGCCAACTTGCGAAAGTGAGCGAATCCCAAAAAGTGCGTCGTAGTCCGGATTGGAGTCTG
CAACTCGACTCCATGAAGTCGGAATCGCTAGTAATCGTGGATCAGAATGCCACGGTGAATACGTTCCCGG
GCCTTGTACACACCGCCCGTCACACCATGGGAGTGGGCTGCAAAAGAAGTA

>CP082315.1 Vibrio alginolyticus strain ZLV3 chromosome 1, complete sequence
GTGTCATCTTCGCTTTGGCTGCAATGTTTGCAGCAGCTTCAAGAAGAGCTACCAGCTACAGAATTCAGCA
TGTGGGTTCGTCCGCTACAAGCGGAGCTCAATGACAATACTCTCACTCTATTCGCGCCAAACCGCTTTGT
GTTGGACTGGGTGCGTGATAAGTATCTGAACAATATTACTCGCTTACTGCAGGAATACTGCGGTGGCGAT
ATTCCTAACTTACGCTTTGAAGTGGGGAGCCGACCTGTCTCTGCTCCAAAACCAGCGCCGACACGTACGC
CTGCAGATGTGGCTGCCGAGTCATCTGCACCTGCGCAATTACAGGCGCGTAAGCCAGTACACAAAACCTG
GGATGACGACGCTCAAGCTATTGCGGCTATCAATCACCGCTCAAACATGAACCCAAAACACAAGTTCGAC
AACTTTGTTGAAGGTAAGTCGAACCAACTGGGTTTGGCTGCGGCGCGTCAGGTATCGGATAACCCAGGTG
CAGCTTACAACCCACTATTCCTATACGGCGGCACGGGCTTGGGTAAAACGCACTTGTTGCATGCGGTGGG
CAACGCGATTGTGGATAACAACCCGAACGCAAAAGTGGTCTACATGCACTCAGAACGTTTTGTGCAAGAC
ATGGTAAAAGCGCTGCAAAACAATGCGATTGAAGAATTCAAACGCTACTACCGCAGTGTGGATGCACTCC
TTATCGATGACATCCAATTCTTTGCTAATAAAGAACGTTCGCAAGAAGAATTCTTCCATACCTTCAACGC
ACTATTGGAAGGTAATCAGCAGATCATTCTAACGTCTGACCGTTATCCTAAAGAAATTAATGGCGTTGAA
GATCGTCTTAAATCTCGCTTTGGTTGGGGTCTTACTGTTGCGATCGAGCCGCCTGAGCTTGAAACGCGTG
TGGCGATCTTGATGAAAAAAGCTGAAGATCACCAAATCCACCTTGCTGATGAAGTGGCATTCTTCATTGC
CAAGCGTCTGCGTTCGAACGTTCGTGAGCTAGAAGGTGCATTGAACCGTGTTATCGCGAACGCGAACTTT
ACTGGTCGTCCAATCACGATTGATTTTGTGCGTGAAGCGCTGCGCGATCTGCTTGCTCTACAAGAGAAGC
TGGTGACGATCGATAACATTCAAAAGACCGTGGCGGAATACTACAAGATTAAAGTGGCGGACTTGCTGTC
TAAGCGTCGTTCTCGTTCTGTAGCGCGTCCTCGTCAGCTGGCTATGGCGTTAGCAAAAGAACTAACCAAC
CATAGCTTGCCTGAAATTGGCGATGCGTTTGGGGGCCGCGATCATACGACCGTACTTCATGCTTGTCGCA
AGATTGAGCAGCTGCGTGAAGAGAGCCACGACATTAAAGAAGATTACTCTAACTTGATTCGCACCCTGTC
TTCGTAATCGGTTAGAATAACGGTATTCTAAGCAAATCATTTATTGAGCTGACAGCGTAAAGAGCCAAGC
ATGAAATTTACCATTGAACGTAGTCATTTAATCAAACCGCTACAGCAGGTTTCGGGCGCTTTAGGTGGCC
GGCCAACCCTGCCAATCCTGGGTAACCTACTTCTTAAAGTAGAAGAGAACGTATTGTCGATGACGGCAAC
CGATCTTGAAGTAGAGTTAGTAAGCAAAGTGACGCTAGAAGGTGATTTTGAAGCGGGCAGCATTACCGTT
CCTTCGCGTAAGTTTCTCGATATCTGCCGTGGTTTACCGGACGATGCGATCATCACATTTGTATTAGAAG
GTGATCGTGTACAAGTTCGCTCTGGTCGCAGCCGTTTCTCGTTGGCAACGCTTCCTGCGAACGATTTCCC
TAATATTGAAGACTGGCAAAGCGAAGTGGAAGTATCACTGAGCCAAGCTGATTTACGTACGCTGATCGAC
AAAACTCAGTTCTCGATGGCGAATCAAGACGTTCGTTACTACCTCAACGGCATGTTGTTTGAAATTGACG
GCACCACGCTACGCAGCGTGGCGACGGACGGCCACCGTATGGCGGTATCGCAAACTCAGCTAGGTGCGGA
CTTTGCGCAGAAGCAAATCATCGTGCCACGCAAAGGTGTGCAAGAATTAGTAAAGTTGATGGATGCGCCA
GAGCAGCCCGTAGTACTTCAGATCGGTAGCTCCAATGTTCGTGCTGAGGTGAATAACTTTACTTTTACCT
CTAAGTTGGTTGATGGTCGTTTCCCTGACTACCGCCGTGTATTGCCTCAGAGCACCAATAAGACACTAAT
AGCAAGTTGTGACGAGTTGCGCCAAGCCTTCTCTCGTGCTGCGATTCTTTCCAACGAAAAATTCCGTGGC
GTGCGAGTGAATCTGGCTGATACTGAAATGCGCATAACCGCGAATAACCCAGAACAAGAAGAAGCGGAAG
AGATGCTTGATGTAACCTTTGAAGGTGACGCCATCGAAATCGGCTTTAACGTAAGCTATGTGCTTGATGT
TCTGAACACGTTGCGTTGTGAAAATGTACGTGTGTCGATGTCGGATGCTAACGCCAGCGCGTTGATTGAA
AATGCCGACGATGACAGTGCCATGTACGTGGTGATGCCAATTCGTCTATAAGCCAACTGAATGCCTCTTT
CTCGTCTCATCATTCAGCAGTTTCGAAACATTAAAGCCTGTGATATTGAGTTATCAGCAGGCTTTAACTT
TCTTATCGGGCCGAACGGCAGCGGTAAAACCAGTGTCTTAGAAGCGGTTTATCTGCTCGGGCATGGCCGC
TCATTCAAGAGCTCTCTCACCGGACGAGTGATACAAAATGAGTGTGACGAACTGTTTGTTCATGGTCGTT
TTTTGAACTCGGATCAATTTGAGCTACCTATCGGCATTAATAAGCAGCGTGATGGCTCAACAGAGGTTAA
AATAGGCGGTCAATCTGGACAAAAATTAGCGCAGTTGGCACAAGTTCTGCCTCTGCAGTTGATACACCCA
GAAGGGTTTGATTTACTGACAGATGGCCCTAAACATCGTCGTGCGTTTATTGATTGGGGCGTGTTTCATA

>LC745351.1 Vibrio sp. gene for 16S ribosomal RNA, partial sequence
TAACGGCGTCGAGCGGCGGACGGGTGAGTAATGCCTAGGAAATTGCCCTGATGTGGGGGATAACCATTGG
AAACGATGGCTAATACCGCATGATGCCTACGGGCCAAAGAGGGGGACCTTCGGGCCTCTCGCGTCAGGAT
ATGCCTAGGTGGGATTAGCTAGTTGGTGAGGTAAGGGCTCACCAAGGCGACGATCCCTAGCTGGTCTGAG
AGGATGATCAGCCACACTGGAACTGAGACACGGTCCAGACTCCTACGGGAGGCAGCAGTGGGGAATATTG
CACAATGGGCGCAAGCCTGATGCAGCCATGCCGCGTGTGTGAAGAAGGCCTTCGGGTTGTAAAGCACTTT
CAGTCGTGAGGAAGGTGGTGTAGTTAATAGCTGTATTATTTGACGTTAGCGACAGAAGAAGCACCGGCTA
ACTCCGTGCCAGCAGCCGCGGTAATACGGAGGGTGCGAGCGTTAATCGGAATTACTGGGCGTAAAGCGCA
TGCAGGTGGTTTGTTAAGTCAGATGTGAAAGCCCGGGGCTCAACCTCGGAATTGCATTTGAAACTGGCAG
ACTAGAGTACTGTAGAGGGGGGTAGAATTTCAGGTGTAGCGGTGAAATGCGTAGAGATCTGAAGGAATAC
CGGTGGCGAAGGCGGCCCCCTGGACAGATACTGACACTCAGATGCGAAAGCGTGGGGAGCAAACAGGATT
AGATACCCTGGTAGTCCACGCCGTAAACGATGTCTACTTGGAGGTTGTGGCCTTGAGCCGTGGCTTTCGG
AGCTAACGCGTTAAGTAGACCGCCTGGGGAGTACGGTCGCAAGATTAAAACTCAAATGAATTGACGGGGG
CCCGCACAAGCGGTGGAGCATGTGGTTTAATTCGATGCAACGCGAAGAACCTTACCTACTCTTGACATCC
AGAGAACTTTCCAGAGATGGATTGGTGCCTTCGGGAACTCTGAGACAGGTGCTGCATGGCTGTCGTCAGC
TCGTGTTGTGAAATGTTGGGTTAAGTCCCGCAACGAGCGCAACCCTTATCCTTGTTTGCCAGCGAGTAAT
GTCGGGAACTCCAGGGAGACTGCCGGTGATAAACCGGAGGAAGGTGGGGACGACGTCAAGTCATCATGGC
CCTTACGAGTAGGGCTACACACGTGCTACAATGGCGCATACAGAGGGCAGCCAACTTGCGAAAGTGAGCG
AATCCCAAAAAGTGCGTCGTAGTCCGGATTGGAGTCTGCAACTCGACTCCATGAAGTCGGAATCGCTAGT
AATCGTGGATCAGAATGCCACGGTGAATACGTTCCCGGGCCTTGTACACACCGCCCGTCACACCATGGGA
GTGGGCTGCAAAAGAAGTAGGTA

>GU726845.1 Vibrio alginolyticus strain 1306 16S ribosomal RNA gene, partial sequence
AGTCGAGCGGAACGAGTTATCTGAACCTTCGGGGAACGATAACGGCGTCGAGCGGCGGACGGGTGAGTAA
TGCCTAGGAAATTGCCCTGATGTGGGGGATAACCATTGGAAACGATGGCTAATACCGCATGATGCCTACG
GGCCAAAGAGGGGGACCTTCGGGCCTCTCGCGTCAGGATATGCCTAGGTGGGATTAGCTAGTTGGTGAGG
TAAGGGCTCACCAAGGCGACGATCCCTAGCTGGTCTGAGAGGATGATCAGCCACACTGGAACTGAGACAC
GGTCCAGACTCCTACGGGAGGCAGCAGTGGGGAATATTGCACAATGGGCGCAAGCCTGATGCAGCCATGC
CGCGTGTGTGAAGAAGGCCTTCGGGTTGTAAAGCACTTTCAGTCGTGAGGAAGGTGGTGTAGTTAATAGC
TGTATTATTTGACGTTAGCGACAGAAGAAGCACCGGCTAACTCCGTGCCAGCAGCCGCGGTAATACGGAG
GGTGCGAGCGTTAATCGGAATTACTGGGCGTAAAGCGCATGCAGGTGGTTTGTTAAGTCAGATGTGAAAG
CCCGGGGCTCAACCTCGGAATAGCATTTGAAACTGGCAGACTAGAGTACTGTAGAGGGGGGTAGAATTTC
AGGTGTAGCGGTGAAATGCGTAGAGATCTGAAGGAATACCGGTGGCGAAGGCGGCCCCCTGGACAGATAC
TGACACTCAGATGCGAAAGCGTGGGGAGCAAACAGGATTAGATACCCTGGTAGTCCACGCCGTAAACGAT
GTCTACTTGGAGGTTGTGGCCTTGAGCCGTGGCTTTCGGAGCTAACGCGTTAAGTAGACCGCCTGGGGAG
TACGGTCGCAAGATTAAAACTCAAATGAATTGACGGGGGCCCGCACAAGCGGTGGAGCATGTGGTTTAAT
TCGATGCAACGCGAAGAACCTTACCTACTCTTGACATCCAGAGAACTTTCCAGAGATGGATTGGTGCCTT
CGGGAACTCTGAGACAGGTGCTGCATGGCTGTCGTCAGCTCGTGTTGTGAAATGTTGGGTTAAGTCCCGC
AACGAGCGCAACCCTTATCCTTGTTTGCCAGCGAGTAATGTCGGGAACTCCAGGGAGACTGCCGGTGATA
AACCGGAGGAAGGTGGGGACGACGTCAAGTCATCATGGCCCTTACGAGTAGGGCTACACACGTGCTACAA
TGGCGCATACAGAGGGCGGCCAACTTGCGAAAGTGAGCGAATCCCAAAAAGTGCGTCGTAGTCCGGATTG
GAGTCTGCAACTCGACTCCATGAAGTCGGAATCGCTAGTAATCGTGGATCAGAATGCCACGGTGAATACG
TTCCCGGGCCTTGTACACACCGCCCGTCACACCATGGGAGTGGGCTGCAAAAGAAGTAGGTAGTTTAACC
TTCGGG

 >CP046787.1 Vibrio parahaemolyticus strain 2013V-1174 chromosome 1, complete sequence
AACAACGTTTGAAAATGGCGAATCTTATTTTAAGTATGGCACTGAGGAAGGAATAAAAAACCCCGGATGA
ATTCCAGGGTTTTTAGGTATCTCGCAAAGTTTAGAATAATCCAGCTAAGCGGTTGAGATCCGATTGGATT
GCACCAGCGGTCACCTCGCGTCCTGCGCCTGGGCCACGAATCACCAAAGGGTTGTCCTTGTACCATTTAC
TTTCAATCGCAAAAATGTTGTCGCATGGCAGCAAGTTAGCCAGCGCATGTTCGCGCGGTAATGCTTCTAC
GCCCACCGTCGCTTTGCCATTTTTCTCTAAGCGCGCCACATATCGAAGCACCTGCTCATCTCTTTGTGCT
TTGGTTAGACGCTCTTGAAGAATCTCACTGAGCAGTGCGCCATTATCAAAGAACTCATCCAAACTCAGAG
AGCGCAGCTCTTCAGGCACCAGTGATTCCACTTTTACGCTATCCGGTTCAATATCCAGACCAGACTCACG
CGCTAAGATAACCAGTTTACGCATGACATCCGAACCATCTAAATCAGCACGCGGATCCGGTTCTGTTAAT
CCTTGTTGCCACGCTAAATCGACCAACTCATTAAACGGCACCGAGCCATCAAACTGCTGGAACAGCCAAG
AAAGTGTTCCCGAGAAGATTCCGGATAGCGCCACAATCTCGTCACCACTTTCACGCAGATCTCGCACGGT
ATGGTTGATAGGTAATCCAGCCCCGACCGTCGCGTTGTAGAGCCAGTAACGACCGATTTTCGCGAATGCA
TCTTGCACTTGATGGTAATACTGACTATCGGCAGAGCCTGCAACTTTGTTAGCAGAAATTAAATGGATGC
CTTGCTGAGCGATATCAACATAACGTTGCGCCAACTCTTTGCTCGCGGTCACGTCCAATACCACAGCTTC
ATCATAACCTTGTAAATCGCCTAACCGACTCAGCCACGCCCCATCGTTCTCAATACTTTCATCATCAAAC
CTGTCGGCAACCGCCGCCGCATCGATACCCTTTTCATCAAACCAATATGTCTGGCTATCGACAACGGCAA
CTAAGTCAAAGCTCATACCGTGGCGTTTTTCGAGTTCTGTTTTCTGAGTCGCAAATAGATTTAACCAGCT
AGAACCGATATTGCCTTTGCCACATAACGCTACCGCTACGCGCTTTTGCGCTTGAAATAACTGGCTGTGA
ATCAACTGCACTAATGCTTCGGTATCGGTGCGACGCAGTACCGCAACTAGGCTTAAACCTGATTCAGTTT
CAGAAACAAATTCAACTGGTGCGTGCTTCAATTTTTGGTAAAAACCAAAACAATGGTTGGCATTCTTCGT
TACGCCTGCTCCGACAGCGGCCACAAGTGAATAGCCTTCTTTGAGTTTGATTTCAGCTTCAACCGCAAGA
TCTTGTAGATACTTCAGCGCACCTGTCGCAATCTCTGCGGTGTACGCTAAGCGTAATTTTTGCTGATCGG
CTTGGGCTTCGTAAGAGAGTGGCTCGAGTTGCGCACGTTGAAGAGACTTTAAAACATCACTTTGGGTTTT
GTTGAAGTCATGACCATGCCTAAAAGACAATTGAATCAACAATACTTCATCCAAAGAAGTGATGATTTTC
GCTCCACGCCCAGAAGCAAGTACACGCTCAATACGCGTTGAACCCGATTCAGGTTGATAACTGCACTTGA
GGCTTAAGTCCATCGTGCTTTGCGCGACAGGTTGTAACGTACGGCTATGCAAGACCGGAGCTGCAAGGCG
GGCCAGTTCACTCGCTTCGTCTAAGCGCAGTAAAGGCAACAGACATGCGTCCGACACCAAACGAGGATCC
GCACTGTAAACGCCAGCAACATCACTCCAAATGGTTACGGTTGTCACTTCCGCCAAAGCACCTATTACGG
TTGCGGAATAATCCGAGCCATTCCGCCCCAACAGTACGGTTTCTCCTGCCTCATTTTGCGCCATAAATCC
AGTAATGATGACGCGTTTGTGGCTGTGTTGCGCCAACGCCTCCTTAATCAGGGGATAAGAACGCGCACGG
```
