# Supplementary material for: Molecular Identification of Culturable Bacteria and Vibrio Virulence‐Related Genes Associated With Sea Turtle Eggshells and Nest Sediments in Pangandaran, Indonesia
Source: Int J Microbiol. 2026 Oct 5;2026:7422673. doi: 10.1155/ijm/7422673 (PMC13639395; doi:10.1155/ijm/7422673)
Supplement: Supplementary file 2 [file IJM-2026-7422673-s002.zip › Hasil NCBI/YS Consensus .docx]

>HQ108124.1 Citrobacter sp. enrichment culture clone VN_TX2-12 16S ribosomal RNA gene, partial sequence

AGAGTTTGATCATGGCTCAGATTGAACGCTGGCGGCAGGCCTAACACATGCAAGTCGAACGGTAACAGGA

AGCAGCTTGCTGCTTTGCTGACGAGTGGCGGACGGGTGAGTAATGTCTGGGAAACTGCCCGATGGAGGGG

GATAACTACTGGAAACGGTAGCTAATACCGCATAATGTCGCAAGACCAAAGAGGGGGACCTTCGGGCCTC

TTGCCATCGGATGTTCCCAGATGGGATTAGCTTGTTGGTGAGGTAACGGCTCACCAAGGCGACGATCCCC

AGCTGGTCTGAGAGGATGACCAGCCACACTGGAACTGAGACACGGTCCAGACTCCTACGGGAGGCAGCAG

TGGGGAATATTGCACAATGGGCGCAAGCCTGATGCAGCCATGCCGCGTGTATGAAGAAGGCCTTCGGGTT

GTAAAGTACTTTCAGCGGGGAGGAAGGGGTTAAGGTTAATAACCTTAGCCATTGACGTTACCCGCAGAAG

AAGCACCGGCTAACTCCGTGCCAGCAGCCGCGGTAATACGGAGGGTGCAAGCGTTAATCGGAATTACTGG

GCGTAAAGCGCACGCAGGCGGTCTGTCAAGTCGGATGTGAAATCCCCGGGCTCAACCTGGGAACTGCATT

CGAAACTGGCAGGCTTGAGTCTCGTAGAGGGGGGTAGAATTCCAGGTGTAGCGGTGAAATGCGTAGAGAT

CTGGAGGAATACCGGTGGCGAAGGCGGCCCCCTGGACGAAGACTGACGCTCAGGTGCGAAAGCGTGGGGA

GCAAACAGGATTAGATACCCTGGTAGTCCACGCCGTAAACGATGTCTATTTGGAGGTTGTGCCCTTGAGG

CGTGGCTTCCGGAGCTAACGCGTTAAATAGACCGCCTGGGGAGTACGGCCGCAAGGTTAAAACTCAAATG

AATTGACGGGGGCCCGCACAAGCGGTGGAGCATGTGGTTTAATTCGATGCAACGCGAAGAACCTTACCTG

GTCTTGACATCCACAGAACTTGGCAGAGATGCCTTGGTGCCTTCGGGAACTGTGAGACAGGTGCTGCATG

GCTGTCGTCAGCTCGTGTTGTGAAATGTTGGGTTAAGTCCCGCAACGAGCGCAACCCTTATCCTTTGTTG

CCAGCGGTTAGGCCGGGAACTCAAAGGAGACTGCCAGTGATAAACTGGAGGAAGGTGGGGATGACGTCAA

GTCATCATGGCCCTTACGACCAGGGCTACACACGTGCTACAATGGCATATACAAAGAGAAGCGACCTCGC

GAGAGCAAGCGGACCTCATAAAGTATGTCGTAGTCCGGATTGGAGTCTGCAACTCGACTCCATGAAGTCG

GAATCGCTAGCAATCGTGGATCAGAATGCCACGGTGAATACGTTCCCGGGCCTTGTACACACCGCCCGTC

ACACCATGGGAGTGGGTTGCAAAAGAAGTAGGTAGCTTAACCTTCGGGAGGGCGCTTACCACTTTGTGAT

TCATGACTGGGGTGAAGTCGTAACAAGGTAACCGTA

>OQ198441.1 Citrobacter amalonaticus strain S3 16S ribosomal RNA gene, partial sequence

AGGCTCAGATTGAACGCTGGCGGCAGGCCTAACACATGCAAGTCGAACGGTAACAGGAAGCAGCTTGCTG

CTTTGCTGACGAGTGGCGGACGGGTGAGTAATGTCTGGGAAACTGCCCGATGGAGGGGGATAACTACTGG

AAACGGTAGCTAATACCGCATAATGTCGCAAGACCAAAGAGGGGGACCTTCGGGCCTCTTGCCATCGGAT

GTGCCCAGATGGGATTAGCTTGTTGGTGAGGTAACGGCTCACCAAGGCGACGATCCCTAGCTGGTCTGAG

AGGATGACCAGCCACACTGGAACTGAGACACGGTCCAGACTCCTACGGGAGGCAGCAGTGGGGAATATTG

CACAATGGGCGCAAGCCTGATGCAGCCATGCCGCGTGTATGAAGAAGGCCTTCGGGTTGTAAAGTACTTT

CAGCGGGGAGGAAGGGGTTAAGGTTAATAACCTTAGCCATTGACGTTACCCGCAGAAGAAGCACCGGCTA

ACTCCGTGCCAGCAGCCGCGGTAATACGGAGGGTGCAAGCGTTAATCGGAATTACTGGGCGTAAAGCGCA

CGCAGGCGGTCTGTCAAGTCGGATGTGAAATCCCCGGGCTCAACCTGGGAACTGCATTCGAAACTGGCAG

GCTTGAGTCTCGTAGAGGGGGGTAGAATTCCAGGTGTAGCGGTGAAATGCGTAGAGATCTGGAGGAATAC

CGGTGGCGAAGGCGGCCCCCTGGACGAAGACTGACGCTCAGGTGCGAAAGCGTGGGGAGCAAACAGGATT

AGATACCCTGGTAGTCCACGCCGTAAACGATGTCTATTTGGAGGTTGTGCCCTTGAGGCGTGGCTTCCGG

AGCTAACGCGTTAAATAGACCGCCTGGGGAGTACGGCCGCAAGGTTAAAACTCAAATGAATTGACGGGGG

CCCGCACAAGCGGTGGAGCATGTGGTTTAATTCGATGCAACGCGAAGAACCTTACCTGGTCTTGACATCC

ACAGAACTTGGCAGAGATGCCTTGGTGCCTTCGGGAACTGTGAGACAGGTGCTGCATGGCTGTCGTCAGC

TCGTGTTGTGAAATGTTGGGTTAAGTCCCGCAACGAGCGCAACCCTTATCCTTTGTTGCCAGCGGTTAGG

CCGGGAACTCAAAGGAGACTGCCAGTGATAAACTGGAGGAAGGTGGGGATGACGTCAAGTCATCATGGCC

CTTACGACCAGGGCTACACACGTGCTACAATGGCATATACAAAGAGAAGCGACCTCGCGAGAGCAAGCGG

ACCTCATAAAGTATGTCGTAGTCCGGATTGGAGTCTGCAACTCGACTCCATGAAGTCGGAATCGCTAGTA

ATCGTGGATCAGAATGCCACGGTGAATACGTTCCCGGGCCTTGTACACACCGCCCGTCACACCATGGGAG

TGGGTTGCAAAAGAAGTAGGTAGCTTAACCTTCGGGAGGGCGCTTACCACTTTGTGATTCATGACTGGGG

TGAAGTCGTAA

>JQ912533.1 Citrobacter sp. HD4.9 16S ribosomal RNA gene, partial sequence

GCTTGCTGCTTTGCTGACGAGTGGCGGACGGGTGAGTAATGTCTGGGAAACTGCCCGATGGAGGGGGATA

ACTACTGGAAACGGTAGCTAATACCGCATAATGTCGCAAGACCAAAGAGGGGGACCTTCGGGCCTCTTGC

CATCGGATGTGCCCAGATGGGATTAGCTAGTTGGTGAGGTAACGGCTCACCAAGGCGACGATCCCTAGCT

GGTCTGAGAGGATGACCAGCCACACTGGAACTGAGACACGGTCCAGACTCCTACGGGAGGCAGCAGTGGG

GAATATTGCACAATGGGCGCAAGCCTGATGCAGCCATGCCGCGTGTATGAAGAAGGCCTTCGGGTTGTAA

AGTACTTTCAGCGGGGAGGAAGGGGTTAAGGTTAATAACCTTAGCCATTGACGTTACCCGCAGAAGAAGC

ACCGGCTAACTCCGTGCCAGCAGCCGCGGTAATACGGAGGGTGCAAGCGTTAATCGGAATTACTGGGCGT

AAAGCGCACGCAGGCGGTCTGTCAAGTCGGATGTGAAATCCCCGGGCTCAACCTGGGAACTGCATTCGAA

ACTGGCAGGCTTGAGTCTCGTAGAGGGGGGTAGAATTCCAGGTGTAGCGGTGAAATGCGTAGAGATCTGG

AGGAATACCGGTGGCGAAGGCGGCCCCCTGGACGAAGACTGACGCTCAGGTGCGAAAGCGTGGGGAGCAA

ACAGGATTAGATACCCTGGTAGTCCACGCCGTAAACGATGTCTATTTGGAGGTTGTGCCCTTGAGGCGTG

GCTTCCGGAGCTAACGCGTTAAATAGACCGCCTGGGGAGTACGGCCGCAAGGTTAAAACTCAAATGAATT

GACGGGGGCCCGCACAAGCGGTGGAGCATGTGGTTTAATTCGATGCAACGCGAAGAACCTTACCTGGTCT

TGACATCCACAGAACTTGGCAGAGATGCCTTGGTGCCTTCGGGAACTGTGAGACAGGTGCTGCATGGCTG

TCGTCAGCTCGTGTTGTGAAATGTTGGGTTAAGTCCCGCAACGAGCGCAACCCTTATCCTTTGTTGCCAG

CGGTTAGGCCGGGAACTCAAAGGAGACTGCCAGTGATAAACTGGAGGAAGGTGGGGATGACGTCAAGTCA

TCATGGCCCTTACGACCAGGGCTACACACGTGCTACAATGGCATATACAAAGAGAAGCGACCTCGCGAGA

GCAAGCGGACCTCATAAAGTATGTCGTAGTCCGGATTGGAGTCTGCAACTCGACTCCATGAAGTCGGAAT

CGCTAGTAATCGTGGATCAGAATGC

>OR426156.1 Citrobacter sp. strain CPO 2.210 16S ribosomal RNA gene, partial sequence

CTCAGATTGAACGCTGGCGGCAGGCCTAACACATGCAAGTCGAACGGTAACAGGAAGCAGCTTGCTGCTT

TGCTGACGAGTGGCGGACGGGTGAGTAATGTCTGGGAAACTGCCCGATGGAGGGGGATAACTACTGGAAA

CGGTAGCTAATACCGCATAATGTCGCAAGACCAAAGAGGGGGACCTTCGGGCCTCTTGCCATCGGATGTG

CCCAGATGGGATTAGCTAGTTGGTGAGGTAACGGCTCACCAAGGCGACGATCCCTAGCTGGTCTGAGAGG

ATGACCAGCCACACTGGAACTGAGACACGGTCCAGACTCCTACGGGAGGCAGCAGTGGGGAATATTGCAC

AATGGGCGCAAGCCTGATGCAGCCATGCCGCGTGTATGAAGAAGGCCTTCGGGTTGTAAAGTACTTTCAG

CGGGGAGGAAGGGGTTAAGGTTAATAACCTTAGCCATTGACGTTACCCGCAGAAGAAGCACCGGCTAACT

CCGTGCCAGCAGCCGCGGTAATACGGAGGGTGCAAGCGTTAATCGGAATTACTGGGCGTAAAGCGCACGC

AGGCGGTCTGTCAAGTCGGATGTGAAATCCCCGGGCTCAACCTGGGAACTGCATTCGAAACTGGCAGGCT

TGAGTCTCGTAGAGGGGGGTAGAATTCCAGGTGTAGCGGTGAAATGCGTAGAGATCTGGAGGAATACCGG

TGGCGAAGGCGGCCCCCTGGACGAAGACTGACGCTCAGGTGCGAAAGCGTGGGGAGCAAACAGGATTAGA

TACCCTGGTAGTCCACGCCGTAAACGATGTCTATTTGGAGGTTGTGCCCTTGAGGCGTGGCTTCCGGAGC

TAACGCGTTAAATAGACCGCCTGGGGAGTACGGCCGCAAGGTTAAAACTCAAATGAATTGACGGGGGCCC

GCACAAGCGGTGGAGCATGTGGTTTAATTCGATGCAACGCGAAGAACCTTACCTGGTCTTGACATCCACA

GAACTTGGCAGAGATGCCTTGGTGCCTTCGGGAACTGTGAGACAGGTGCTGCATGGCTGTCGTCAGCTCG

TGTTGTGAAATGTTGGGTTAAGTCCCGCAACGAGCGCAACCCTTATCCTTTGTTGCCAGCGGTTAGGCCG

GGAACTCAAAGGAGACTGCCAGTGATAAACTGGAGGAAGGTGGGGATGACGTCAAGTCATCATGGCCCTT

ACGACCAGGGCTACACACGTGCTACAATGGCATATACAAAGAGAAGCGACCTCGCGAGAGCAAGCGGACC

TCATAAAGTATGTCGTAGTCCGGATTGGAGTCTGCAACTCGACTCCATGAAGTCGGAATCGCTAGTAATC

GTGGATCAGAATGCCACGGTGAATACGTTCCCGGGCCTTGTACACACCGCCCGTCACACCATGGGAGTGG

GTTGCAAAAGAAGTAGGTAGCTTAACCTTCGGGAGGGCGCTTACCACTTTGTGATTCATGACTGGGGTGA

A

>OQ780950.1 Citrobacter amalonaticus strain BASG52 16S ribosomal RNA gene, partial sequence

TTAGAGTTTTGGTATCTGGCTCAGATTGAACGCTGGCGGCAGGCCTAACACATGCAAGTCGAACGGTAAC

AGGAAGCAGCTTGCTGCTTTGCTGACGAGTGGCGGACGGGTGAGTAATGTCTGGGAAACTGCCCGATGGA

GGGGGATAACTACTGGAAACGGTAGCTAATACCGCATAATGTCGCAAGACCAAAGAGGGGGACCTTCGGG

CCTCTTGCCATCGGATGTGCCCAGATGGGATTAGCTAGTTGGTGAGGTAACGGCTCACCAAGGCGACGAT

CCCTAGCTGGTCTGAGAGGATGACCAGCCACACTGGAACTGAGACACGGTCCAGACTCCTACGGGAGGCA

GCAGTGGGGAATATTGCACAATGGGCGCAAGCCTGATGCAGCCATGCCGCGTGTATGAAGAAGGCCTTCG

GGTTGTAAAGTACTTTCAGCGGGGAGGAAGGGGTTAAGGTTAATAACCTTAGCCATTGACGTTACCCGCA

GAAGAAGCACCGGCTAACTCCGTGCCAGCAGCCGCGGTAATACGGAGGGTGCAAGCGTTAATCGGAATTA

CTGGGCGTAAAGCGCACGCAGGCGGTCTGTCAAGTCGGATGTGAAATCCCCGGGCTCAACCTGGGAACTG

CATTCGAAACTGGCAGGCTTGAGTCTCGTAGAGGGGGGTAGAATTCCAGGTGTAGCGGTGAAATGCGTAG

AGATCTGGAGGAATACCGGTGGCGAAGGCGGCCCCCTGGACGAAGACTGACGCTCAGGTGCGAAAGCGTG

GGGAGCAAACAGGATTAGATACCCTGGTAGTCCACGCCGTAAACGATGTCTATTTGGAGGTTGTGCCCTT

GAGGCGTGGCTTCCGGAGCTAACGCGTTAAATAGACCGCCTGGGGAGTACGGCCGCAAGGTTAAAACTCA

AATGAATTGACGGGGGCCCGCACAAGCGGTGGAGCATGTGGTTTAATTCGATGCAACGCGAAGAACCTTA

CCTGGTCTTGACATCCACAGAACTTGGCAGAGATGCCTTGGTGCCTTCGGGAACTGTGAGACAGGTGCTG

CATGGCTGTCGTCAGCTCGTGTTGTGAAATGTTGGGTTAAGTCCCGCAACGAGCGCAACCCTTATCCTTT

GTTGCCAGCGGTTAGGCCGGGAACTCAAAGGAGACTGCCAGTGATAAACTGGAGGAAGGTGGGGATGACG

TCAAGTCATCATGGCCCTTACGACCAGGGCTACACACGTGCTACAATGGCATATACAAAGAGAAGCGACC

TCGCGAGAGCAAGCGGACCTCATAAAGTATGTCGTAGTCCGGATTGGAGTCTGCAACTCGACTCCATGAA

GTCGGAATCGCTAGTAATCGTGGATCAGAATGCCACGGTGAATACGTTCCCGGGCCTTGTACACACCGCC

CGTCACACCATGGGAGTGGGTTGCAAAAGAAGTAGGTAGCTTAACCTTCGGGAGGGCGCTTACCACTTTG

TGATTCATGACTGGGGTGAAGTCTA
