# Supplementary material for: Molecular Identification of Culturable Bacteria and Vibrio Virulence‐Related Genes Associated With Sea Turtle Eggshells and Nest Sediments in Pangandaran, Indonesia
Source: Int J Microbiol. 2026 Oct 5;2026:7422673. doi: 10.1155/ijm/7422673 (PMC13639395; doi:10.1155/ijm/7422673)
Supplement: Supplementary file 2 [file IJM-2026-7422673-s002.zip › Hasil NCBI/QS Cosensus.docx]

>LR218095.1 Bacillus halmapalus partial 16S rRNA gene, isolate IK_P55

GATGAACGCTGGCGGCGTGCCTAATACATGCAAGTCGAGCGGACTTACAAAAGCTTGCTTTTGTAAGTTA

GCGGCGGACGGGTGAGTAACACGTGGGCAACCTGCCCTCGAGACTGGGATAACTTCGGGAAACCGGAGCT

AATACCGGATAATATAAGGAACCTCCTGGTTCCTTRTTGAAAGATGGTTTCGGCTATCACTTGAGGATGG

GCCCGCGGCGCATTAGCTAGTTGGTGAGGTAACGGCTCACCAAGGCGACGATGCGTAGCCGACCTGAGAG

GGTGATCGGCCACACTGGGACTGAGACACGGCCCAGACTCCTACGGGAGGCAGCAGTAGGGAATCTTCCA

CAATGGACGAAAGTCTGATGGAGCAACGCCGCGTGAGTGATGAAGGCCTTCGGGTCGTAAAGCTCTGTTG

TTAGGGAAGAACAAGTGCGAGAGTAACTGCTCGCACCTTGACGGTACCTAACCAGAAAGCCACGGCTAAC

TACGTGCCAGCAGCCGCGGTAATACGTAGGTGGCAAGCGTTATCCGGAATTATTGGGCGTAAAGCGCGCG

CAGGTGGTTTCTTAAGTCTGATGTGAAAGCCCACGGCTCAACCGTGGAGGGTCATTGGAAACTGGGGAAC

TTGAGTGCAGAAGAGGAAAGTGGAATTCCAAGTGTAGCGGTGAAATGCGTAGAGATTTGGAGGAACACCA

GTGGCGAAGGCGACTTTCTGGTCTGTAACTGACACTGAGGCGCGAAAGCGTGGGGAGCAAACAGGATTAG

ATACCCTGGTAGTCCACGCCGTAAACGATGAGTGCTAAGTGTTAGAGGGTTTCCGCCCTTTAGTGCTGCA

GCTAACGCATTAAGCACTCCGCCTGGGGAGTACGGTCGCAAGACTGAAACTCAAAGGAATTGACGGGGGC

CCGCACAAGCGGTGGAGCATGTGGTTTAATTCGAAGCAACGCGAAGAACCTTACCAGGTCTTGACATCCT

CTGACAACCCTAGAGATAGGGCGTTCCCCTTCGGGGGACAGAGTGACAGGTGGTGCATGGTTGTCGTCAG

CTCGTGTCGTGAGATGTTGGGTTAAGTCCCGCAACGAGCGCAACCCTTGATCTTAGTTGCCAGCATTCAG

TTGGGCACTCTAAGGTGACTGCCGGTGACAAACCGGAGGAAGGTGGGGATGACGTCAAATCATCATGCCC

CTTATGACCTGGGCTACACACGTGCTACAATGGACGGTACAAAGGGCAGCAAAACCGCGAGGTCGAGCCA

ATCCCATAAAACCGTTCTCAGTTCGGATTGCAGGCTGCAACTCGCCTGCATGAAGCCGGAATCGCTAGTA

ATCGCGGATCAGCATGCCGCGGTGAATACGTTCCCGGGCCTTGTACACACCGCCCGTCACACCACGAGAG

TTTGTAACACCCGAAGTCGGTGGGGTAACCTTTGGAGCCAGCCGCC

>KU955637.1 Sutcliffiella halmapala strain FJAT-44660 16S ribosomal RNA gene, partial sequence

GCAGTCGAGCGGACTTACAAAAGCTTGCTTTTGTAAGTTAGCGGCGGACGGGTGAGTAACACGTGGGCAA

CCTGCCCTTGAGACTGGGATAACTTCGGGAAACCGGAGCTAATACCGGATAATATGAGGAACCTCCTGGT

TCCTTATTGAAAGATGGTTTCGGCTATCACTTAAGGATGGGCCCGCGGCGCATTAGCTAGTTGGTGAGGT

AACGGCTCACCAAGGCGACGATGCGTAGCCGACCTGAGAGGGTGATCGGCCACACTGGGACTGAGACACG

GCCCAGACTCCTACGGGAGGCAGCAGTAGGGAATCTTCCACAATGGACGAAAGTCTGATGGAGCAACGCC

GCGTGAGTGATGAAGGCCTTCGGGTCGTAAAGCTCTGTTGTTAGGGAAGAACAAGTGCGAGAGTAACTGC

TCGCACCTTGACGGTACCTAACCAGAAAGCCACGGCTAACTACGTGCCAGCAGCCGCGGTAATACGTAGG

TGGCAAGCGTTATCCGGAATTATTGGGCGTAAAGCGCGCGCAGGTGGTTTCTTAAGTCTGATGTGAAAGC

CCACGGCTCAACCGTGGAGGGTCATTGGAAACTGGGGAACTTGAGTGCAGAAGAGGAAAGTGGAATTCCA

AGTGTAGCGGTGAAATGCGTAGAGATTTGGAGGAACACCAGTGGCGAAGGCGACTTTCTGGTCTGTAACT

GACACTGAGGCGCGAAAGCGTGGGGAGCAAACAGGATTAGATACCCTGGTAGTCCACGCCGTAAACGATG

AGTGCTAAGTGTTAGAGGGTTTCCGCCCTTTAGTGCTGCAGCTAACGCATTAAGCACTCCGCCTGGGGAG

TACGGTCGCAAGACTGAAACTCAAAGGAATTGACGGGGGCCCGCACAAGCGGTGGAGCATGTGGTTTAAT

TCGAAGCAACGCGAAGAACCTTACCAGGTCTTGACATCCTCTGACAACCCTAGAGATAGGGCGTTCCCCT

TCGGGGGACAGAGTGACAGGTGGTGCATGGTTGTCGTCAGCTCGTGTCGTGAGATGTTGGGTTAAGTCCC

GCAACGAGCGCAACCCTTGATCTTAGTTGCCAGCATTCAGTTGGGCACTCTAAGGTGACTGCCGGTGACA

AACCGGAGGAAGGTGGGGATGACGTCAAATCATCATGCCCCTTATGACCTGGGCTACACACGTGCTACAA

TGGACGGTACAAAGGGCAGCAAAACCGCGAGGTCGAGCCAATCCCATAAAACCGTTCTCAGTTCGGATTG

CAGGCTGCAACTCGCCTGCATGAAGCCGGAATCGCTAGTAATCGCGGATCAGCATGCCGCGGTGAATACG

TTCCCGGGCCTTGTACACACCGCCCGTCACACCACGAGAGTTTGTAACACCCGAAGTCGGTGGGGTAACC

TTTGGAGCC

>AF479350.1 Glacial ice bacterium G200-T16 16S ribosomal RNA gene, partial sequence

TGAACGCTGGCGGCGTGCCTAATACATGCAAGTCGAGCGGACTTACAAAAGCTTGCTTTTGTAAGTTAGC

GGCGGACGGGTGAGTAACACGTGGGCAACCTGCCCTTGAGACTGGGATAACTTCGGGAAACCGGAGCTAA

TACCGGATAATATGAGGAACCTCCTGGTTCCTTATTGAAAGATGGTTTCGGCTATCACTTAAGGATGGGC

CCGCGGCGCATTAGCTAGTTGGTGAGGTAACGGCTCACCAAGGCGACGATGCGTAGCCGACCTGAGAGGG

TGATCGGCCACACTGGGACTGAGACACGGCCCAGACTCCTACGGGAGGCAGCAGTAGGGAATCTTCCACA

ATGGACGAAAGTCTGATGGAGCAACGCCGCGTGAGTGATGAAGGCCTTCGGGTCGTAAAGCTCTGTTGTT

AGGGAAGAACAAGTGCGAGAGTAACTGCTCGCACCTTGACGGTACCTAACCAGAAAGCCACGGCTAACTA

CGTGCCAGCAGCCGCGGTAATACGTAGGTGGCAAGCGTTATCCGGAATTATTGGGCGTAAAGCGCGCGCA

GGTGGTTTCTTAAGTCTGATGTGAAAGCCCACGGCTCAACCGTGGAGGGTCATTGGAAACTGGGGAACTT

GAGTGCAGAAGAGGAAAGTGGAATTCCAAGTGTAGCGGTGAAATGCGTAGAGATTTGGAGGAACACCAGT

GGCGAAGGCGACTTTCTGGTCTGTAACTGACACTGAGGCGCGAAAGCGTGGGGAGCAAACAGGATTAGAT

ACCCTGGTAGTCCACGCCGTAAACGATGAGTGCTAAGTGTTAGAGGGTTTCCGCCCTTTAGTGCTGCAGC

TAACGCATTAAGCACTCCGCCTGGGGAGTACGGTCGCAAGACTGAAACTCAAAGGAATTGACGGGGGCCC

GCACAAGCGGTGGAGCATGTGGTTTAATTCGAAGCAACGCGAAGAACCTTACCAGGTCTTGACATCCTCT

GACAACCCTAGAGATAGGGCGTTCCCCTTCGGGGGACAGAGTGACAGGTGGTGCATGGTTGTCGTCAGCT

CGTGTCGTGAGATGTTGGGTTAAGTCCCGCAACGAGCGCAACCCTTGATCTTAGTTGCCAGCATTCAGTT

GGGCACTCTAAGGTGACTGCCGGTGACAAACCGGAGGAAGGTGGGGATGACGTCAAATCATCATGCCCCT

TATGACCTGGGCTACACACGTGCTACAATGGACGGTACAAAGGGCAGCAAAACCGCGAGGTCGAGCCAAT

CCCATAAAACCGTTCTCAGTTCGGATTGCAGGCTGCAACTCGCCTGCATGAAGCCGGAATCGCTAGTAAT

CGCGGATCAGCATGCCGCGGTGAATACGTTCCCGGGCCTTGTACACACCGCCCGTCACACCACGAGAGTT

TGTAACACCCGAAGTCGGTGGGGTAACCTTTATGGAGCCAGCCGCCTAAGGTGGGACAGATGATT

>OL955454.1 Sutcliffiella sp. strain L7-7 16S ribosomal RNA gene, partial sequence

ACGCGGTTTGCGGTCTATCTGCAGTCGAGCGGATGAGAGGAGCTTGCTCCTCTGATTCAGCGGCGGACGG

GTGAGTAACACGTGGGCAACCTGCCCTTGAGACTGGGATAACTTCGGGAAACCGGAGCTAATACCGGATA

ATATTTGGAACCTCCTGGTTCCATATTGAAAGATGGTTTCGGCTATCACTCAAGGATGGGCCCGCGGCGC

ATTAGCTAGTTGGTGAGGTAACGGCTCACCAAGGCAACGATGCGTAGCCGACCTGAGAGGGTGATCGGCC

ACACTGGGACTGAGACACGGCCCAGACTCCTACGGGAGGCAGCAGTAGGGAATCTTCCACAATGGACGAA

AGTCTGATGGAGCAACGCCGCGTGAGTGATGAAGGCCTTCGGGTCGTAAAGCTCTGTTGTTAGGGAAGAA

CAAGTGCGAGAGTAACTGCTCGCACCTTGACGGTACCTAACCAGAAAGCCACGGCTAACTACGTGCCAGC

AGCCGCGGTAATACGTAGGTGGCAAGCGTTATCCGGAATTATTGGGCGTAAAGCGCGCGCAGGTGGTTTC

TTAAGTCTGATGTGAAAGCCCACGGCTCAACCGTGGAGGGTCATTGGAAACTGGGGAACTTGAGTGCAGA

AGAGGAAAGTGGAATTCCAAGTGTAGCGGTGAAATGCGTAGAGATTTGGAGGAACACCAGTGGCGAAGGC

GACTTTCTGGTCTGTAACTGACACTGAGGCGCGAAAGCGTGGGGAGCAAACAGGATTAGATACCCTGGTA

GTCCACGCCGTAAACGATGAGTGCTAAGTGTTAGAGGGTTTCCGCCCTTTAGTGCTGCAGCTAACGCATT

AAGCACTCCGCCTGGGGAGTACGGTCGCAAGACTGAAACTCAAAGGAATTGACGGGGGCCCGCACAAGCG

GTGGAGCATGTGGTTTAATTCGAAGCAACGCGAAGAACCTTACCAGGTCTTGACATCCTCTGACAACCCT

AGAGATAGGGCGTTCCCCTTCGGGGGACAGAGTGACAGGTGGTGCATGGTTGTCGTCAGCTCGTGTCGTG

AGATGTTGGGTTAAGTCCCGCAACGAGCGCAACCCTTGATCTTAGTTGCCAGCATTAAGTTGGGCACTCT

AAGGTGACTGCCGGTGACAAACCGGAGGAAGGTGGGGATGACGTCAAATCATCATGCCCCTTATGACCTG

GGCTACACACGTGCTACAATGGACGGTACAAAGGGCAGCAAAACCGCGAGGTCGAGCCAATCCCATAAAA

CCGTTCTCAGTTCGGATTGCAGGCTGCAACTCGCCTGCATGAAGCCGGAATCGCTAGTAATCGCGGATCA

GCATGCCGCGGTGAATACGTTCCCGGGCCTTGTACACACCGCCCGTCACACCACGAGAGTTTGTAACACC

CGAAGTCGGTGGGGTAACCTTGAGCCAGCCGCTACGGAACAGA

>OL955454.1 Sutcliffiella sp. strain L7-7 16S ribosomal RNA gene, partial sequence

ACGCGGTTTGCGGTCTATCTGCAGTCGAGCGGATGAGAGGAGCTTGCTCCTCTGATTCAGCGGCGGACGG

GTGAGTAACACGTGGGCAACCTGCCCTTGAGACTGGGATAACTTCGGGAAACCGGAGCTAATACCGGATA

ATATTTGGAACCTCCTGGTTCCATATTGAAAGATGGTTTCGGCTATCACTCAAGGATGGGCCCGCGGCGC

ATTAGCTAGTTGGTGAGGTAACGGCTCACCAAGGCAACGATGCGTAGCCGACCTGAGAGGGTGATCGGCC

ACACTGGGACTGAGACACGGCCCAGACTCCTACGGGAGGCAGCAGTAGGGAATCTTCCACAATGGACGAA

AGTCTGATGGAGCAACGCCGCGTGAGTGATGAAGGCCTTCGGGTCGTAAAGCTCTGTTGTTAGGGAAGAA

CAAGTGCGAGAGTAACTGCTCGCACCTTGACGGTACCTAACCAGAAAGCCACGGCTAACTACGTGCCAGC

AGCCGCGGTAATACGTAGGTGGCAAGCGTTATCCGGAATTATTGGGCGTAAAGCGCGCGCAGGTGGTTTC

TTAAGTCTGATGTGAAAGCCCACGGCTCAACCGTGGAGGGTCATTGGAAACTGGGGAACTTGAGTGCAGA

AGAGGAAAGTGGAATTCCAAGTGTAGCGGTGAAATGCGTAGAGATTTGGAGGAACACCAGTGGCGAAGGC

GACTTTCTGGTCTGTAACTGACACTGAGGCGCGAAAGCGTGGGGAGCAAACAGGATTAGATACCCTGGTA

GTCCACGCCGTAAACGATGAGTGCTAAGTGTTAGAGGGTTTCCGCCCTTTAGTGCTGCAGCTAACGCATT

AAGCACTCCGCCTGGGGAGTACGGTCGCAAGACTGAAACTCAAAGGAATTGACGGGGGCCCGCACAAGCG

GTGGAGCATGTGGTTTAATTCGAAGCAACGCGAAGAACCTTACCAGGTCTTGACATCCTCTGACAACCCT

AGAGATAGGGCGTTCCCCTTCGGGGGACAGAGTGACAGGTGGTGCATGGTTGTCGTCAGCTCGTGTCGTG

AGATGTTGGGTTAAGTCCCGCAACGAGCGCAACCCTTGATCTTAGTTGCCAGCATTAAGTTGGGCACTCT

AAGGTGACTGCCGGTGACAAACCGGAGGAAGGTGGGGATGACGTCAAATCATCATGCCCCTTATGACCTG

GGCTACACACGTGCTACAATGGACGGTACAAAGGGCAGCAAAACCGCGAGGTCGAGCCAATCCCATAAAA

CCGTTCTCAGTTCGGATTGCAGGCTGCAACTCGCCTGCATGAAGCCGGAATCGCTAGTAATCGCGGATCA

GCATGCCGCGGTGAATACGTTCCCGGGCCTTGTACACACCGCCCGTCACACCACGAGAGTTTGTAACACC

CGAAGTCGGTGGGGTAACCTTGAGCCAGCCGCTACGGAACAGA
